# Recording of wider determinants of health in UK primary care using the Clinical Practice Research Datalink (CPRD) databases, a descriptive cross-sectional study

**DOI:** 10.3389/fpubh.2026.1788509

**Published:** 2026-05-18

**Authors:** Eleanor L. Axson, Kathleen Kenyatta White, Justin Chan, Alice Hinchliffe, Abbie Hunt, Rachael Williams, Susan Hodgson

**Affiliations:** Clinical Practice Research Datalink, Medicines and Healthcare Products Regulatory Agency, London, United Kingdom

**Keywords:** census, determinants of health, electronic healthcare records, primary care, representation, United Kingdom

## Abstract

**Introduction:**

To explore the recording of wider determinants of health in the Clinical Practice Research Datalink (CPRD) Gold and CPRD Aurum primary care databases (jointly CPRD Combined), and, presented these for description in relation to the UK Census 2011 and UK Census 2021. Wider determinants explored include country of birth, language, religion, sexual orientation, gender identity, care home residence, unpaid carer responsibilities, and relationship status.

**Methods:**

All acceptable, currently registered patients from the CPRD databases on the relevant UK Census dates were included. The proportion of patients with a record pertaining to each wider determinant of health was assessed. The distribution of each characteristic in the CPRD populations was descriptively presented against the distribution of that characteristic in the UK Census populations.

**Results:**

There were 17,232,402 and 16,935,144 acceptable, currently registered patients in CPRD Combined at the time of the 2011 and 2021–2 UK Censuses, respectively. The proportion of CPRD Combined patients in 2021–2 with any codes related to country of birth was 3.65%, to language was 42.92%, to religion was 7.05%, to sexual orientation was 1.46%, to gender identity was 0.11%, to care home residence was 0.43%, to unpaid carer responsibilities was 1.35%, and to legal partnership status was 25.39%. Recording increased between the Censuses. Recording was lower in CPRD Gold compared to CPRD Aurum.

**Conclusions:**

Recording of the wider determinants of health examined in this study was low in primary care across the board. Despite limitations, researchers still use CPRD data to investigate questions on the wider determinants of health because of the sparsity of alternative real world data sources that include the richness of health-related data available in CPRD.

## 1 Introduction

Wider determinants of health are the suite of social, cultural, and environmental characteristics that influence mental and physical health, as well as access and provision of care. Systematic variations in these characteristics result in social inequalities, including in health care (1). There is a need for large population-level, real-world data sources on these wider determinants of health to support investigations into their impact on aspects of health and care. This study aimed to provide a simple overview of the recording of wider determinants of health in United Kingdom (UK) Clinical Practice Research Datalink (CPRD) primary care databases, which has not been done before, to allow greater understanding and serve as the foundation for further research.

The CPRD provides access to anonymised coded electronic healthcare record data from a sample of general practices (GPs) across the UK. Previous studies have shown that both CPRD databases, individually and combined, are largely representative of the UK general population in terms of age, sex, ethnicity, and area socioeconomic status compared with the UK Census 2011 (2–6). Completeness of recording for the characteristics of age, sex, ethnicity, and area socioeconomic status is high in primary care. Previous studies have shown age and sex to be nearly 100% recorded, ethnicity recording to be >80% complete, and area socioeconomic status recording to be >90% complete (2–6).

The ascertainment of data for other wider determinants of health in the GP data is less well studied. Previous research has developed ways of characterizing certain wider determinants of health in the CPRD data, and in the additional data sources linked to CPRD, including Hospital Episode Statistics (HES) and Office for National Statistics (ONS) Death Registration; however, none of these have been validated (7–10). These studies have also found poor completeness of recording for some wider determinants of health; for example, Jain et al. (8), reported completeness of recording of country of birth (0.7% complete), religion (2.6%), type of residence (10.0%), and marital status (27.2%) for adults aged ≥65 years old in CPRD Gold.

Despite difficulties in variable definitions and high levels of missing data, previous studies have used CPRD to investigate the effects of wider determinants of health on diverse topics such as: primary care utilization by migrants before and during the COVID-19 pandemic (11); cancer risk factors among transgender and gender diverse individuals (12); and osteoporosis-related characteristics in care home residents (13).

In this study, we investigated the recording of a range of wider determinants of health in the CPRD databases and compared the distribution of these in CPRD to the UK Censuses. The first aim was to explore the recording of selected wider determinants of health in the CPRD primary care databases. The selected wider determinants of health were chosen from those recorded by the UK Census and which might plausibly be recorded as part of the coded data by a GP during a patient consultation. These included: country of birth, language, religion, sexual orientation, gender identity, care home residence, unpaid carer responsibilities, and relationship status. The second aim was to assess the representativeness of the CPRD Gold and CPRD Aurum databases in terms of these variables when compared to the UK Census 2011 and UK Census 2021–2.

The overall purpose of this study was to provide researchers with information on the completeness and representativeness, where possible, of the CPRD Gold and CPRD Aurum databases in terms of wider determinants and compared to the UK population to inform choice of data sources, interpretation of results, and translation of those results into practice for patients.

## 2 Methods

This study was approved by CPRD's Research Data Governance (RDG) Process (protocol 23_003005).

### 2.1 Primary care data sources

Data in CPRD is curated into two databases from two software systems for general practice records: CPRD Gold, sourcing data from the Vision^®^ software system, and CPRD Aurum, sourcing data from EMIS Web^®^. CPRD Gold and CPRD Aurum can be used either independently or in conjunction. This study utilized data from the September 2023 builds of the CPRD Gold and CPRD Aurum databases (14, 15). Where the populations of CPRD Gold and CPRD Aurum were assessed together, practice-level de-duplication was undertaken to avoid double counting in cases where a practice had migrated to a different software supplier, retaining the most contemporary record.

### 2.2 UK Census data

Population proportions for comparison were drawn from the UK Census 2011 and UK Census 2021–2 (Supplementary material 1.1). The UK Census 2011 took place on 27 March 2011. The UK Census 2021–2 for England, Wales, and Northern Ireland (NI) took place on 21 March 2021. The UK Census 2021–2 for Scotland took place on 20 March 2022. Gender identity and sexual orientation data were not collected in the UK Census 2011 and gender identity was also not collected in the 2021 Census in NI.

### 2.3 Study populations

In this descriptive, cross-sectional study, all research acceptable, currently registered patients (Supplementary material 1.2) from the CPRD databases on the relevant UK Census dates were included. Patients were followed from the latest of their birth date or most recent registration date until their censoring date, which was the earliest of the patient's transfer out/registration end date, the CPRD derived death date, or the last data collection date from their GP. Date of birth was set to 01/07/year of birth for those aged 16 years and over and to 01/month of birth/year of birth for those under 16 years old. Patients were defined as registered and contributing data if their start date was before or equal to, and their censoring date was after, the relevant UK Census date.

### 2.4 Characteristic definition in primary care

Geography (UK, England, NI, Scotland, Wales) and sex were determined using the “region” and “gender” variables in the CPRD database Practice and Patient Tables respectively. Age, continuously in years, was calculated as the date of the relevant UK Census minus the set birth date of the patient.

Each wider determinant of health was ascertained using Read, SNOMED-CT, and software-specific codes (Supplementary material 10) and through the development of an algorithm to identify the most recent data prior to the date of the relevant census. Only codes prior to the relevant Census date were considered. Simple assumptions were made to decide the most relevant code if there were multiple available prior to the relevant Census date (Supplementary material 2–9).

For comparison to Census distributions, patients with no coded information were assigned to the following default categories, which were based on the majority categories from the Census: country of birth was assigned as the UK, language as a UK language, religion as unknown, sexual orientation as unknown or heterosexual (considered both separately as defaults), gender identity as the same as sex assigned at birth, non-care home residence, and no unpaid carer responsibilities. For legal partnership status, persons with unknown status were not included in comparisons to the Census distributions.

Detailed information on the definitions and algorithms to define each characteristic are outlined in the relevant Supplementary Appendices for country of birth (Supplementary material 2.1–2.2), language (Supplementary material 3.1–3.2), religion (Supplementary material 4.1–4.2), sexual orientation (Supplementary material 5.1–5.2), gender identity (Supplementary material 6.1–6.2), care home residence (Supplementary material 7.1–7.2), unpaid carer responsibilities (Supplementary material 8.1–8.2), and relationship status (Supplementary material 9.1–9.2).

As a sensitivity analysis, two definitions of unpaid carer responsibilities were assessed: (1) a strict definition relying on codes explicitly stating that the patient was providing unpaid care, and (2) a broad definition relying on the strict codes as well as non-specific care provision codes (Supplementary material 8, 10.7). The results for the broad definition are presented in the main paper; all results are presented in Supplementary material 8.3.

### 2.5 Characteristic definition in the UK censuses

Each characteristic defined in primary care was mapped to the categories of a corresponding characteristic captured by the UK Census data, where possible. The proportion of the UK population with a given characteristic was determined from the relevant data table for the relevant geography. All UK Census data tables used for comparison are detailed and referenced in Supplementary material 1.1.

### 2.6 Analyses

Completeness of recording for each wider determinant of health was measured using the count and proportion of patients with any record for the given characteristic, including a record of “unknown”. The CPRD population with relevant coding for a given characteristic was descriptively shown against the Census population as described above.

Distribution among categories for each characteristic was reported (*N*, %) along with the proportion of patients (*N*, %) in the CPRD databases with a non-missing/unknown record. Distributions were stratified by geography (UK, England, NI, Scotland, and Wales), where appropriate. Further stratifications and statistical tests of significance were not undertaken due to the large amount of missingness.

## 3 Results

There were 6,936,975 acceptable, currently registered patients in CPRD Gold at the time of the 2011 UK Census. There were 12,109,773 acceptable, currently registered patients in CPRD Aurum at the time of the 2011 England and NI Censuses. After deduplication, there were 17,232,402 acceptable, currently registered patients in CPRD Combined at the time of the 2011 UK Census.

There were 3,368,293 acceptable, currently registered patients in CPRD Gold at the time of the 2021–2 UK Censuses. There were 13,578,103 acceptable, currently registered patients in CPRD Aurum at the time of the 2021 England and NI Censuses. After deduplication, there were 16,935,144 acceptable, currently registered patients in CPRD Combined at the time of the 2021–2 UK Censuses.

The representativeness of the CPRD databases to the UK and constituent countries in relation to the UK Censuses 2011 and 2021–2 in terms of age, sex, ethnicity, and socioeconomic status have been previously published (2–6, 16) and are not presented again here.

Table 1 is a summary table presenting the proportion of the relevant CPRD populations with any relevant coding for a given Census-comparable characteristic.

**Table 1:** Proportion of the relevant CPRD populations with any codes—including non-specific and unknown—related to the given Census-comparable characteristic on the dates of the United Kingdom (UK) Census 2011 and UK Census 2021–2.

Census-comparable characteristic	CPRD combined (UK)	CPRD Gold (UK)	CPRD Aurum (ENI only)
Proportion of patients with a relevant code at the time of UK Census 2011			
Country of birth	1.46%	0.48%	1.88%
Language	31.61%	8.96%	41.20%
Religion	3.81%	1.21%	4.91%
Care home Residence	0.25%	0.26%	0.22%
Unpaid care	0.61%	0.62%	0.63%
Legal partnership	16.34%	30.77%	14.92%
Proportion of patients with a relevant code at the time of UK Census 2021–2			
Country of birth	3.65%	0.98%	1.31%
Language	42.92%	6.06%	52.07%
Religion	7.05%	1.02%	8.54%
Sexual orientation	1.46%	0.30%	1.76%
Gender identity	0.11% (ESW only)	0.10% (ESW only)	0.11% (E only)
Care home residence	0.43%	0.28%	0.47%
Unpaid care	1.35%	1.51%	1.34%
Legal partnership	25.39%	34.06%	23.21%

See Supplementary material 2–9 for details on how each characteristic and relevant population were defined. The UK includes England (E), Northern Ireland (NI), Scotland (S), and Wales (W).

### 3.1 Country of birth

Country of birth was recorded for 1.46% of the CPRD population in 2011 and 3.65% of the population in 2021–2 (Table 1; Supplementary material 2.3). The proportion of the CPRD populations born outside of the UK was smaller than the proportions reported in both Censuses, though proportion of patients in the CPRD data with country of birth recorded did increase between the Censuses. Full results for country of birth by UK country as recorded in CPRD data in comparison to the Census, where possible, are detailed in Supplementary material 2.4.

### 3.2 Language

Language was recorded for 31.61% and 42.92% of the CPRD Combined population aged ≥3 years old in 2011 and 2021–2, respectively (Table 1; Supplementary material 3.3); however, most of those records were for explicitly coded UK languages, with 28.34% and 18.53% of all language codes being for UK languages (including UK sign languages, English, Welsh, Gaelic, Irish, Scots, Manx, Cornish, and Romany English) in 2011 and 2021–2, respectively. Language recording was higher in CPRD Aurum than in CPRD Gold. Recording of language increased between the Censuses, but the proportion of the population with non-UK languages recorded was lower than the proportions seen in both Censuses (Table 2).

**Table 2:** Proportion of the CPRD population with a non-United Kingdom (UK) language code(s) in each country compared to the proportion of the UK Census population in each country with a non-UK main spoken language for 2011 and 2021–2.

Geography	CPRD combined	CPRD Gold	CPRD Aurum	Census population
Proportion of population with non-UK language records at the time of the UK Census 2011				
United Kingdom	3.26%	0.63%	N/A	7.35%
England	3.89%	1.29%	4.40%	7.94%
Northern Ireland	0.12%	0.09%	0.26%	2.90%
Scotland	0.08%	0.08%	N/A	5.55%
Wales	0.12%	0.12%	N/A	2.85%
Proportion of population with non-UK language records at the time of the UK Census 2021–2				
United Kingdom	4.00%	0.52%	N/A	8.40%
England	4.60%	2.12%	4.68%	9.14%
Northern Ireland	0.17%	0.13%	0.35%	4.24%
Scotland	0.31%	0.31%	N/A	5.16%
Wales	0.16%	0.16%	N/A	3.29%

Language was assessed for persons aged ≥3 years old at the time of the given Census. UK languages were the default. The proportion of the CPRD Combined population with any language code was 31.61% in 2011 and 42.92% in 2021–2. See Supplementary material 3 for further information.

### 3.3 Religion

Religion was reported for 3.81% and 7.05% of the CPRD Combined population in 2011 and 2021–2, respectively (Table 1; Supplementary material 4.3). The most recorded religions in CPRD Combined were Christian (1.96% and 3.32%) and Muslim (0.51% and 0.95%) in 2011 and 2021–2. Religion recording was higher in CPRD Aurum than in CPRD Gold. The recording of religion in CPRD data increased between the Censuses but the proportion of the population with religions recorded remained well below those seen in the Censuses. Full results for religion as recorded in CPRD data in comparison to the Census, where possible, are detailed in Supplementary material 4.4. Due to small numbers, description of religion was only undertaken at the largest geography for each database.

### 3.4 Sexual orientation

Sexual orientation was recorded for 0.30%, 1.76%, and 1.46% of the CPRD Gold, CPRD Aurum, and CPRD Combined populations ≥16 years old in 2021–2 (Table 1; Supplementary material 5.3). The most frequently reported sexual orientation was heterosexual, at 0.27% of CPRD Gold, 1.62% of CPRD Aurum, and 1.35% of the CPRD Combined populations. The reporting of sexual orientations other than heterosexual, homosexual, and bisexual represented <0.01% of the CPRD populations, compared to ~0.33% of the Census populations. Full results for sexual orientation as recorded in CPRD data in comparison to the Census, where possible, are detailed in Supplementary material 5.4.

### 3.5 Gender identity

Gender identity was recorded for only 0.11% of the CPRD Combined population aged ≥16 years old in 2021–2 in England, Scotland, and Wales (Table 1; Supplementary material 6.3). Gender identity was not collected by the 2021 Census in NI. The distribution of gender identities in the CPRD data was skewed toward the default assignment of gender identity being the same as sex assigned at birth (Table 3). Due to small numbers, description of gender identity was only undertaken at the largest geography for each database.

**Table 3:** Proportion of the CPRD Combined, CPRD Gold, and CPRD Aurum populations in England (E), Scotland (S), and Wales (W) with a given gender identity compared to the proportion of the ESW Census population with a given gender identity for 2021–2.

Gender identity	CPRD combined (ESW)	CPRD Gold (ESW)	CPRD Aurum (E only)	Census 2021–2 (ESW)
Same sex as birth [default]	99.90%	99.90%	99.90%	93.46%
Transgender	0.03%	0.03%	0.03%	0.20%
Other/unknown	0.07%	0.07%	0.07%	6.34%

Gender identity was assessed for persons aged ≥16 years old at the time of the given Census. Default category was same sex as birth. ‘Other/Unknown' included non-specified gender identity, non-binary, all other gender identities, and not answered (Census only). The proportion of the CRPD Combined population with a gender identity code was 0.11% in ESW in 2021–2. Gender identity was not collected by the 2021 Census in Northern Ireland. Due to small numbers, description of gender identity was only undertaken at the largest geography for each database. Please see Supplementary Appendix 6 for further information.

### 3.6 Care home residence

Care home residence was recorded for 0.25% and 0.43% of the CPRD Combined population at the time of the 2011 and 2021–2 Censuses, respectively (Table 1; Supplementary material 7.3). The proportions of the CPRD populations recorded as residing in a care home were much lower than those reported in the Censuses. While the proportion of population recorded as residing in a care home in the CPRD data increased in 2021–2, it was still much lower than that reported in the Census. A smaller proportion of the CPRD populations had records of residing in a care home compared to the Census populations (Table 4).

**Table 4:** Proportion of the CPRD Combined, CPRD Gold, and CPRD Aurum populations in the given geographies with a record of living in a care home compared to the United Kingdom (UK) Censuses 2011 and 2021–2.

Geography	CPRD combined	CPRD Gold	CPRD Aurum	Census population
Proportion of population living in a care home at the time of the UK Census 2011				
United Kingdom	0.19%	0.20%	N/A	0.68%
England	0.18%	0.18%	0.17%	0.68%
Northern Ireland	0.38%	0.42%	0.19%	0.57%
Scotland	0.19%	0.19%	N/A	0.68%
Wales	0.26%	0.26%	N/A	0.70%
Proportion of population living in a care home at the time of the UK Census 2021–2				
United Kingdom	0.39%	0.26%	N/A	0.58%
England	0.41%	0.19%	0.42%	0.57%
Northern Ireland	0.57%	0.62%	0.36%	0.70%
Scotland	0.16%	0.19%	N/A	0.57%
Wales	0.35%	0.35%	N/A	0.63%

Default category was not living in a care home. Care home residence was recorded for 0.25% and 0.43% of the CPRD Combined population at the time of the 2011 and 2021–2 Censuses, respectively. Please see Supplementary Appendix 7 for further information.

### 3.7 Unpaid carer responsibilities

According to the UK Census 2011, 10.30% of UK usual residents aged ≥5 years old provided at least 1 h of unpaid care per week; however, that proportion of patients with a coded record of unpaid carer responsibilities was only 0.61% in CPRD Combined (Tables 1, 5; Supplementary material 8.3). According to the UK Census 2021, 9.20% of the UK usual residents aged ≥5 years old (≥3 in Scotland) provided at least 1 h of unpaid care per week; however, that proportion was only 1.35% in CPRD Combined (Tables 1, 5; Supplementary material 8.3).

**Table 5:** Proportion of the CPRD population providing unpaid care in each country compared to the proportion of the United Kingdom (UK) Census population in each country providing unpaid care for 2011 and 2021–2.

Geography	CPRD combined	CPRD Gold	CPRD Aurum	Census population
Proportion of the population with unpaid carer responsibilities at the time of the UK Census 2011				
United Kingdom	0.61%	0.62%	N/A	10.30%
England	0.62%	0.65%	0.62%	10.24%
Northern Ireland	0.68%	0.69%	0.71%	11.82%
Scotland	0.59%	0.60%	N/A	9.30%
Wales	0.48%	0.48%	N/A	12.09%
Proportion of population with unpaid carer responsibilities at the time of the UK Census 2021–2				
United Kingdom	1.35%	1.51%	N/A	9.20%
England	1.34%	2.28%	1.56%	8.76%
Northern Ireland	1.82%	1.98%	1.19%	12.42%
Scotland	1.29%	1.29%	N/A	11.86%
Wales	1.40%	1.41%	N/A	10.53%

Unpaid carer responsibilities were assessed for persons aged ≥5 years old at the time of the given Census. Default category was not providing unpaid care. Unpaid carer responsibilities were recorded for 0.61% and 1.35% of the CPRD Combined population aged ≥5 years old (≥3 in Scotland) at the time of the UK Census 2011 and 2021–2, respectively. Please see Supplementary Appendix 8 for further information.

### 3.8 Relationship status

At the time of the UK Census 2011, a relationship status was recorded for 31.53% and a legal partnership status was recorded for 16.34% of the CPRD population aged ≥16 years old. At the time of the UK Census 2021–2, a relationship status was recorded for 47.71% and a legal partnership status was recorded for 25.39% of the CPRD population aged ≥16 years old. Comparisons of the legal partnership distributions from the CPRD data compared to the 2011 and 2021–2 Censuses by country are detailed in Table 6. Full results for relationship status and legal partnerships by country in comparison to the Census, where possible, are detailed in Supplementary material 9.

**Table 6:** Proportion of the CPRD population with a given legal partnership status in each country compared to the proportion of the UK Census population in each country by legal partnership status for 2011 and 2021–2.

Geography	Never married/not in a registered civil partnership		Married/in a registered civil partnership		Separated but still legally married/in a registered civil partnership		Divorced/dissolved civil partnership		Widowed/surviving civil partner	
	CPRD combined	Census 2011	CPRD combined	Census 2011	CPRD combined	Census 2011	CPRD combined	Census 2011	CPRD combined	Census 2011
United Kingdom	46.36%	34.69%	45.20%	46.72%	1.79%	2.72%	2.73%	8.84%	3.93%	7.03%
England	35.81%	34.64%	47.85%	46.82%	3.26%	2.65%	4.31%	8.97%	8.77%	6.91%
Northern Ireland	47.62%	36.14%	45.78%	47.65%	1.77%	3.98%	1.38%	5.45%	3.45%	6.78%
Scotland	56.12%	35.38%	37.53%	45.41%	1.45%	3.22%	1.87%	8.21%	3.03%	7.77%
Wales	38.83%	33.52%	50.85%	46.74%	1.73%	2.18%	3.75%	9.66%	4.84%	7.90%
	**CPRD combined**	**Census 2021–2**	**CPRD combined**	**Census 2021–2**	**CPRD combined**	**Census 2021–2**	**CPRD combined**	**Census 2021–2**	**CPRD combined**	**Census 2021–2**
United Kingdom	42.24%	37.92%	47.08%	44.62%	2.32%	2.30%	3.64%	8.96%	4.71%	6.20%
England	40.40%	37.89%	47.84%	44.65%	2.50%	2.24%	3.99%	9.11%	5.28%	6.12%
Northern Ireland	44.82%	38.07%	48.14%	45.77%	2.07%	3.78%	1.77%	6.02%	3.20%	6.36%
Scotland	50.71%	38.13%	42.33%	44.00%	1.74%	2.45%	2.33%	8.38%	2.90%	7.04%
Wales	39.12%	37.18%	51.54%	43.82%	1.91%	2.05%	3.93%	9.87%	3.50%	7.08%

Legal partnership status was assessed for persons aged ≥16 years old at the time of the given Census. Persons with unknown legal partnership status were excluded. The proportion of the CPRD population with a given legal partnership status was 16.34% in 2011 and 25.39% in 2021–2. Please see Supplementary Appendix 9 for full results.

## 4 Discussion

Overall, recording of wider determinants of health in the CPRD primary care databases was largely incomplete and meaningful comparison to the Censuses to assess representativeness was not possible; therefore, the CPRD distributions, using the default categories, are presented next to the Census distributions for illustrative purposes.

### 4.1 Country of birth

Recording of country of birth was low and a smaller proportion of the CPRD population was recorded as migrant compared to the Census. We did not stratify completeness in recording of country of birth by age or sex due to the high level of missingness, but a previous study found that country of birth was recorded for only 0.7% of the CPRD Gold population aged ≥65 years old (8). Taken together, these data suggest that recording of country of birth has improved over time, increasing as we saw from 1.46% to 3.65% between the 2011 and 2021–2 Censuses in the whole population, but with likely variability in ascertainment by age, and potentially other factors such as temporal migration trends. Migrants experience greater barriers to healthcare than the UK-born population and have unique health risks before, during, and after migration (11, 12). The interplay of hostility toward migration, ethnic inequalities, language barriers, and more all contribute to differing health outcomes between migrants and non-migrants (7, 17, 18). Improved recording of migrant status in primary care would allow healthcare providers and researchers to investigate these inequalities more thoroughly.

### 4.2 Language

Recording of language in the CPRD data was higher than for the other wider determinants; however, most records were for UK languages. The higher recording of language in CPRD Aurum than in CPRD Gold might reflect differences in how the GP software systems present the options to record language to GPs. Patients who are not proficient in English experience greater barriers to healthcare, poorer experiences of care, and worse health outcomes than those who are proficient in English (19, 20). Use of interpreting services has been shown to improve patient outcomes and experiences, but these services are underused in primary care (19). Recording of language in primary care, whether spoken or signed, could allow healthcare providers to more easily and quickly provide interpreting and translation services to patients by knowing before an appointment that these services will be required. These records would also allow researchers to track the use and benefits of services and interventions aimed at improving access to and quality of healthcare.

### 4.3 Religion

Recording of religion was low in the CPRD data but increased between the Censuses. Religious and spiritual beliefs and practices can impact on health behaviors, outcomes, service use and access, and acceptability of and adherence to treatment plans (12). Challenges in cross-cultural communication and understanding can impact how patients interact with healthcare providers including what they choose to disclose and how they choose to disclose their health concerns (12).

### 4.4 Sexual orientation

Recording of sexual orientation was low in the CPRD data. Recording was higher in CPRD Aurum compared to CPRD Gold, which may be a result of the more diverse SNOMED-CT and software-specific codes in EMIS Web^®^ available to GPs to describe sexual orientation. Previous work has estimated that only half of patients with homosexual orientation and even less of those with bisexual orientation have disclosed their orientation to their GP (21). There are several barriers to disclosure of minority sexual orientation including the use of heteronormative language and assumptions, fear of discrimination and non-acceptance, and lack of knowledge (22). There are significant health disparities between sexual minority patients and heterosexual patients (22, 23). Recording sexual orientation could allow healthcare providers to provide better care and researchers to track inequalities and the effectiveness of interventions, but this will require increased trust from patients and understanding from providers.

### 4.5 Gender identity

Recording of gender identity was low in the CPRD data. As with sexual orientation, there were a greater variety of gender identity codes in CPRD Aurum compared to CPRD Gold. A recent study utilized additional data sources, including prescription information and secondary care data, to develop a more comprehensive algorithm than the one used here to determine gender identity in CPRD data, defining a cohort of 3,317 transgender patients; however, even with the additional data it was not possible to determine sex assigned at birth in 33% of cases (9). Barriers to disclosure of transgender or gender diverse identity are numerous, with 70% of transgender people reporting they have experienced transphobia from their primary care provider and 57% of transgender people reporting they avoid seeking healthcare when they are unwell (24). Since the 2021–2 Census, the Office for National Statistics downgraded the Census data collected around gender identity from “accredited official statistics” to “official statistics in development” due to concerns about inconsistency in question design (25). Recent initiatives have recommended improved recording of gender identity in a consistent and inclusive way within the National Health Service's (NHS) information technology systems and in future Censuses, and ensure researchers account for sex and gender in their funding applications and research projects (26–28).

### 4.6 Care home residence

While completeness of care home residence recording was low, it is unlikely that not residing in a care home would be coded frequently, making completeness not as indicative of missingness or misclassification risk as with other characteristics. The proportion of the populations with care home residence coded in primary care increased between 2011 and 2021 but remained below the proportions reported in the Census. Previous studies have used CPRD data to investigate public health questions in care home residents and have often included lower age limits to better define this population (8, 13, 29). Age limits were not applied for these analyses as they are not applied in the Census and age does not preclude someone from residing in a care home. There is epidemiological evidence of a rise in the disability and complexities of care required by care home residents in England and Wales (30). Understanding the needs and care requirements of this population will continue to increase in importance, making the ability of health professionals and public health researchers to define residents of care homes useful to assessing future health care needs.

### 4.7 Unpaid carer responsibilities

Around 10% of the UK population report providing unpaid care in the Censuses and the burden of unpaid care is increasing over time; however, <2% of the CPRD population had unpaid carer responsibilities coded in their primary care record. Carer responsibilities negatively impact the mental and physical health of those providing care and a previous study found 61% of carers need more support to maintain their own health (31, 32). Encouraging regular and standardized recording of carer responsibilities in primary care could aid in the provision of better support and services to carers by GPs and allow researchers to examine the impact of interventions on carer health and wellbeing.

### 4.8 Relationship status

The UK Census collects information on legal partnership status, but not on informal relationship statuses. Medical coding allows for the recording of informal relationship statuses; for example, a person may be legally categorized in the Census as “Never Married/In a Registered Civil Partnership” but be in a long-term committed relationship that can be noted in their primary care record. Recording of a relationship status, legally recognized or not, was available for just over 30% of the CPRD population in 2011 and just under 50% of the CPRD population in 2021–2. The relationship between marital status and health is well established in medical literature with studies routinely showing the benefits to health, quality of life, and longevity for married couples and persons with quality wider relationships (33).

### 4.9 Mechanisms for incomplete recording and potential ways to augment primary care data

There are several potential underlying mechanisms that could contribute to the incomplete recording of wider determinants of health in primary care records. Firstly, these characteristics are unlikely to be recorded in primary care unless they directly impact an individual patients care. Secondly, patients may choose not to share this information for any reason. Thirdly, structural measurement bias could result from at least two mechanisms: (1) the use of free text, which is not available to researchers, for recording instead of medical codes and (2) the structure of specific electronic healthcare software facilitating recording of some determinants over others. This last point may be one reason why recording was often higher in CPRD Aurum, sourcing data from EMIS Web^®^, than in CPRD Gold, sourcing data from sourcing data from Vision^®^. It is not possible to disentangle the effects of these and other mechanisms on the completeness and quality of the recording of these determinants in the data. These mechanisms are likely to affect all electronic healthcare record databases, not just CPRD.

The importance of wider determinants of health in the provision of care, access to care, and quality of care means these characteristics have value in understanding the experience and health of patients (1). Despite limitations in their recording in primary care, researchers still use CPRD data to investigate questions on the wider determinants of health because of the richness of the health-related data available (7–9, 11, 13, 34).

Future exploration to refine and/or validate the algorithms used may improve their capabilities, such as including information from family/household indicators, mother-baby relationships, etc (8). Additional data sources, including primary care prescription and procedure data, linked HES data, and linked ONS Death Registration data, were not considered in these analyses (10). Previous studies have included these additional data sources in their development of algorithms to define wider determinants of health and have found their inclusion to enhance the ability to define and infer some of these characteristics, but not all (7–9).

Additionally, exploration of the feasibility and development of infrastructure to support linkage between primary care and Census data may improve researchers' ability to study wider determinants of health and could reduce the need for routine recording of information in primary care that is not directly relevant to patient care.

### 4.10 Limitations

The primary purpose of the data recorded in electronic medical records is to service patient care, not provide information for research purposes. Coding of certain patient characteristics is neither mandated nor required for patient care. Patient characteristics may be noted in free text records and therefore not available to in the CPRD data for researchers, a potential source of structural measurement bias. Additionally, characteristics are more likely to be recorded if they directly impact the provision of patient care. For example, having a native language other than English would be less likely to be recorded if a person also speaks English without the need for an interpreter.

The geography (UK, England, NI, Scotland, Wales) for each patient available in CPRD is only linked to their most recently recorded geography. If a patient lived in a different geography in the past, such as at the time of either UK Census, this would not be reflected in their current data. These analyses are based on a patient's current geography linked to their historically recorded health data.

These analyses were limited to patients deemed acceptable for research per CPRD's criteria (Supplementary material 1.2). This may have introduced selection bias favoring default categories as temporarily registered patients were excluded; however, the impact was likely minimal as over 98% of patient data in CPRD are research quality data (14, 15).

The “gender” variable in the CPRD Patient tables was assumed to represent sex defined at birth; although it is recognized that this is not always the case. For example, in England, patients who transition and request this to be reflected in their medical record are provided with a new NHS number that corresponds to their affirmed gender and all previous medical history is transferred to that new record (35). The Census data on gender identity is classified as “official statistics in development” due to concerns about inconsistency in question design; however, the data collected remains the most comprehensive, scaled, and accessible data available on gender identity in the UK (25, 28).

Timeliness of recording, i.e., the amount of time between the record and the date of observation, was not considered as part of these analyses and the most recent record prior to the date of observation was taken. Previous research has found that the timeliness of characteristic recording varies greatly and could lead to misclassification of characteristics that vary over time but have not been regularly updated in the records (8).

This study aimed to explore how some of the wider determinants of health are recorded in primary care based on medical observation codes and using simple assumptions, including default categories, to handle missing data. The choice of default categories based on the majority categories from the Census could impact the validity of the distributions presented. Many of the default categories chosen in this descriptive study reflect necessary assumptions that are made by researchers looking to assess a determinant in relation to health using electronic healthcare record data. For example, unless otherwise explicitly coded, gender identity is assumed to match sex assigned at birth. For studies interested in the effects of care home residence or unpaid carer responsibilities, it is necessary to assume that not having an explicit code equates to not being exposed to those effects. These assumptions are not perfect and do result in misclassification that is likely not random. Researchers should be aware of the potential biases introduced and conduct sensitivity analyses appropriate to their study question, such as complete case analysis or analysing missing as a separate category. More sophisticated algorithms and/or inclusion of additional linked data sources might support a more refined analysis in future studies.

### 4.11 Conclusions

Recording of the wider determinants of health examined in this study was low in primary care across the board; however, this data is still used by researchers as it is often the best available to answer their research questions. As a result of the large amount of missingness, representativeness of CPRD compared to the Censuses in relation to these determinants could not be assessed. The primary purpose of medical records is to provide patient care; nevertheless, research using medical records informs healthcare policy, resource distribution, drug and medical device development, and practice standards and guidelines. Linkage of primary care data to data sources, like the Census, that directly record data on these wider determinants could be a route to making these data available for research without adding to the reporting/recording burden of patients and GPs.

## Data Availability

The data analyzed in this study is subject to the following licenses/restrictions: the data that support the findings of this study are available from the Clinical Practice Research Datalink (CPRD), but restrictions apply to the availability of these data, which were used under license for the current study, and so are not publicly available. Requests to access CPRD data are reviewed via the CPRD Research Data Governance (RDG) process to ensure that the proposed research is of benefit to patients and public health. More information is available on the CPRD website: https://www.cprd.com/safeguarding-patient-data. This study utilized data from the September 2023 builds of the CPRD Gold and CPRD Aurum databases (14, 15). Upon reasonable application to the CPRD RDG, researchers may use this information to assemble the data used in this study. For further information, please contact the study authors in the first instance. Requests to access these datasets should be directed to Eleanor Axson, eleanor.axson@mhra.gov.uk.
