# Supplementary material for: Recording of wider determinants of health in UK primary care using the Clinical Practice Research Datalink (CPRD) databases, a descriptive cross-sectional study
Source: Front Public Health. 2026 May 18;14:1788509. doi: 10.3389/fpubh.2026.1788509 (PMC13223126; doi:10.3389/fpubh.2026.1788509)
Supplement: Supplementary file 1 [file Data_Sheet_1.docx]

Supplementary Material

**Table of Contents**

1. General supplementary materials
2. Country of birth
3. Language
4. Religion
5. Sexual orientation
6. Gender identity
7. Care home residence
8. Unpaid carer responsibilities
9. Relationship status
10. Code lists
11. Abbreviations
12. References

# General supplementary materials

## Sources of UK Census Data for Comparison

Identified from the UK Census 2011 and UK Census 2021-2 datasets from the relevant Government sources (see **Table 1.1** for Census Data Sources).

**Table 1.1: Census Data Sources**

| **Data included** | **Data source** | **Table name** |
| --- | --- | --- |
| Country of birth for UK Census 2011 | Nomis/ONS (1) | QS203UK Country of birth |
| Country of birth for EW Census 2021 | Nomis/ONS (2) | TS004 – Country of birth |
| Country of birth for NI Census 2021 | NISRA (3) | MS-A16 Country of birth – basic detail |
| Country of birth for S Census 2022 | Scotland’s Census (4) | UV204 – Country of birth |
| Language for EW Census 2011 | Nomis/ONS(5) | QS204EW Main language (detailed) |
| Language for NI Census 2011 | NISRA (6) | KS207NI Main language |
| Language for S  Census 2011 | Scotland’s Census (7) | Table KS206SC - Language |
| Language for EW Census 2021 | Nomis/ONS (8) | TS024 – Main language |
| Language for NI Census 2021 | NISRA (9) | MS-B13 Main language – full detail |
| Language for S Census 2022 | Scotland’s Census (10) | UV212 - Main language |
| Religion for EW  Census 2011 | Nomis/ONS (11) | QS208EW Religion |
| Religion for NI  Census 2011 | NISRA (12) | KS211NI Religion |
| Religion for S Census 2011 | Scotland’s Census (13) | Table KS209SCb - Religion |
| Religion for EW Census 2021 | Nomis/ONS (14) | TS030 - Religion |
| Religion for NI  Census 2021 | NISRA (15) | MS-B21 Religion – full detail |
| Religion for S Census 2022 | Scotland’s Census (16) | UV205 – Religion |
| Sexual orientation for EW Census 2021 | Nomis/ONS (17) | TS079 – Sexual orientation |
| Sexual orientation for NI Census 2021 | NISRA (18) | MS-C03 Sexual orientation – full detail |
| Sexual orientation for S Census 2022 | Scotland’s Census (19) | UV904 – Sexual orientation |
| Gender identity for EW Census 2021 | Nomis/ONS (20) | TS070 – Gender identity |
| Gender identity for S Census 2022 | Scotland’s Census (21) | UV903a - Trans Status or History (7 Groups) |
| Communal Housing for UK Census 2011 | Nomis/ONS (22) | KS405UK Communal establishment residents |
| Communal housing for EW Census 2021 | Nomis/ONS (23) | TS048 – Communal establishment management and type |
| Communal housing for NI Census 2021 | NISRA (24) | MS-F02 Communal establishment management and type- usual residents in a communal establishment |
| Communal housing for S Census 2022 | Scotland’s Census (25) | UV414 - Communal establishment management and type - Residents |
| Unpaid Care for UK  Census 2011 | Nomis/ONS (26) | QS301UK Provision of unpaid care |
| Unpaid care for EW Census 2021 | Nomis/ONS (27) | TS039 – Provision of unpaid care |
| Unpaid care for NI Census 2021 | NISRA (28) | MS-D17 Provision of unpaid care by broad age bands |
| Unpaid care for S Census 2022 | Scotland’s Census (29) | UV301 – Provision of unpaid care |
| Marital status for UK  Census 2011 | Nomis/ONS (30) | KS103UK Marital and civil partnership status |
| Marital status for EW Census 2021 | Nomis/ONS (31) | TS002 - Legal partnership status |
| Marital and civil partnership status for NI Census 2021 | NISRA (32) | MS-A30: Marital and civil partnership status |
| Marital and civil partnership status for S Census 2022 | Scotland’s Census (33) | UV104 – Marital and civil partnership status |

## Acceptable patient definition in CPRD

Patients in CPRD Gold and CPRD Aurum are labelled as ‘acceptable’ for use in research by a process that identifies and excludes patients with non-continuous follow up or patients with poor data recording that raises suspicion as to the validity of the that patients record. Patient data is checked, for the following issues:

• An empty or invalid first registration date

• An empty or invalid current registration date

• Absence of a record for a year of birth

• A first registration date prior to their birth year

• A current registration date prior to their birth year

• A transferred-out reason with no transferred-out date

• A transferred-out date with no transferred-out reason

• A transferred-out date prior to their first registration date

• A transferred-out date prior to their current registration date

• A current registration date prior to their first registration date

• A gender other than Female/Male/Indeterminate

• An age of greater than 115 at end of follow up

• Recorded health care episodes in years prior to birth year

• All recorded health care episodes have empty or invalid event dates

• Registration status of temporary patients

If any of these conditions are true, then the patient is labelled unacceptable and is not recommended for use in research.

# Country of birth

## Country of birth definitions in CPRD and the Censuses

In accordance with Census information related to country of birth, country of birth was defined for all eligible patients where possible.

Country of birth variable

A categorical variable detailing:

0 = United Kingdom or Uncoded

1. = Europe
2. = Africa
3. = Asia and Middle East
4. = Americas
5. = Australasia
6. = Unknown or Other Country of Birth/Migrant

The default country of birth for those with no relevant coding was the UK. Countries included in the UK category were England, Northern Ireland, Scotland, and Wales.

Countries included in the European category were Albania, Andora, Austria, Azerbaijan, Belgium, Belarus, Bosnia, Bulgaria, Croatia, Cyprus, Czech Republic, Denmark, Estonia, Finland, France, Germany, Greece, Greenland, Georgia, Guernsey, Gibraltar Hungary, Iceland, Ireland, Italy, Isle of Man, Jersey, Kosovo, Latvia, Liechtenstein, Lithuania, Luxembourg, Malta, Moldova, Monaco, Montenegro, Norway, Poland, Portugal, Romania, Russia, San Marino, Slovakia, Slovenia, Spain, Sweden, Switzerland, Turkey, The Netherlands, Ukraine, and the Vatican City.

Countries included in the African category were Algeria, Angola, Benin, Botswana, Burkina Faso, Burundi, Cameroon, Cape Verde, Chad, Comoros, The Congo, The Democratic Republic of Congo, Djibouti, Egypt, Eritrea, Equatorial Guinea, Guinea Bissau, Ethiopia, Gabon, Gambia, Ghana, Ivory Coast, Kenya, Lesotho, Liberia, Libya, Madagascar, Malawi, Mauritania, Mauritius, Mayotte, Morocco, Mozambique, Mali, Namibia, Niger, Nigeria, Rwanda, Reunion Island, Sao Tome and Principe, Senegal, Sierra Leone, Somalia, Sudan, South Sudan, Swaziland (historic data), Seychelles, Tanzania, The Gambia, Tunisia, Togo, Uganda, Western Sahara, Zambia, Zimbabwe

Countries included in the Asia and the Middle East category were Afghanistan, Armenia, Bahrain, Bangladesh, Bhutan, Brunei, Burma, China, Korea, Timor, Hong Kong, India, Indonesia, Iran, Iraq, Israel, Japan, Jordan, Kazakhstan, Kuwait, Kyrgyzstan, Laos, Lebanon, Malaysia, Maldives, Mongolia, Nepal, Oman, Pakistan, Palestine, Philippines, Qatar, Saudi Arabia, Singapore, Sri Lanka, Syria, Taiwan, Tajikistan, Thailand, Turkmenistan, United Arab Emirates, Uzbekistan, Vietnam, Yemen, Christmas Islands, Cocos Islands, Macao, Indian Ocean territory.

Countries included in the Americas category were Argentina, Belize, Bolivia, Brazil, French Guyana, Canada, Chile, Colombia, Costa Rica, Ecuador, El Salvador, Grenada, Guatemala, Honduras, Mexico, Nicaragua, Panama, Paraguay, Peru, Suriname, United States of America, Uruguay, Venezuela, Antigua and Barbuda, The Bahamas, Barbados, Cuba, Dominican Republic, Haiti, Jamaica, Puerto Rico, Saint Kitts and Nevis, Saint Lucia, Saint Vincent and the Grenadines, Trinidad and Tobago, Aruba, United Virgin Islands, Saint Martin, Curacao, Bonaire, Montserrat, Bermuda, Anguilla, British Virgin Islands, Turks and Caicos, Saint Helena, Cayman Islands, Martinique, French Guiana, Falkland Islands, Guadeloupe, Saint Pierre, South Georgia and the Sandwich Islands.

Countries included in the Australasia category were Australia, New Zealand, Fiji, Papua New Guinea, Solomon Islands, Tonga, Tuvalu, Vanuatu, Samoa, Kiribati, Guam, Cook Islands, Micronesia, Marshall Islands, Tokelau, Mariana Islands, Palau, Antarctica, Pitcairn, Wallis and Futuna, French Polynesia, French Southern Territories, and Caledonia.

Country of birth was categorised to be comparable to the 2011 and 2021 Census tables for Country of Birth (1–4).

## Algorithm for determining country of birth in CPRD

For patients who have a code (see 10.1) for a singular country of birth, they were allocated to the corresponding country of birth category.

For patients who had multiple codes for a single country, with an additional migrant or unknown code, they were allocated to the country of birth category which corresponded to their country code.

For patients who had multiple codes for different countries, they were allocated to the unknown category.

The default country of birth for those with no relevant coding was the UK. Countries included in the UK category were England, Northern Ireland, Scotland, and Wales.

## Completeness of country of birth recording in CPRD

Census 2011

On the date of the 2011 Census for the UK, the proportion of the CPRD Gold population with ANY country of birth coding was 0.48% (24,724 out of 5,122,629).

On the date of the 2011 Census for ENI, the proportion of the CPRD Aurum population with ANY country of birth coding was 1.88% (227,260 out of 12,109,773).

On the date of the 2011 Census for UK, the proportion of the CPRD Gold and CPRD Aurum populations combined with ANY country of birth coding was 1.46% (251,989 out of 17,232,402).

Census 2021

On the date of the 2021 Census for the UK, the proportion of the CPRD Gold population with ANY country of birth coding was 0.98% (32,930 out of 3,357,041).

On the date of the 2021 Census for ENI, the proportion of the CPRD Aurum population with ANY country of birth coding was 1.31% (585,023 out of 13,578,103).

On the date of the 2021 Census for UK, the proportion of the CPRD Gold and CPRD Aurum populations combined with ANY country of birth coding was 3.65% (617,953 out of 16,935,144).

## Additional results for country of birth

**Table 2.4.1.** Proportion of the CPRD Combined population born in each world region for each country compared to the proportion of the UK Census population born in each world region for each country for 2011 and 2021.

Born in UK or Uncoded was the default.

| **Geography** | **Born in Africa** | | **Born in Americas or Caribbean** | | **Born in Antarctica, Oceania, Australasia, or Other** | | **Born in Europe (non-UK)** | | **Born in Middle East or Asia** | | **Migrant/Unknown or Other Country of Birth** | | **Born in UK or Uncoded** | |
| --- | --- | --- | --- | --- | --- | --- | --- | --- | --- | --- | --- | --- | --- | --- |
|  | **CPRD** | **Census 2011** | **CPRD** | **Census 2011** | **CPRD** | **Census 2011** | **CPRD** | **Census 2011** | **CPRD** | **Census 2011** | **CPRD** | **Census 2011** | **CPRD** | **Census 2011** |
| **United Kingdom** | 0.26% | 2.16% | 0.14% | 1.13% | 0.05% | 0.31% | 0.42% | 4.76% | 0.38% | 0.56% | 0.21% | N/A | 98.54% | 87.35% |
| **England** | 0.31% | 2.43% | 0.16% | 1.25% | 0.06% | 0.34% | 0.49% | 5.05% | 0.45% | 4.77% | 0.22% | N/A | 98.30% | 86.16% |
| **Northern Ireland** | 0.02% | 0.31% | 0.01% | 0.45% | <0.01% | 0.14% | 0.14% | 4.74% | 0.02% | 0.95% | 0.03% | N/A | 99.78% | 93.41% |
| **Scotland** | 0.01% | 0.88% | 0.01% | 0.65% | 0.01% | 0.23% | 0.08% | 3.25% | 0.03% | 1.97% | 0.28% | N/A | 99.58% | 93.02% |
| **Wales** | 0.01% | 0.72% | <0.01% | 0.34% | <0.01% | 0.14% | 0.03% | 2.40% | 0.01% | 1.89% | 0.04% | N/A | 99.90% | 94.51% |
|  |  |  |  |  |  |  |  |  |  |  |  |  |  |  |
|  | **CPRD** | **Census 2021** | **CPRD** | **Census 2021** | **CPRD** | **Census 2021** | **CPRD** | **Census 2021** | **CPRD** | **Census 2021** | **CPRD** | **Census 2021** | **CPRD** | **Census 2021** |
| **United Kingdom** | 0.51% | 2.49% | 0.30% | 1.26% | 0.07% | 0.32% | 1.26% | 6.73% | 1.18% | 5.23% | 0.33% | N/A | 96.35% | 83.96% |
| **England** | 0.62% | 2.75% | 0.36% | 1.37% | 0.08% | 0.33% | 1.50% | 7.16% | 1.42% | 5.74% | 0.30% | N/A | 95.74% | 82.65% |
| **Northern Ireland** | 0.05% | 0.57% | 0.02% | 0.53% | 0.01% | 0.16% | 0.37% | 5.85% | 0.06% | 1.54% | 0.04% | N/A | 99.45% | 91.35% |
| **Scotland** | 0.05% | 1.33% | 0.03% | 0.91% | 0.01% | 0.26% | 0.19% | 4.63% | 0.08% | 3.01% | 0.81% | N/A | 98.82% | 89.80% |
| **Wales** | 0.03% | 0.92% | 0.01% | 0.40% | 0.01% | 0.16% | 0.07% | 3.22% | 0.05% | 2.23% | 0.20% | N/A | 99.63% | 93.07% |

**Table 2.4.2.** Proportion of the CPRD Gold population born in each world region for each country compared to the proportion of the UK Census population born in each world region for each country for 2011 and 2021.

Born in UK or Uncoded was the default.

| **Geography** | **Born in Africa** | | **Born in Americas or Caribbean** | | **Born in Antarctica, Oceania, Australasia, or Other** | | **Born in Europe (non-UK)** | | **Born in Middle East or Asia** | | **Migrant/Unknown or Other Country of Birth** | | **Born in UK or Uncoded** | |
| --- | --- | --- | --- | --- | --- | --- | --- | --- | --- | --- | --- | --- | --- | --- |
|  | **CPRD Gold** | **Census 2011** | **CPRD Gold** | **Census 2011** | **CPRD Gold** | **Census 2011** | **CPRD Gold** | **Census 2011** | **CPRD Gold** | **Census 2011** | **CPRD Gold** | **Census 2011** | **CPRD Gold** | **Census 2011** |
| **United Kingdom** | 0.06% | 4.34% | 0.03% | 2.69% | 0.01% | 0.84% | 0.15% | 15.43% | 0.09% | 9.59% | 0.15% | N/A | 99.52% | 87.35% |
| **England** | 0.11% | 2.43% | 0.05% | 1.25% | 0.02% | 0.34% | 0.24% | 5.05% | 0.18% | 4.77% | 0.12% | N/A | 99.27% | 86.16% |
| **Northern Ireland** | 0.02% | 0.31% | 0.01% | 0.45% | <0.01% | 0.14% | 0.10% | 4.74% | 0.02% | 0.95% | 0.04% | N/A | 99.82% | 93.41% |
| **Scotland** | 0.01% | 0.88% | 0.01% | 0.65% | 0.01% | 0.23% | 0.08% | 3.25% | 0.03% | 1.97% | 0.28% | N/A | 99.58% | 93.02% |
| **Wales** | 0.01% | 0.72% | <0.01% | 0.34% | <0.01% | 0.14% | 0.03% | 2.40% | 0.02% | 1.89% | 0.04% | N/A | 99.90% | 94.51% |
|  |  |  |  |  |  |  |  |  |  |  |  |  |  |  |
|  | **CPRD Gold** | **Census 2021** | **CPRD Gold** | **Census 2021** | **CPRD Gold** | **Census 2021** | **CPRD Gold** | **Census 2021** | **CPRD Gold** | **Census 2021** | **CPRD Gold** | **Census 2021** | **CPRD Gold** | **Census 2021** |
| **United Kingdom** | 0.07% | 2.49% | 0.03% | 1.26% | 0.01% | 0.32% | 0.25% | 6.73% | 0.12% | 5.23% | 0.49% | N/A | 99.02% | 83.96% |
| **England** | 0.27% | 2.75% | 0.08% | 1.37% | 0.03% | 0.33% | 0.86% | 7.16% | 0.51% | 5.74% | 0.30% | N/A | 97.95% | 82.65% |
| **Northern Ireland** | 0.04% | 0.57% | 0.02% | 0.53% | 0.01% | 0.16% | 0.35% | 5.85% | 0.06% | 1.54% | 0.05% | N/A | 99.46% | 91.35% |
| **Scotland** | 0.05% | 1.33% | 0.03% | 0.91% | 0.01% | 0.26% | 0.19% | 4.63% | 0.08% | 3.01% | 0.81% | N/A | 98.82% | 89.80% |
| **Wales** | 0.03% | 0.92% | 0.01% | 0.40% | 0.01% | 0.16% | 0.07% | 3.22% | 0.05% | 2.23% | 0.20% | N/A | 99.63% | 93.07% |

**Table 2.4.3.** Proportion of the CPRD Aurum population born in each world region for each country compared to the proportion of the UK Census population born in each world region for each country for 2011 and 2021.

Born in UK or Uncoded was the default.

| **Geography** | **Born in Africa** | | **Born in Americas or Caribbean** | | **Born in Antarctica, Oceania, Australasia, or Other** | | **Born in Europe (non-UK)** | | **Born in Middle East or Asia** | | **Migrant/Unknown or Other Country of Birth** | | **Born in UK or Uncoded** | |
| --- | --- | --- | --- | --- | --- | --- | --- | --- | --- | --- | --- | --- | --- | --- |
|  | **CPRD Aurum** | **Census 2011** | **CPRD Aurum** | **Census 2011** | **CPRD Aurum** | **Census 2011** | **CPRD Aurum** | **Census 2011** | **CPRD Aurum** | **Census 2011** | **CPRD Aurum** | **Census 2011** | **CPRD Aurum** | **Census 2011** |
| **England** | 0.35% | 2.43% | 0.18% | 1.25% | 0.06% | 0.34% | 0.54% | 5.05% | 0.51% | 4.77% | 0.24% | <0.01% | 98.12% | 86.16% |
| **Northern Ireland** | 0.01% | 0.31% | <0.01% | 0.45% | <0.01% | 0.14% | 0.36% | 4.74% | 0.01% | 0.95% | <0.01% | <0.01% | 99.59% | 93.41% |
|  |  |  |  |  |  |  |  |  |  |  |  |  |  |  |
|  | **CPRD Aurum** | **Census 2021** | **CPRD Aurum** | **Census 2021** | **CPRD Aurum** | **Census 2021** | **CPRD Aurum** | **Census 2021** | **CPRD Aurum** | **Census 2021** | **CPRD Aurum** | **Census 2021** | **CPRD Aurum** | **Census 2021** |
| **England** | 0.63% | 2.75% | 0.36% | 1.37% | 0.08% | 0.33% | 1.52% | 7.16% | 1.45% | 5.74% | 0.30% | N/A | 95.67% | 82.65% |
| **Northern Ireland** | 0.07% | 0.57% | 0.02% | 0.53% | 0.01% | 0.16% | 0.44% | 5.85% | 0.07% | 1.54% | <0.01% | N/A | 99.39% | 91.35% |

# Language

## Language definitions in CPRD and the Censuses

In accordance with Census information related to main spoken language, language was defined for all eligible patients aged ≥3 years old where possible.

Language variables

A categorical variable detailing:

0 = English

1 = Americas

2 = Africa

3 = Australasia

4 = East Asia

5 = European

6 = Sign language

7 = Other UK languages (non-English)

8 = West and Central Asia

9 = Other/Non-specific languages

The default language for those with no relevant coding was English.

A binary variable detailing:

0 = UK languages including UK sign languages (British Sign Language, Makaton, visual frame sign language), English, Welsh, Gaelic, Irish, Scots, Manx, Cornish, and Romany English

1 = non-UK languages

The default language for those with no relevant coding was UK languages including UK sign languages.

Languages in the Americas category were: Creole, Patois, Quechua, Guarani, Aymara, Inuktitut, Inupiqua, Cree, Haitian, Navajo, Ojibwa, Central South American Indian Language, Algonkian, Mayan, Chinantec, Tanoan, and Kwakiutl.

Languages in the African category were: Amharic, Tigrinya, Somali, Krio, Akan, Yoruba, Igbo, Kiswahili, Twi, Zulku, Kinyarwanda, Luba Katanga, Venda, Luganda, Lingala, Shona, Afrikaans, Ethiopian, Brawa, Barawa, Hausa, Oromo, Afar, Sudanese, Xhosa, Southern Sotho, Tsonga, Malagasy, Tswana, Bamun, Ndebele, Swati, Sango, Rundi, Bamoun, Nyanja, Chewa, Bambara, Ewe, Fulah, Heroro, Hanuri, Kikuyu, Kongo, Kuanyama, Ndonga, Berber, Tamazight, Sidamo, Egyptian, Ethiopic, Harari, Tigre, Central Sudanic, Madi, Nilotic, Luo, Nuer, Fur, Bushman, Maba, Niger, Banda, Bembe, Duala, Ganda, Sotho, Swazi, Yao, Efik, Ibibio, Tiv, Ijo, Western Sudanic, Kwa, Bassa, Edo, Fanti, Ga, Ibo, Nupe, Urhodo, Mande, Malinke, Soninke, Fulani, Temne, and Pidgin.

Languages included in the Australasian category were: Tongan, Māori, Fijian, Samoan, Nauru, Bislama, Chamorro, Hiri Motu, Marshallese, Tahitian, Australian Native, and Enga.

Languages included in the East Asian category were: Mandarin, Chinese, Cantonese, Japanese, Korean, Vietnamese, Thai, Malay, Tagalog, Filipino, Zhuang, Lahu, Hakka, Burmese, Mongolian, Indonesian, Lao, Uighur, Javanese, Central Khmer, Tetum, Sichuan Yi, Manchu, Igorot, Madurese, Pampangan, Visaya, Khmer, Tai, Shan, Chin, Kachin and Karen.

Languages included in the European category were: French, Portuguese, Spanish, Italian, German, Polish, Slovak, Czech, Romanian, Lithuanian, Latvian, Walloon, Hungarian, Bulgarian, Greek, Dutch, Swedish, Danish, Finnish, Estonian, Slovenian, Sami, Albanian, Ukrainian, Bosnian, Croatian, Montenegrin, Romani, Yiddish, Russian, Occitan, Sardinian, Serbo-Croatian, Macedonian, Norwegian, Flemish, Maltese, Azerbaijan, Georgian, Romansh, Basque, Catalan, Moldavian, Kalaallisut, Icelandic, Faeroese, Galician, Belarussian, Corsican, Greenlandic, Breton, Abkhazian, Bashkir, Frisian, Aragonese, Tatar, Avaric, Chechen, Church Slavic, Chuvash, Faroese, Komi, Latin, Limburgan, Luxembourgish, Ossetian, Romany, Romsky, Romanese, Romanes, Balkar, Dargin, Baltic, Luxembourgian, Hellenic, Romance, Rumian, Finnic, Croat, Abkhaz, and Serbian.

Languages included in the Sogn Language category were: unspecified sign languages, moving frame sign language, Makaton, British Sign Language, Australian Sign Language, and American Sign Language.

Languages included in the South Asian category were: Urdu, Hindi, Punjabi, Pahari, Mirpuri, Potwari, Bengali, Sylheti, Chatgaya, Gujurati, Marathi, Gujerati, Teluga, Tamil, Malayalam, Sinhala, Nepalese, Iba, Kutchi, Tibetan, Kannada, Sindhi, Sinhalese, Kashmiri, Oriya, Bihari, Konkani, Dzongkha, Assamese, Hindiko, Hindko, Dhivehi, Pali, Sanskrit, Hinko, Kanarese, Kurukh, Tulu, Hindustani, Maldivian, Baluchi and Khasi.

Languages included in the Other UK Languages (non-English) category were: Welsh, Gaelic, Irish, Scots, Manx, Cornish, and Romany English.

Languages included in the West and Central Asian Category were: Turkish, Arabic, Hebrew, Kurdish, Persian, Farsi, Pashto, Uzbek, Pashtu, Armenian, Turkmen, Kazakh, Dari, Tajik, Kirghiz, Avestan, Pushto, Aramaic, Assyrian, Syriac, and Iranian.

Languages included in the Other/Non-Specific category were those coded with Afro-Asiatic language, Indo-European Language, Italic Language, Indo-Iranian Language, and those with a code for additional main language.

Language included in the English category was English.

UK Languages in CPRD were defined as UK sign languages (British Sign Language, Makaton, visual frame sign language), English, Welsh, Gaelic, Irish, Scots, Manx, Cornish, and Romany English.

Language was categorised to be comparable to the 2011 and 2021 Census tables for Main Spoken Language or Language Used at Home (Scotland only) (5–9).

- UK Languages for the Census data pertaining to the UK, England, and Wales were defined as UK sign languages (British Sign Language, Makaton, visual frame sign language), English, Welsh, Gaelic, Irish, Scots, Manx, Cornish, and Romany English.
- UK Languages for the Census data pertaining to Northern Ireland were English and Irish (Gaelic) in 2011. UK Languages for the Census data pertaining to Northern Ireland were English, Irish, British Sign Language, Ulster-Scots, Irish Sign Language, Gaelic, Welsh, and Scots in 2021.

UK Languages for the Census data pertaining to Scotland were English, Gaelic, Scots, and British Sign Language.

Language was categorised to be comparable to the 2011 and 2021 Census tables for Main Language (5–10).

## Algorithm for determining language in CPRD

For individuals with one code (see 10.2) for a language, they were allocated to the corresponding language category.

For individuals with a code which indicates an undefined language but an additional code for a specific language, they were assigned to the category indicated by the meaningful language code.

For individuals with multiple different language codes, the code which was recorded closest to the date of the census was used to assign the individuals to a language category.

## Completeness of language recording in CPRD

Census 2011

On the date of the 2011 Census for the UK, the proportion of the CPRD Gold population aged ≥3 years old with ANY language coding was 8.96% (442,719 out of 4,942,071).

On the date of the 2011 Census for ENI, the proportion of the CPRD Aurum population aged ≥3 years old with ANY language coding was 41.20% (4,805,275 out of 11,662,012).

On the date of the 2011 Census for UK, the proportion of the CPRD Gold and CPRD Aurum populations combined aged ≥3 years old with ANY language coding was 31.61% (5,247,994 out of 16,604,083).

Census 2021

On the date of the 2021 Census for the UK, the proportion of the CPRD Gold population aged ≥3 years old with ANY language coding was 6.06% (198,052 out of 3,270,374).

On the date of the 2021 Census for ENI, the proportion of the CPRD Aurum population aged ≥3 years old with ANY language coding was 52.07% (6,855,723 out of 13,165,748).

On the date of the 2021 Census for UK, the proportion of the CPRD Gold and CPRD Aurum populations combined aged ≥3 years old with ANY language coding was 42.92% (7,053,775 out of 16,436,174).

# Religion

## Religion definitions in CPRD and the Censuses

In accordance with Census information related to religion, religion characteristics were defined for all eligible patients where possible.

The following religion characteristics were defined:

1. = No religion
2. = Christian
3. = Buddhist
4. = Hindu
5. = Jewish
6. = Muslim
7. = Sikh
8. = Other Religion
9. = Unknown

The default status was defined as unknown.

Codes which were included in the No religion category were: Agnostic, Atheist, non-believer, Humanist and, no religion.

Religions included in the Christian category were: Christian, Church of Scotland, Church of England, Church of Ireland, Catholic, Free Church, Evangelical, Mormon, Jehovah’s Witness, Methodist, Presbyterian, Baptist, Greek Orthodox church, Romanian Orthodox church, Armenian Orthodox church, Russian Orthodox church, Bulgarian Orthodox church, Ethiopian Orthodox church, Ukrainian Orthodox church, Syrian Orthodox church, Coptic Orthodox church, Indian Orthodox church, Anglican, Pentecostal, Christian Brethren, Salvation Army, Moravian, Adventist, Lutheran, United Reformed Church, Unitarian, Episcopal, Christadelphian, Nonconformist Church, Krishnan Church, Mennonite, Church of God Prophecy, New Testament, Nazarene, Plymouth Brethren, Seventh Day Adventist, Unitarian, Congregationalist, Anabaptist, Ethiopian Orthodox church, Quaker, Serbian Orthodox church, Eastern orthodox church, Evangelical, Pietist Religion, Chapel Religion, Swedenborgian, Church of Wales, Church of God Prophecy, Uniting Church, Oriental Orthodox church, Assyrian church, Church of Jesus Christ of Latter Day Saints, Amish, Judaic, French Protestant, and Uniate Catholic.

Religions included in the Buddhist category were: Buddhist, Nichiren Buddhist, Theravada Buddhist, New Kapampa tradition Buddhist, Mahayana Buddhist, Zen Buddist, Tibetan Buddhist, and Lamaist Religion.

Religions included in the Hindu category were: Hindu, Vaishanava Hindu, Arya Samaj Hindu, Smarta Hindu, Shiva Hindu, Advaitin Hindu, Krishna consciousness, and Sanarana Dharma.

Religions included in the Jewish category were: Jew, Hasidic Jew, Haredi Jew, Masorti Jew, Messianic Jew, Ashkenazi Jew, and British Israelite Jew.

Religions included in the Muslim category were: Muslim, Islam, Shiite Muslim, Sunni Muslim, Sufi Muslim, Ahmadi Muslim, Ismaili Muslim, and Druze Muslim.

Religions included in the Sikh category were: Sikh and Sikh Punjabi.

Religions included in the Other Religion category were: Spiritualism, spirituality, pagan, Wicca, Heathen, Jain, Taoist, Rastafarian, Druid, Deis, Shaman, Zoroastrian, Shinto, Universalist, Yoruba religion, Mixed religion, Native American Religion, Religion Not otherwise specified, African religion, Arcane School religion, Arminianist religion, Babis religion, Bahais religion, Calvinist religion, Confucian religion, Eckanker, Eminist, Ismailis, Primal Society religion, Brahma Kumari, Deist, Kabbalist, Peyotist, Pantheist, Santerian, Secularist, Shumeist, Scientologist, Anthroposophist, Asartruar, Black Magic, Light worker, Radha Soami, Infinite way, ancestral worship, and Chondogyo.

Codes included in the Unknown category were religion not recorded and mixed religion.

Religion was categorised to be comparable to the 2011 and 2021 Census tables for Religion (11–15).

- Religion in Northern Ireland was categorised as Christian, Other, no religion, and not stated in 2011.

## Algorithm for determining religion in CPRD

For individuals with one code (see 10.3) for a religion, they were allocated to the corresponding religion category.

For individuals with a code which indicates no answer or unknown religion but an additional code for a specified religion, they were assigned to the category indicated by the specified religion code.

For individuals with multiple different religion codes, the code which was recorded closest to the date of the census was used to assign the individuals to a religion category.

## Completeness of religion recording in CPRD

Census 2011

On the date of the 2011 Census for the UK, the proportion of the CPRD Gold population with ANY religion coding was 1.21% (61,963 out of 5,122,629).

On the date of the 2011 Census for ENI, the proportion of the CPRD Aurum population with ANY religion coding was 4.91% (594,920 out of 12,109,773).

On the date of the 2011 Census for UK, the proportion of the CPRD Gold and CPRD Aurum populations combined with ANY religion coding was 3.81% (656,883 out of 17,232,402).

Census 2021

On the date of the 2021 Census for the UK, the proportion of the CPRD Gold population with ANY religion coding 1.02% (34,160 out of 3,357,041).

On the date of the 2021 Census for ENI, the proportion of the CPRD Aurum population with ANY religion coding 8.54% (1,159,510 out of 13,578,103).

On the date of the 2021 Census for UK, the proportion of the CPRD Gold and CPRD Aurum populations combined with ANY religion coding 7.05% (1,193,670 out of 16,935,144).

## Additional results for religion

**Table 4.4.1.** Proportion of the CPRD Combined population with a given religion compared to the proportion of the UK Census population with a given religion for 2011 and 2021.

No religion/Unknown was the default. Due to small numbers, description of religion was only undertaken at the largest geographies for each database.

| **Geography** | **Buddhist** | | **Christian** | | **Hindu** | | **Jewish** | | **Muslim** | | **Sikh** | | **Other religion** | | **No religion/ Unknown** | |
| --- | --- | --- | --- | --- | --- | --- | --- | --- | --- | --- | --- | --- | --- | --- | --- | --- |
|  | **CPRD** | **Census 2011** | **CPRD** | **Census 2011** | **CPRD** | **Census 2011** | **CPRD** | **Census 2011** | **CPRD** | **Census 2011** | **CPRD** | **Census 2011** | **CPRD** | **Census 2011** | **CPRD** | **Census 2011** |
| **United Kingdom** | 0.03% | 0.41% | 1.96% | 59.49% | 0.17% | 1.32% | 0.05% | 0.43% | 0.51% | 4.40% | 0.07% | 0.68% | 0.05% | 0.43% | 97.17% | 32.84% |
|  |  |  |  |  |  |  |  |  |  |  |  |  |  |  |  |  |
|  | **CPRD** | **Census 2021** | **CPRD** | **Census 2021** | **CPRD** | **Census 2021** | **CPRD** | **Census 2021** | **CPRD** | **Census 2021** | **CPRD** | **Census 2021** | **CPRD** | **Census 2021** | **CPRD** | **Census 2021** |
| **United Kingdom** | 0.05% | 0.43% | 3.32% | 46.53% | 0.31% | 1.59% | 0.11% | 0.41% | 0.95% | 5.97% | 0.10% | 0.80% | 0.09% | 0.58% | 93.29% | 43.67% |

**Table 4.4.2.** Proportion of the CPRD Gold population with a given religion compared to the proportion of the UK Census population with a given religion for 2011 and 2021.

No religion/Unknown was the default. Due to small numbers, description of religion was only undertaken at the largest geographies for each database.

| **Geography** | **Buddhist** | | **Christian** | | **Hindu** | | **Jewish** | | **Muslim** | | **Sikh** | | **Other religion** | | **No religion/ Unknown** | |
| --- | --- | --- | --- | --- | --- | --- | --- | --- | --- | --- | --- | --- | --- | --- | --- | --- |
|  | **CPRD Gold** | **Census 2011** | **CPRD Gold** | **Census 2011** | **CPRD Gold** | **Census 2011** | **CPRD Gold** | **Census 2011** | **CPRD Gold** | **Census 2011** | **CPRD Gold** | **Census 2011** | **CPRD Gold** | **Census 2011** | **CPRD Gold** | **Census 2011** |
| **United Kingdom** | 0.01% | 0.41% | 0.62% | 59.49% | 0.03% | 1.32% | 0.01% | 0.43% | 0.16% | 4.40% | 0.02% | 0.68% | 0.03% | 0.43% | 99.12% | 32.84% |
|  |  |  |  |  |  |  |  |  |  |  |  |  |  |  |  |  |
|  | **CPRD Gold** | **Census 2021** | **CPRD Gold** | **Census 2021** | **CPRD Gold** | **Census 2021** | **CPRD Gold** | **Census 2021** | **CPRD Gold** | **Census 2021** | **CPRD Gold** | **Census 2021** | **CPRD Gold** | **Census 2021** | **CPRD Gold** | **Census 2021** |
| **United Kingdom** | <0.01% | 0.43% | 0.46% | 46.53% | 0.02% | 1.59% | 0.01% | 0.41% | 0.08% | 5.97% | 0.01% | 0.80% | 0.01% | 0.58% | 99.10% | 43.67% |

**Table 4.4.3.** Proportion of the CPRD Aurum population with a given religion compared to the proportion of the UK Census population with a given religion for 2011 and 2021.

No religion/Unknown was the default. Due to small numbers, description of religion was only undertaken at the largest geography for each database.

| **Geography** | **Buddhist** | | **Christian** | | **Hindu** | | **Jewish** | | **Muslim** | | **Sikh** | | **Other religion** | | **No religion/ Unknown** | |
| --- | --- | --- | --- | --- | --- | --- | --- | --- | --- | --- | --- | --- | --- | --- | --- | --- |
|  | **CPRD Aurum** | **Census 2011** | **CPRD Aurum** | **Census 2011** | **CPRD Aurum** | **Census 2011** | **CPRD Aurum** | **Census 2011** | **CPRD Aurum** | **Census 2011** | **CPRD Aurum** | **Census 2011** | **CPRD Aurum** | **Census 2011** | **CPRD Aurum** | **Census 2011** |
| **ENI** | 0.04% | 0.44% | 2.53% | 60.14% | 0.22% | 1.32% | 0.06% | 0.48% | 0.65% | 4.85% | 0.09% | 0.77% | 0.07% | 0.43% | 96.34% | 31.42% |
|  |  |  |  |  |  |  |  |  |  |  |  |  |  |  |  |  |
|  | **CPRD Aurum** | **Census 2021** | **CPRD Aurum** | **Census 2021** | **CPRD Aurum** | **Census 2021** | **CPRD Aurum** | **Census 2021** | **CPRD Aurum** | **Census 2021** | **CPRD Aurum** | **Census 2021** | **CPRD Aurum** | **Census 2021** | **CPRD Aurum** | **Census 2021** |
| **ENI** | 0.06% | 0.45% | 4.02% | 47.41% | 0.38% | 1.75% | 0.13% | 0.46% | 1.16% | 6.53% | 0.13% | 0.89% | 0.12% | 0.58% | 93.99% | 41.92% |

# Sexual orientation

## Sexual orientation definitions in CPRD and the Censuses

In accordance with Census information related to sexual orientation, sexual orientation was defined for all eligible patients aged ≥16 years old where possible.

Sexual Orientation variable

A categorical variable indicating:

1. = Unknown
2. = Heterosexual
3. = Homosexual
4. = Bisexual
5. = Other

The default orientation was defined as unknown or heterosexual, depending on the model.

Heterosexual was defined as having a code indicating an individual is sexually attracted to, or has a sexual partner of, the opposite sex such as heterosexual or straight. When heterosexual was defined as the default orientation, its definition also included the absence of any code of interest or having an ambiguous code, such as having a male or female sexual partner, which may later be used alongside further data to determine one of the above sexual orientations.

Homosexual was defined as having a code indicating an individual is sexually attracted to, or has a sexual partner of, the same sex such as homosexual, gay, or lesbian.

Bisexual was defined as having a code indicating an individual is sexually attracted to, or has/had a sexual partner of, the opposite and same sex, such as bisexual or sexually attracted to either sex.

Other was defined as having a code indicating an individual does not meet the definitions above, but whose sexuality is not ambiguous, such as asexual or other sexual orientation not listed.

Unknown was defined as the absence of any code of interest or having an ambiguous code, such as having a male or female sexual partner, which may later be used alongside further data to determine one of the above sexual orientations. When heterosexual was defined as the default orientation, unknown was limited to instances where an explicit “unknown sexual orientation” code was present in the record.

Sexual orientation was categorised to be comparable to the 2021 Census table for sexual orientation (17–19).

## Algorithm for determining sexual orientation in CPRD

Where one code (see 10.4) was present per patient, the patient was categorised as defined above with the exception that if this code were categorised as unknown, the patient was classified based on their sex as follows:

If patient had a code:

- indicating sexual partner/sexually attracted to male(s) and their sex is recorded as male/female, they were categorised homosexual /heterosexual.
- indicating sexual partner/sexually attracted to female(s) and their sex is recorded as female/male, they were categorised homosexual /heterosexual.

Where multiple codes were present per patient, the patient was categorised based on their most recent code’s classification, except for in the following situations:

If the patient had multiple most recent and opposing classifications, they were categorised as the first of any orientation that applies, in the order of the following list:

- Other
- Bisexual
- Heterosexual *OR* homosexual
- Other
- Unknown

Where only homosexual and heterosexual appeared together, they were categorised as unknown.

## Completeness of sexual orientation recording in CPRD

Census 2021

On the date of the 2021 Census for the UK, the proportion of the CPRD Gold population aged ≥16 years old with ANY sexual orientation coding was 0.30% (8,364 out of 2,792,324).

On the date of the 2021 Census for ENI, the proportion of the CPRD Aurum population aged ≥16 years old with ANY sexual orientation coding was 1.76% (194,076 out of 11,044,269).

On the date of the 2021 Census for UK, the proportion of the CPRD Gold and CPRD Aurum populations combined aged ≥16 years old with ANY sexual orientation coding was 1.46% (202,440 out of 13,634,153).

## Additional results for sexual orientation

**Table 5.4.1.** Proportion of the CPRD Combined, CPRD Gold, and CPRD Aurum populations with a given sexual orientation compared to the proportion of the UK Census population with a given sexual orientation for 2021.

Default categories were either Heterosexual or Unknown. Due to small numbers, description of sexual orientation was only undertaken at the largest geographies for each database. The ‘Other’ and non-default ‘Unknown’ sexual orientation categories were not represented by any codes in CPRD Gold. Sexual orientation was assessed for persons aged ≥16 years old at the time of the given Census. ‘Other’ included pansexual, asexual, queer, and all other sexual orientations. ‘Unknown’ included not answered (Census only). N/A = not applicable.

| **Geography** | **Default Category** | **Heterosexual** | | **Homosexual** | | **Bisexual** | | **Other** | | **Unknown** | |
| --- | --- | --- | --- | --- | --- | --- | --- | --- | --- | --- | --- |
|  |  | **CPRD Combined** | **Census 2021** | **CPRD Combined** | **Census 2021** | **CPRD Combined** | **Census 2021** | **CPRD Combined** | **Census 2021** | **CPRD Combined** | **Census 2021** |
| **United Kingdom** | **Heterosexual** | 99.88% | 89.39% | 0.07% | 1.53% | 0.03% | 1.27% | <0.01% | 0.33% | <0.01% | 7.48% |
|  | **Unknown** | 1.35% |  | 0.07% |  | 0.03% |  | <0.01% |  | 98.55% |  |
|  |  |  |  |  |  |  |  |  |  |  |  |
|  |  | **CPRD Gold** | **Census 2021** | **CPRD Gold** | **Census 2021** | **CPRD Gold** | **Census 2021** | **CPRD Gold** | **Census 2021** | **CPRD Gold** | **Census 2021** |
| **United Kingdom** | **Heterosexual** | 99.97% | 89.39% | 0.03% | 1.53% | 0.01% | 1.27% | N/A | 0.33% | <0.01% | 7.48% |
|  | **Unknown** | 0.27% |  | 0.03% |  | 0.01% |  |  |  | 99.70% |  |
|  |  |  |  |  |  |  |  |  |  |  |  |
|  |  | **CPRD Aurum** | **Census 2021** | **CPRD Aurum** | **Census 2021** | **CPRD Aurum** | **Census 2021** | **CPRD Aurum** | **Census 2021** | **CPRD Aurum** | **Census 2021** |
| **ENI** | **Heterosexual** | 99.86% | 89.39% | 0.08% | 1.53% | 0.04% | 1.27% | <0.01% | 0.34% | <0.01% | 7.48% |
|  | **Unknown** | 1.62% |  | 0.08% |  | 0.04% |  | <0.01% |  | 98.25% |  |

# Gender identity

## Gender identity definitions in CPRD and the Censuses

In accordance with Census information related to gender identity, gender identity characteristics were defined for all eligible patients aged ≥16 years old where possible.

Gender Identity

A categorical variable indicating:

1. = To be determined
2. = Same sex as birth
3. = Transgender
4. = Other or unknown

The default gender identity was defined as same sex as birth.

Same sex as birth was defined as having a code indicating an individual identifies with their sex registered at birth.

Transgender was defined as having a code indicating an individual does not identify with their sex registered at birth, or as assigned within their healthcare records, such as transgender or gender assignment surgery.

Other or unknown was defined as having a code indicating an individual identifies as a gender identity not listed above, such as non-binary, or an ambiguous code that indicates that they may not identify with their sex registered at birth, or as assigned within their healthcare records, such as gender dysphoria.

To be determined was defined as having a code that may be used to identify their gender identity by using accompanying data within their healthcare records, such as identifies as female or identifies as male.

Individuals were excluded from the above categories in certain instances, such as if there was any record of a code corresponding to variations of sex characteristics.

Gender identity was categorised to be comparable to the 2021 Census table for gender identity in England and Wales (20) and for Scotland (21). Gender identity was not collected by the 2021 Census in Northern Ireland.

## Algorithm for determining gender identity in CPRD

The following records that were assigned the categorical variable indicating ‘to be determined’ were re-categorised using their sex, as assigned within their healthcare records, as follows:

If an individual had a record (see 10.5) indicating:

- female gender/identifies as female gender and their sex is recorded as male/female, the record was categorised transgender /same sex as birth.
- Indicating male gender/identifies as male and their sex is recorded as female/male, the record was categorised transgender /same sex as birth.

Where one record was present per patient, the patient was categorised as previously defined.

Where multiple records were present per patient and these were all categorised the same, the patient was categorised as previously defined.

Where multiple records were present per patient, but these were not all categorised the same, the most definitive record, which we classified as either same sex as birth or transgender, were prioritised. The most recent code recorded was then assigned, unless there were multiple different, most recent events present, in which case, the individual was characterised within the category other or unknown.

All individuals with any record (see 10.5) corresponding to the exclusion criteria were removed from the final pool of patients.

## Completeness of gender identity recording in CPRD

Census 2021

On the date of the 2021 Census for ESW, the proportion of the CPRD Gold population aged ≥16 years old with ANY gender identity coding was 0.10% (2,481 out of 2,543,061).

On the date of the 2021 Census for England, the proportion of the CPRD Aurum population aged ≥16 years old with ANY gender identity coding was 0.11% (12,226 out of 10,994,292).

On the date of the 2021 Census for ESW, the proportion of the CPRD Gold and CPRD Aurum populations combined aged ≥16 years old with ANY gender identity characteristic coding was 0.11% (14,707 out of 13,537,353).

# Care home residence

## Care home residence definition in CPRD and the Censuses

In accordance with Census information related to care home residence, care home residence characteristics were defined for all eligible patients where possible.

Care Home Residence

A subset of residence in a medical or care establishment.

A binary variable detailing:

1. = Does not reside in a care home/Unknown
2. = Resides in a care home.

The default status was defined as not residing in a care home.

Residence in a care home was defined as residing in a care home with or without nursing, run by the local authority or another authority.

Care home residence was categorised to be comparable to the Care Home subsets of the 2011 and 2021 Census tables for Communal Housing (22–25).

## Algorithm for determining care home residence in CPRD

All events stemming from the code list (see 10.6) for care home residence were identified.

In the first instance, the most recent code prior to the given UK Census date pertaining to care home residence was taken to define care home residence for a given patient.

If a patient had more than one most recent code pertaining to care home residence the following steps were taken to select a single code to represent the characteristic:

- For care home residence, codes indicating residing in a care home were prioritised over codes indicating not residing in a care home.

## Completeness of care home residence recording in CPRD

Census 2011

On the date of the 2011 Census for the UK, the proportion of the CPRD Gold population with ANY care home residence coding was 0.26% (18,275 out of 6,936,975).

On the date of the 2011 Census for ENI, the proportion of the CPRD Aurum population with ANY care home residence coding was 0.22% (27,561 out of 12,109,773).

On the date of the 2011 Census for UK, the proportion of the CPRD Gold and CPRD Aurum populations combined with ANY care home residence coding was 0.25% (42,401 out of 17,232,402).

Census 2021

On the date of the 2021 Census for the UK, the proportion of the CPRD Gold population with ANY care home residence coding was 0.28% (9,436 out of 3,368,293).

On the date of the 2021 Census for ENI, the proportion of the CPRD Aurum population with ANY care home residence coding was 0.47% (63,254 out of 13,578,103).

On the date of the 2021 Census for UK, the proportion of the CPRD Gold and CPRD Aurum populations combined with ANY care home residence coding was 0.43% (72,687 out of 16,935,144).

# Unpaid carer responsibilities

## Unpaid carer responsibilities definition in CPRD and the Censuses

In accordance with Census information related to unpaid care, unpaid care was defined for all eligible patients ≥5 years old where possible.

In CPRD, each medical code was assigned a flag:

- 1 = care is explicitly unpaid
- 2 = it is unclear if care is unpaid (ambiguous)

Each code was assigned a flag of 2 by default, unless the term indicates that it is unpaid. For example:

- Codes starting with “carer of (a) person” were considered to be explicitly unpaid because care was provided to a single person.
- Both the Carer strain index score and Carer Support Needs Assessment Tool are designed for informal carers.
- It was unclear whether Carer (annual) health checks are only for paid carers, thus these codes were classified as 2.

Unpaid care definitions in CPRD were compared with the unpaid care data collected in the UK 2011 and 2021 Censuses (26–29). The Census data were collapsed into two categories: “Does not provide unpaid care” and “Does provide at least 1 hour of unpaid care per week”.

## Algorithm for determining unpaid carer responsibilities in CPRD

All events stemming from the code list (see 10.7) for unpaid carer responsibilities were identified.

The most recent code prior to the given UK Census date pertaining to unpaid carer responsibilities was taken to define the carer status of a given patient.

Patients without a code specifying unpaid carer responsibilities were assumed to not provide unpaid care.

## Completeness of unpaid carer responsibilities recording in CPRD

Census 2011

On the date of the 2011 Census for the UK, the proportion of the CPRD Gold population aged ≥5 years old with ANY codes related to unpaid care:

- Was 0.62% (39,827 out of 6,455,576) based on the broad criteria.
- Was 0.04% (2,541 out of 6,455,576) based on the strict criteria.

On the date of the 2011 Census for ENI, the proportion of the CPRD Aurum population aged ≥5 years old with ANY codes related to unpaid care:

- Was 0.63% (70,800 out of 11,327,861) based on the broad criteria.
- Was 0.02% (2,774 out of 11,327,861) based on the strict criteria.

On the date of the 2011 Census for UK, the proportion of the CPRD Gold and CPRD Aurum populations combined aged ≥5 years old with ANY codes related to unpaid care:

- Was 0.61% (98,189 out of 16,141,186) based on the broad criteria.
- Was 0.03% (4,937 out of 16,141,186) based on the strict criteria.

Census 2021

On the date of the 2021 Census for the UK, the proportion of the CPRD Gold population aged ≥5 years old (≥3 in Scotland) with ANY codes related to unpaid care:

- Was 1.51% (48,892 out of 3,237,060) based on the broad criteria.
- Was 0.08% (2,678 out of 3,237,060) based on the strict criteria.

On the date of the 2021 Census for ENI, the proportion of the CPRD Aurum population aged ≥5 years old with ANY codes related to unpaid care:

- Was 1.34% (172,089 out of 12,858,795) based on the broad criteria.
- Was 0.13% (17,087 out of 12,858,795) based on the strict criteria.

On the date of the 2021 Census for UK, the proportion of the CPRD Gold and CPRD Aurum populations combined aged ≥5 years old (≥3 in Scotland) with ANY codes related to unpaid care:

- Was 1.35% (217,601 out of 16,091,384) based on the broad criteria.
- Was 0.12% (19,692 out of 16,091,384) based on the strict criteria.

# Relationship status

## Relationship status definitions in CPRD and the Censuses

In accordance with Census information related to legal partnership status, relationship status and legal partnership status were defined for all eligible patients ≥16 years old where possible.

The following relationship status characteristics were defined:

1. Legal partnership status
2. Relationship status

Legal partnership status

A categorical variable detailing:

0 = Never married/Not in a registered civil partnership

1 = Married/In a registered civil partnership

2 = Separated but still legally married or in a registered civil partnership

3 = Divorced/Dissolved civil partnership

4 = Widowed/Surviving Civil Partner

5 = Unknown/not recorded

Being never married/not in a registered civil partnership was defined as having a code indicating no partner at present, single, relationship breakdown, separated from cohabitee etc.

Being married/in a registered civil partnership was defined as having a code indicating having a husband/wife/spouse or something related to marital/marriage/married etc.

Being separated but still legally married or in a registered civil partnership was defined as having a code indicating husband/wife/spouse left home, marital breakdown, separated etc.

Being divorced/dissolved civil partnership was defined as having a code indicating divorce.

Being widowed/surviving civil partner was defined as having a code indicating death of wife/husband/spouse, widow, deceased wife/husband/spouse etc.

Not having recorded history of a legal partnership status was coded as unknown.

Legal partnership status was categorised to be comparable to the 2011 and 2021 Census tables for marital status (30–32).

Relationship status

A categorical variable detailing:

0 = Not in a relationship (no history of legally recognised partnership)

1 = Married/In a registered civil partnership

2 = Separated but still legally married or in a registered civil partnership (not in new relationship)

3 = Divorced/Dissolved civil partnership (not in new relationship, legally recognised or not)

4 = Widowed/Surviving Civil Partner (not in new relationship, legally recognised or not)

5 = In a relationship (not legally recognised, may have a history of legally recognised partnership)

6 = Unknown

Being not in a relationship was defined as having a code indicating no partner at present, single, relationship breakdown, separated from cohabitee etc. & not having recorded history of a legal partnership status.

Being married/in a registered civil partnership was defined as having a code indicating having a husband/wife/spouse or something related to marital/marriage/married etc.

Being separated but still legally married or in a registered civil partnership was defined as having a code indicating husband/wife/spouse left home, marital breakdown, separated etc. & not having a code indicating being in a relationship.

Being divorced/dissolved civil partnership was defined as having a code indicating divorce & not having a code indicating being in a relationship.

Being widowed/surviving civil partner was defined as having a code indicating death of wife/husband/spouse, widow, deceased wife/husband/spouse etc. & not having a code indicating being in a relationship.

Being in a relationship was defined as having a code indicating having a partner/boyfriend/girlfriend, relationship, cohabitee etc.

Being unknown was defined as either having no recorded code or having a code indicating marital status unknown/not disclosed etc.

Relationship status is not comparable to any Census data tables.

## Algorithm for determining relationship status in CPRD

For CPRD Gold, a marital status variable already exists. We considered the CPRD Gold marital status record to be the oldest recorded marital status for each patient, working on the assumption that this was probably recorded at registration and not changed since.

Marital status records with no corresponding date were considered to be the oldest recorded marital status for that patient.

In all algorithms, patients with more than one recorded marital status in their records were categorised based on the most recent recorded marital status, except for in the following situations:

Legal partnership status algorithm

- Most recent status unknown: Second most recent status
- Second most recent status unknown: Unknown
- Most recent status single but previous most recent status divorced/widowed/separated: Divorced/widowed/separated
- Most recent status single but previous most recent status married: Unknown
- Most recent status single (code indicates partner died) & previous most recent status married: Widowed
- Most recent status single & previous married status (code indicates marital problems) & previous divorced/separated status: Divorced/separated
- Patients with multiple most recent recorded marital statuses were categorised as follows:
  - Single & married: Unknown
  - Single & divorced/separated/widowed: divorced/separated/widowed (if more than one, categorise as unknown)
  - Married (code indicates marital problems) & divorced/separated: Divorced/separated
  - Married (code refers to spouse being sick) & widowed: Widowed
  - Divorced & separated: Divorced

Relationship status

The algorithm followed the same rules as above with the addition of:

- Most recent status relationship & previous most recent married: Married
- Patients with multiple most recent recorded marital statuses were categorised as follows:
  - Relationship & married: Married
  - Divorced/separated/widowed & relationship: Relationship
- Patients with more complex marital status histories that did not fit into any of these situations were categorised as unknown.

## Completeness of relationship status recording in CPRD

Census 2011

On the date of the 2011 census for the UK, the proportion of the CPRD Gold population aged ≥16 years old with ANY marital status characteristic coding:

- For legal partnership status was 30.77% (1,576,063 out of 5,122,629).
- For relationship status was 70.78% (3,625,777 out of 5,122,629).

On the date of the 2011 census for ENI, the proportion of the CPRD Aurum population aged ≥16 years old with ANY marital status characteristic coding:

- For legal partnership status was 10.24% (1,240,425 out of 12,109,773).
- For relationship status was 14.92% (1,806,763 out of 12,109,773).

On the date of the 2011 census for the UK, the proportion of the CPRD Gold and CPRD Aurum populations combined aged ≥16 years old with ANY marital status characteristic coding:

- For legal partnership status was 16.34% (2,816,488 out of 17,232,402).
- For relationship status was 31.53% (5,432,540 out of 17,232,402).

Census 2021

On the date of the 2021 census for the UK, the proportion of the CPRD Gold population aged ≥16 years old with ANY marital status characteristic coding:

- For legal partnership status was 34.06% (945,213 out of 2,775,001).
- For relationship status was 65.79% (1,825,747 out of 2,775,001).

On the date of the 2021 census for the ENI, the proportion of the CPRD Aurum population aged ≥16 years old with ANY marital status characteristic coding:

- For legal partnership status was 23.21% (2,562,871 out of 11,044,269).
- For relationship status was 43.16% (4,766,804 out of 11,044,269).

On the date of the 2021 census for the UK, the proportion of the CPRD Gold and CPRD Aurum populations combined aged ≥16 years old with ANY marital status characteristic coding:

- For legal partnership status was 25.39% (3,520,346 out of 13,819,270).
- For relationship status was 47.71% (6,592,551 out of 13,819,270).

## Additional results for relationship status

**Table 9.4.1.** Proportion of the CPRD population with a given legal partnership status in each country by legal partnership status compared to the proportion of the UK Census population in each country by legal partnership status for 2011 and 2021. Legal partnership status was assessed for persons aged ≥16 years old at the time of the given Census. Please see the main manuscript for the results of CPRD Combined. Never married/not in a registered civil partnership was the default.

| **Geography** | **Never Married/Not in a Registered Civil Partnership** | | | **Married/In a Registered Civil Partnership** | | | **Separated but still Legally Married/In a Registered Civil Partnership** | | | **Divorced/Dissolved Civil Partnership** | | | **Widowed/Surviving Civil Partner** | | |
| --- | --- | --- | --- | --- | --- | --- | --- | --- | --- | --- | --- | --- | --- | --- | --- |
|  | **CPRD Gold** | **CPRD Aurum** | **Census 2011** | **CPRD Gold** | **CPRD Aurum** | **Census 2011** | **CPRD Gold** | **CPRD Aurum** | **Census 2011** | **CPRD Gold** | **CPRD Aurum** | **Census 2011** | **CPRD Gold** | **CPRD Aurum** | **Census 2011** |
| **United Kingdom** | 46.36% |  | 34.69% | 45.20% |  | 46.72% | 1.79% |  | 2.72% | 2.73% |  | 8.84% | 3.93% |  | 7.03% |
| **England** | 34.21% | 36.47% | 34.64% | 54.55% | 45.12% | 46.82% | 2.36% | 3.62% | 2.65% | 3.86% | 4.49% | 8.97% | 5.02% | 10.30% | 6.91% |
| **Northern Ireland** | 47.47% | 50.41% | 36.14% | 46.28% | 36.91% | 47.65% | 1.59% | 4.87% | 3.98% | 1.35% | 1.94% | 5.45% | 3.31% | 5.87% | 6.78% |
| **Scotland** | 56.12% |  | 35.38% | 37.53% |  | 45.41% | 1.45% |  | 3.22% | 1.87% |  | 8.21% | 3.03% |  | 7.77% |
| **Wales** | 38.83% |  | 33.52% | 50.85% |  | 46.74% | 1.73% |  | 2.18% | 3.75% |  | 9.66% | 4.84% |  | 7.90% |
|  |  |  |  |  |  |  |  |  |  |  |  |  |  |  |  |
|  | **CPRD Gold** | **CPRD Aurum** | **Census 2021** | **CPRD Gold** | **CPRD Aurum** | **Census 2021** | **CPRD Gold** | **CPRD Aurum** | **Census 2021** | **CPRD Gold** | **CPRD Aurum** | **Census 2021** | **CPRD Gold** | **CPRD Aurum** | **Census 2021** |
| **United Kingdom** | 46.84% | 40.55% | 37.92% | 45.56% | 47.64% | 44.62% | 1.83% | 2.50% | 2.30% | 2.68% | 3.99% | 8.96% | 3.09% | 5.31% | 6.20% |
| **England** | 35.19% | 40.54% | 37.89% | 55.26% | 47.64% | 44.65% | 2.38% | 2.50% | 2.24% | 3.58% | 4.00% | 9.11% | 3.59% | 5.32% | 6.12% |
| **Northern Ireland** | 45.09% | 42.94% | 38.07% | 48.12% | 48.24% | 45.77% | 1.88% | 3.35% | 3.78% | 1.76% | 1.84% | 6.02% | 3.14% | 3.63% | 6.36% |
| **Scotland** | 50.71% |  | 38.13% | 42.33% |  | 44.00% | 1.74% |  | 2.45% | 2.33% |  | 8.38% | 2.90% |  | 7.04% |
| **Wales** | 39.12% |  | 37.18% | 51.54% |  | 43.82% | 1.91% |  | 2.05% | 3.93% |  | 9.87% | 3.50% |  | 7.08% |

**Table 9.2.** Proportion of the CPRD Combined population with a given relationship status, legally recognised or not, in each country in 2011 and 2021. Unknown relationship status was the default.

| **Geography** | **Not in a Relationship (no history of legally recognised partnership)** | **Married/In a Registered Civil Partnership** | **Separated but still Legally Married/In a Registered Civil Partnership (not in a new relationship)** | **Divorced/Dissolved Civil Partnership (not in a new relationship)** | **Widowed/Surviving Civil Partner (not in a new relationship)** | **In a Relationship (not legally recognised, may have history of legally recognised partnership)** | **Unknown** |
| --- | --- | --- | --- | --- | --- | --- | --- |
| **2011** | | | | | | | |
| **United Kingdom** | 14.14% | 13.86% | 0.54% | 0.82% | 1.20% | 0.92% | 68.52% |
| **England** | 4.27% | 5.77% | 0.38% | 0.51% | 1.06% | 1.07% | 86.95% |
| **Northern Ireland** | 12.01% | 11.58% | 0.44% | 0.35% | 0.87% | 0.33% | 74.41% |
| **Scotland** | 29.14% | 19.53% | 0.74% | 0.97% | 1.58% | 0.54% | 47.50% |
| **Wales** | 7.90% | 10.40% | 0.35% | 0.75% | 0.99% | 0.91% | 78.70% |
| **2021** | | | | | | | |
| **United Kingdom** | 10.51% | 11.94% | 0.57% | 0.90% | 1.19% | 2.02% | 72.87% |
| **England** | 9.11% | 11.05% | 0.56% | 0.90% | 1.21% | 2.19% | 74.97% |
| **Northern Ireland** | 13.75% | 14.89% | 0.63% | 0.54% | 0.99% | 0.68% | 68.53% |
| **Scotland** | 23.00% | 19.29% | 0.78% | 1.05% | 1.32% | 1.25% | 53.30% |
| **Wales** | 8.33% | 11.06% | 0.40% | 0.83% | 0.75% | 1.37% | 77.26% |

# Code lists

## Code list for determining country of birth in CPRD

The CPRD Code Browser was used to generate a list of codes, both in CPRD Gold and CPRD Aurum. The search strategy included terms such as “*born*in*”, “*migrant*”, “*country*birth*”, etc.

All codes that are related to country of birth were considered. The final code lists had 240 codes for CPRD Gold and 285 codes for CPRD Aurum.

| Medcode | Description | Country of birth category | Source |
| --- | --- | --- | --- |
| 9292 | immigrant | Migrant or unknown | Gold |
| 47073 | family reunion immigrant | Migrant or unknown | Gold |
| 8892 | born in - country | Migrant or unknown | Gold |
| 23398 | social migrant | Migrant or unknown | Gold |
| 44407 | illegal migrant | Migrant or unknown | Gold |
| 4114 | immigrant | Migrant or unknown | Gold |
| 101846 | country of birth unknown | Migrant or unknown | Gold |
| 30800 | country of birth (European) | Europe | Gold |
| 32055 | born in Albania | Europe | Gold |
| 93935 | born in Andorra | Europe | Gold |
| 32313 | born in Austria | Europe | Gold |
| 41337 | born in Azerbaijan | Europe | Gold |
| 32352 | born in Belgium | Europe | Gold |
| 41233 | born in Belorussia | Europe | Gold |
| 32397 | born in Bosnia - Herzegovina | Europe | Gold |
| 32169 | born in Bulgaria | Europe | Gold |
| 41210 | born in Croatia | Europe | Gold |
| 32190 | born in Cyprus | Europe | Gold |
| 26463 | born in Czech Republic | Europe | Gold |
| 32186 | born in Denmark | Europe | Gold |
| 32139 | born in Estonia | Europe | Gold |
| 32173 | born in Finland | Europe | Gold |
| 32081 | born in France | Europe | Gold |
| 32085 | born in Germany | Europe | Gold |
| 32080 | born in Greece | Europe | Gold |
| 32688 | born in Hungary | Europe | Gold |
| 41312 | born in Iceland | Europe | Gold |
| 32113 | born in Ireland | Europe | Gold |
| 32116 | born in Italy | Europe | Gold |
| 25730 | born in Kosovo | Europe | Gold |
| 32242 | born in Latvia | Europe | Gold |
| 95708 | born in Liechtenstein | Europe | Gold |
| 28301 | born in Lithuania | Europe | Gold |
| 69131 | born in Luxembourg | Europe | Gold |
| 41364 | born in Malta | Europe | Gold |
| 71190 | born in Monaco | Europe | Gold |
| 41213 | born in Norway | Europe | Gold |
| 32094 | born in Poland | Europe | Gold |
| 32160 | born in Portugal | Europe | Gold |
| 41357 | born in Republic of Ireland | Europe | Gold |
| 32345 | born in Romania | Europe | Gold |
| 98530 | born in San Marino | Europe | Gold |
| 32119 | born in Slovakia | Europe | Gold |
| 41304 | born in Slovenia | Europe | Gold |
| 32097 | born in Spain | Europe | Gold |
| 32098 | born in Sweden | Europe | Gold |
| 32807 | born in Switzerland | Europe | Gold |
| 32150 | born in the Netherlands | Europe | Gold |
| 32103 | born in Ukraine | Europe | Gold |
| 99119 | born in Vatican City | Europe | Gold |
| 25256 | born in Yugoslavia | Europe | Gold |
| 96824 | born in former Yugoslav Republic of Macedonia | Europe | Gold |
| 100517 | born in Serbia | Europe | Gold |
| 109457 | born in Montenegro | Europe | Gold |
| 109898 | born in Belarus | Europe | Gold |
| 109260 | born in Republic of Moldova | Europe | Gold |
| 109093 | born in guernsey | Europe | Gold |
| 109092 | born in jersey | Europe | Gold |
| 112634 | born in isle of man | Europe | Gold |
| 115587 | born in Faroe islands | Europe | Gold |
| 111774 | born in Aland islands | Europe | Gold |
| 11552 | country of birth (Asian) | Asia | Gold |
| 32060 | born in Afghanistan | Asia | Gold |
| 32361 | born in Armenia | Asia | Gold |
| 32273 | born in Bahrain | Asia | Gold |
| 32125 | born in Bangladesh | Asia | Gold |
| 97390 | born in Bhutan | Asia | Gold |
| 62298 | born in Brunei | Asia | Gold |
| 32254 | born in Burma | Asia | Gold |
| 32202 | born in Chechnya | Asia | Gold |
| 32058 | born in China | Asia | Gold |
| 41354 | born in Democratic People's Republic of Korea | Asia | Gold |
| 57186 | born in East Timor | Asia | Gold |
| 49402 | born in Georgia | Europe | Gold |
| 32220 | born in Hong Kong | Asia | Gold |
| 32082 | born in India | Asia | Gold |
| 41289 | born in Indonesia | Asia | Gold |
| 26426 | born in Iran | Asia | Gold |
| 12713 | born in Iraq | Asia | Gold |
| 25007 | born in Israel | Asia | Gold |
| 32070 | born in Japan | Asia | Gold |
| 41311 | born in Jordan | Asia | Gold |
| 41292 | born in Kazakhstan | Asia | Gold |
| 41217 | born in Kuwait | Asia | Gold |
| 32128 | born in Kyrgyzstan | Asia | Gold |
| 69431 | born in Laos | Asia | Gold |
| 41211 | born in Lebanon | Asia | Gold |
| 32171 | born in Malaysia | Asia | Gold |
| 91328 | born in Maldives | Asia | Gold |
| 65310 | born in mail | Asia | Gold |
| 41351 | born in Mongolia | Asia | Gold |
| 32217 | born in Nepal | Asia | Gold |
| 66560 | born in North Korea | Asia | Gold |
| 51778 | born in Oman | Asia | Gold |
| 25995 | born in Pakistan | Asia | Gold |
| 41297 | born in Palestine | Asia | Gold |
| 25092 | born in Philippines | Asia | Gold |
| 42639 | born in Qatar | Asia | Gold |
| 41356 | born in Republic of Korea | Asia | Gold |
| 32127 | born in Russia | Europe | Gold |
| 32144 | born in Saudi Arabia | Asia | Gold |
| 32157 | born in Singapore | Asia | Gold |
| 41228 | born in South Korea | Asia | Gold |
| 32245 | born in Sri Lanka | Asia | Gold |
| 28529 | born in Syria | Asia | Gold |
| 32390 | born in Taiwan | Asia | Gold |
| 100007 | born in Tajikistan | Asia | Gold |
| 32135 | born in Thailand | Asia | Gold |
| 32114 | born in turkey | Europe | Gold |
| 41302 | born in Turkmenistan | Asia | Gold |
| 41372 | born in United Arab Emirates | Asia | Gold |
| 41402 | born in Uzbekistan | Asia | Gold |
| 26334 | born in Vietnam | Asia | Gold |
| 41344 | born in Yemen | Asia | Gold |
| 109226 | born in Christmas Island | Asia | Gold |
| 114221 | born in Cocos (Keeling) Islands | Asia | Gold |
| 32053 | country of birth (American) | Americas | Gold |
| 32325 | born in Argentina | Americas | Gold |
| 94050 | born in Belize | Americas | Gold |
| 41230 | born in Bolivia | Americas | Gold |
| 32075 | born in Brazil | Americas | Gold |
| 32342 | born in British Guyana | Americas | Gold |
| 30606 | born in Canada | Americas | Gold |
| 41291 | born in Chile | Americas | Gold |
| 32303 | born in Columbia | Americas | Gold |
| 64984 | born in Costa Rica | Americas | Gold |
| 32311 | born in Ecuador | Americas | Gold |
| 63943 | born in El Salvador | Americas | Gold |
| 32120 | born in Grenada | Americas | Gold |
| 69806 | born in Guatemala | Americas | Gold |
| 38075 | born in Guyana | Americas | Gold |
| 41280 | born in Honduras | Americas | Gold |
| 32201 | born in Mexico | Americas | Gold |
| 74892 | born in Nicaragua | Americas | Gold |
| 63923 | born in Panama | Americas | Gold |
| 41399 | born in Paraguay | Americas | Gold |
| 32166 | born in Peru | Americas | Gold |
| 69426 | born in Suriname | Americas | Gold |
| 32072 | born in USA | Americas | Gold |
| 41367 | born in Uruguay | Americas | Gold |
| 32090 | born in Venezuela | Americas | Gold |
| 32102 | country of birth (African) | Africa | Gold |
| 32079 | born in Algeria | Africa | Gold |
| 32260 | born in Angola | Africa | Gold |
| 41318 | born in Benin | Africa | Gold |
| 41316 | born in Botswana | Africa | Gold |
| 58527 | born in Burkina Faso | Africa | Gold |
| 32167 | born in Burundi | Africa | Gold |
| 32255 | born in Cameroon | Africa | Gold |
| 69560 | born in Cape Verde Islands | Africa | Gold |
| 99258 | born in Central African Republic | Africa | Gold |
| 38117 | born in Chad | Africa | Gold |
| 101158 | born in Comoros Islands | Africa | Gold |
| 25664 | born in Congo | Africa | Gold |
| 68866 | born in Djibouti | Africa | Gold |
| 32333 | born in Egypt | Africa | Gold |
| 93923 | born in Equatorial Guinea | Africa | Gold |
| 32158 | born in Ethiopia | Africa | Gold |
| 59657 | born in Gabon | Africa | Gold |
| 32207 | born in Gambia | Africa | Gold |
| 32067 | born in Ghana | Africa | Gold |
| 41365 | born in Guinea Bissau | Africa | Gold |
| 32347 | born in Guinea Republic | Africa | Gold |
| 32099 | born in Ivory Coast | Africa | Gold |
| 32112 | born in Kenya | Africa | Gold |
| 93697 | born in Lesotho | Africa | Gold |
| 32237 | born in Liberia | Africa | Gold |
| 41341 | born in Libya | Africa | Gold |
| 37197 | born in Madagascar | Africa | Gold |
| 32115 | born in Malawi | Africa | Gold |
| 69143 | born in Mauritania | Africa | Gold |
| 32140 | born in Mauritius | Africa | Gold |
| 32108 | born in Morocco | Africa | Gold |
| 32162 | born in Mozambique | Africa | Gold |
| 32233 | born in Namibia | Africa | Gold |
| 30224 | born in Niger | Africa | Gold |
| 32111 | born in Nigeria | Africa | Gold |
| 32331 | born in Rwanda | Africa | Gold |
| 101591 | born in Sao Tome and Principe | Africa | Gold |
| 32309 | born in Senegal | Africa | Gold |
| 32197 | born in Sierra Leone | Africa | Gold |
| 32189 | born in Somalia | Africa | Gold |
| 12458 | born in South Africa | Africa | Gold |
| 36794 | born in Sudan | Africa | Gold |
| 41350 | born in Swaziland | Africa | Gold |
| 25133 | born in Tanzania | Africa | Gold |
| 63927 | born in The Gambia | Africa | Gold |
| 32741 | born in Tunisia | Africa | Gold |
| 32068 | born in Uganda | Africa | Gold |
| 41209 | born in Zaire | Africa | Gold |
| 32168 | born in Zambia | Africa | Gold |
| 25008 | born in Zimbabwe | Africa | Gold |
| 64949 | born in Eritrea | Africa | Gold |
| 109992 | born in Democratic Republic of Congo | Africa | Gold |
| 41327 | country of birth (Australasian) | Australasia | Gold |
| 32061 | born in Australia | Australasia | Gold |
| 32074 | born in New Zealand | Australasia | Gold |
| 49907 | country of birth (Atlantic) | Americas | Gold |
| 32301 | born in Antigua and Barbuda | Americas | Gold |
| 58533 | born in Bahamas | Americas | Gold |
| 32089 | born in Barbados | Americas | Gold |
| 41290 | born in Cuba | Americas | Gold |
| 32369 | born in Dominican Republic | Americas | Gold |
| 58192 | born in Haiti | Americas | Gold |
| 32076 | born in Jamaica | Americas | Gold |
| 69135 | born in Puerto Rico | Americas | Gold |
| 66553 | born in St. Kitts and Nevis | Americas | Gold |
| 32131 | born in St. Lucia | Americas | Gold |
| 66551 | born in St. Vincent | Americas | Gold |
| 32304 | born in Togo | Africa | Gold |
| 32062 | born in Trinidad and Tobago | Americas | Gold |
| 99431 | born in Dominica | Americas | Gold |
| 108271 | born in Aruba | Americas | Gold |
| 113466 | born in United States Virgin Islands | Americas | Gold |
| 109458 | born in Saint Vincent and the Grenadines | Americas | Gold |
| 111700 | born in Sint Maarten | Americas | Gold |
| 32417 | country of birth (Pacific) | Australasia | Gold |
| 42635 | born in Fiji | Australasia | Gold |
| 115637 | born in Nauru | Australasia | Gold |
| 64120 | born in Papua New Guinea | Australasia | Gold |
| 32293 | born in Seychelles | Africa | Gold |
| 48297 | born in Solomon Islands | Australasia | Gold |
| 47559 | born in Tonga | Australasia | Gold |
| 57189 | born in Tuvalu | Australasia | Gold |
| 111392 | born in Vanuatu | Australasia | Gold |
| 103965 | born in Western Samoa | Australasia | Gold |
| 109791 | born in American Samoa | Australasia | Gold |
| 96295 | born in British overseas territory | Migrant or unknown | Gold |
| 105923 | born in Montserrat | Americas | Gold |
| 98038 | born in Bermuda | Americas | Gold |
| 104983 | born in Anguilla | Americas | Gold |
| 109727 | born in British Virgin Islands | Americas | Gold |
| 116259 | born in Turks and Caicos Islands | Americas | Gold |
| 109276 | born in Saint Helena, Ascension and Tristan da Cunha | Americas | Gold |
| 115970 | born in British Indian Ocean Territory | Asia | Gold |
| 112269 | born in Cayman Islands | Americas | Gold |
| 103364 | born in Martinique | Americas | Gold |
| 111051 | born in French Guiana | Americas | Gold |
| 108936 | born in Guadeloupe | Americas | Gold |
| 93643 | [v]social migrant | Migrant or unknown | Gold |
| 1917571000006115 | born in Isle of Man | Europe | Aurum |
| 337696018 | migrant | Migrant or unknown | Aurum |
| 1675501000006110 | born in Gibraltar | Europe | Aurum |
| 2003011000006118 | born in South Sudan | Africa | Aurum |
| 1724361000006114 | born in Serbia | Europe | Aurum |
| 1656501000006115 | born in Montserrat | Americas | Aurum |
| 1711231000006117 | country of birth unknown | Migrant or unknown | Aurum |
| 14505071000006116 | born in Colombia | Americas | Aurum |
| 14726951000006111 | born in Cabo Verde | Africa | Aurum |
| 910461000006113 | [rfc] immigrant - new arrival | Migrant or unknown | Aurum |
| 8330481000006114 | born in Réunion | Africa | Aurum |
| 993961000006112 | born in Eritrea | Africa | Aurum |
| 6057281000006115 | country of birth | Migrant or unknown | Aurum |
| 1662431000006117 | born in Martinique | Americas | Aurum |
| 251268019 | immigrant | Migrant or unknown | Aurum |
| 2549144013 | family reunion immigrant | Migrant or unknown | Aurum |
| 459868012 | born in - country | Migrant or unknown | Aurum |
| 250504013 | social migrant | Migrant or unknown | Aurum |
| 250507018 | illegal migrant | Migrant or unknown | Aurum |
| 785351000006117 | immigrant | Migrant or unknown | Aurum |
| 1166121000000117 | country of birth unknown | Migrant or unknown | Aurum |
| 459869016 | country of birth (European) | Europe | Aurum |
| 459880016 | born in Albania | Europe | Aurum |
| 459882012 | born in Andorra | Europe | Aurum |
| 459890012 | born in Austria | Europe | Aurum |
| 459891011 | born in Azerbaijan | Europe | Aurum |
| 459896018 | born in Belgium | Europe | Aurum |
| 459898017 | born in Belorussia | Europe | Aurum |
| 459902011 | born in Bosnia - herzegovnia | Europe | Aurum |
| 459907017 | born in Bulgaria | Europe | Aurum |
| 459924011 | born in Croatia | Europe | Aurum |
| 459926013 | born in Cyprus | Europe | Aurum |
| 459927016 | born in Czech republic | Europe | Aurum |
| 459929018 | born in Denmark | Europe | Aurum |
| 459938016 | born in Estonia | Europe | Aurum |
| 459941013 | born in Finland | Europe | Aurum |
| 459942018 | born in France | Europe | Aurum |
| 459946015 | born in Germany | Europe | Aurum |
| 459948019 | born in Greece | Europe | Aurum |
| 459957013 | born in Hungary | Europe | Aurum |
| 459958015 | born in Iceland | Europe | Aurum |
| 459963016 | born in Ireland | Europe | Aurum |
| 459965011 | born in Italy | Europe | Aurum |
| 459973019 | born in Kosovo | Europe | Aurum |
| 459977018 | born in Latvia | Europe | Aurum |
| 459982013 | born in Liechtenstein | Europe | Aurum |
| 459983015 | born in Lithuania | Europe | Aurum |
| 459984014 | born in Luxembourg | Europe | Aurum |
| 459993010 | born in Malta | Europe | Aurum |
| 459998018 | born in Monaco | Europe | Aurum |
| 460011017 | born in Norway | Europe | Aurum |
| 460020014 | born in Poland | Europe | Aurum |
| 460021013 | born in Portugal | Europe | Aurum |
| 460024017 | born in Republic of Ireland | Europe | Aurum |
| 460026015 | born in Romania | Europe | Aurum |
| 460029010 | born in San Marino | Europe | Aurum |
| 460037019 | born in Slovakia | Europe | Aurum |
| 460038012 | born in Slovenia | Europe | Aurum |
| 460043017 | born in Spain | Europe | Aurum |
| 460051019 | born in Sweden | Europe | Aurum |
| 460052014 | born in Switzerland | Europe | Aurum |
| 460059017 | born in the Netherlands | Europe | Aurum |
| 460069011 | born in Ukraine | Europe | Aurum |
| 460074015 | born in Vatican City | Europe | Aurum |
| 460079013 | born in Yugoslavia | Europe | Aurum |
| 636901000000118 | born in former Yugoslav Republic of Macedonia | Europe | Aurum |
| 1169001000000116 | born in Serbia | Europe | Aurum |
| 2221741000000112 | born in Montenegro | Europe | Aurum |
| 2360941000000118 | born in Belarus | Europe | Aurum |
| 2360981000000114 | born in Republic of Moldova | Europe | Aurum |
| 2361521000000113 | born in Guernsey | Europe | Aurum |
| 2361561000000117 | born in Jersey | Europe | Aurum |
| 2361601000000117 | born in Isle of Man | Europe | Aurum |
| 2362871000000117 | born in Faroe Islands | Europe | Aurum |
| 2362911000000115 | born in Greenland | Europe | Aurum |
| 2362951000000116 | born in Svalbard and Jan Mayen | Europe | Aurum |
| 2369421000000111 | born in Aland islands | Europe | Aurum |
| 459870015 | country of birth (Asian) | Asia | Aurum |
| 459879019 | born in Afghanistan | Asia | Aurum |
| 459888011 | born in Armenia | Asia | Aurum |
| 459893014 | born in Bahrain | Asia | Aurum |
| 459894015 | born in Bangladesh | Asia | Aurum |
| 459900015 | born in Bhutan | Asia | Aurum |
| 459906014 | born in Brunei | Asia | Aurum |
| 459909019 | born in Burma | Asia | Aurum |
| 459917010 | born in Chechnya | Asia | Aurum |
| 459919013 | born in China | Asia | Aurum |
| 459928014 | born in Democratic People's Republic of Korea | Asia | Aurum |
| 459932015 | born in East Timor | Asia | Aurum |
| 459945016 | born in Georgia | Europe | Aurum |
| 459956016 | born in Hong Kong | Asia | Aurum |
| 459959011 | born in India | Asia | Aurum |
| 459960018 | born in Indonesia | Asia | Aurum |
| 459961019 | born in Iran | Asia | Aurum |
| 459962014 | born in Iraq | Asia | Aurum |
| 459964010 | born in Israel | Asia | Aurum |
| 459968013 | born in Japan | Asia | Aurum |
| 459969017 | born in Jordan | Asia | Aurum |
| 459970016 | born in Kazakhstan | Asia | Aurum |
| 459974013 | born in Kuwait | Asia | Aurum |
| 459975014 | born in Kyrgyzstan | Asia | Aurum |
| 459976010 | born in Laos | Asia | Aurum |
| 459978011 | born in Lebanon | Asia | Aurum |
| 459989016 | born in Malaysia | Asia | Aurum |
| 459991012 | born in Maldives | Asia | Aurum |
| 459992017 | born in mail | Asia | Aurum |
| 459999014 | born in Mongolia | Asia | Aurum |
| 460004016 | born in Nepal | Asia | Aurum |
| 460009014 | born in North Korea | Asia | Aurum |
| 460012012 | born in Oman | Asia | Aurum |
| 460013019 | born in Pakistan | Asia | Aurum |
| 460014013 | born in Palestine | Asia | Aurum |
| 460019015 | born in Philippines | Asia | Aurum |
| 460023011 | born in Qatar | Asia | Aurum |
| 460025016 | born in Republic of Korea | Asia | Aurum |
| 460027012 | born in Russia | Europe | Aurum |
| 460031018 | born in Saudi Arabia | Asia | Aurum |
| 460036011 | born in Singapore | Asia | Aurum |
| 460042010 | born in South Korea | Asia | Aurum |
| 460044011 | born in Sri Lanka | Asia | Aurum |
| 460053016 | born in Syria | Asia | Aurum |
| 460054010 | born in Taiwan | Asia | Aurum |
| 460055011 | born in Tajikistan | Asia | Aurum |
| 460057015 | born in Thailand | Asia | Aurum |
| 460064018 | born in Turkey | Europe | Aurum |
| 460065017 | born in Turkmenistan | Asia | Aurum |
| 460070012 | born in United Arab Emirates | Asia | Aurum |
| 460072016 | born in Uzbekistan | Asia | Aurum |
| 460076018 | born in Vietnam | Asia | Aurum |
| 460108015 | born in Yemen | Asia | Aurum |
| 2361321000000116 | born in Christmas Island | Asia | Aurum |
| 2361361000000112 | born in Cocos (Keeling) Islands | Asia | Aurum |
| 2363351000000110 | born in Macao | Asia | Aurum |
| 459871016 | country of birth (American) | Americas | Aurum |
| 459887018 | born in Argentina | Americas | Aurum |
| 459897010 | born in Belize | Americas | Aurum |
| 459901016 | born in Bolivia | Americas | Aurum |
| 459904012 | born in Brazil | Americas | Aurum |
| 459905013 | born in British Guyana | Americas | Aurum |
| 459913014 | born in Canada | Americas | Aurum |
| 459918017 | born in Chile | Americas | Aurum |
| 459920019 | born in Columbia | Americas | Aurum |
| 459923017 | born in Costa Rica | Americas | Aurum |
| 459933013 | born in Ecuador | Americas | Aurum |
| 459935018 | born in El Salvador | Americas | Aurum |
| 459949010 | born in Grenada | Americas | Aurum |
| 459950010 | born in Guatemala | Americas | Aurum |
| 459953012 | born in Guyana | Americas | Aurum |
| 459955017 | born in Honduras | Americas | Aurum |
| 459996019 | born in Mexico | Americas | Aurum |
| 460006019 | born in Nicaragua | Americas | Aurum |
| 460015014 | born in Panama | Americas | Aurum |
| 460017018 | born in Paraguay | Americas | Aurum |
| 460018011 | born in Peru | Americas | Aurum |
| 460049018 | born in Suriname | Americas | Aurum |
| 460067013 | born in USA | Americas | Aurum |
| 460071011 | born in Uruguay | Americas | Aurum |
| 460075019 | born in Venezuela | Americas | Aurum |
| 459872011 | country of birth (African) | Africa | Aurum |
| 459881017 | born in Algeria | Africa | Aurum |
| 459883019 | born in Angola | Africa | Aurum |
| 459899013 | born in Benin | Africa | Aurum |
| 459903018 | born in Botswana | Africa | Aurum |
| 459908010 | born in Burkina Faso | Africa | Aurum |
| 459910012 | born in Burundi | Africa | Aurum |
| 459912016 | born in Cameroon | Africa | Aurum |
| 459914015 | born in Cape Verde Islands | Africa | Aurum |
| 459915019 | born in Central African Republic | Africa | Aurum |
| 459916018 | born in Chad | Africa | Aurum |
| 459921015 | born in Comoros Islands | Africa | Aurum |
| 459922010 | born in Congo | Africa | Aurum |
| 459930011 | born in Djibouti | Africa | Aurum |
| 459934019 | born in Egypt | Africa | Aurum |
| 459937014 | born in Equatorial Guinea | Africa | Aurum |
| 459939012 | born in Ethiopia | Africa | Aurum |
| 459943011 | born in Gabon | Africa | Aurum |
| 459944017 | born in Gambia | Africa | Aurum |
| 459947012 | born in Ghana | Africa | Aurum |
| 459951014 | born in Guinea Bissau | Africa | Aurum |
| 459952019 | born in Guinea Republic | Africa | Aurum |
| 459966012 | born in Ivory Coast | Africa | Aurum |
| 459971017 | born in Kenya | Africa | Aurum |
| 459979015 | born in Lesotho | Africa | Aurum |
| 459980017 | born in Liberia | Africa | Aurum |
| 459981018 | born in Libya | Africa | Aurum |
| 459985010 | born in Madagascar | Africa | Aurum |
| 459986011 | born in Malawi | Africa | Aurum |
| 459994016 | born in Mauritania | Africa | Aurum |
| 459995015 | born in Mauritius | Africa | Aurum |
| 460000013 | born in Morocco | Africa | Aurum |
| 460001012 | born in Mozambique | Africa | Aurum |
| 460002017 | born in Namibia | Africa | Aurum |
| 460007011 | born in Niger | Africa | Aurum |
| 460008018 | born in Nigeria | Africa | Aurum |
| 460028019 | born in Rwanda | Africa | Aurum |
| 460030017 | born in Sao Tome and Principe | Africa | Aurum |
| 460033015 | born in Senegal | Africa | Aurum |
| 460035010 | born in Sierra Leone | Africa | Aurum |
| 460040019 | born in Somalia | Africa | Aurum |
| 460041015 | born in South Africa | Africa | Aurum |
| 460048014 | born in Sudan | Africa | Aurum |
| 460050018 | born in Swaziland | Africa | Aurum |
| 460056012 | born in Tanzania | Africa | Aurum |
| 2476508013 | born in The Gambia | Africa | Aurum |
| 460063012 | born in Tunisia | Africa | Aurum |
| 460068015 | born in Uganda | Africa | Aurum |
| 460080011 | born in Zaire | Africa | Aurum |
| 460081010 | born in Zambia | Africa | Aurum |
| 460082015 | born in Zimbabwe | Africa | Aurum |
| 314501000000119 | born in Eritrea | Africa | Aurum |
| 1650111000000116 | born in Democratic Republic of Congo | Africa | Aurum |
| 2363391000000119 | born in Western Sahara | Africa | Aurum |
| 459873018 | country of birth (Australasian) | Australasia | Aurum |
| 459889015 | born in Australia | Australasia | Aurum |
| 460005015 | born in new Zealand | Australasia | Aurum |
| 459874012 | country of birth (Atlantic) | Americas | Aurum |
| 459884013 | born in Antigua and Barbuda | Americas | Aurum |
| 459892016 | born in Bahamas | Americas | Aurum |
| 459895019 | born in Barbados | Americas | Aurum |
| 459925012 | born in Cuba | Americas | Aurum |
| 459931010 | born in Dominican Republic | Americas | Aurum |
| 459954018 | born in Haiti | Americas | Aurum |
| 459967015 | born in Jamaica | Americas | Aurum |
| 460022018 | born in Puerto Rico | Americas | Aurum |
| 460045012 | born in St. Kitts and Nevis | Americas | Aurum |
| 460046013 | born in St. Lucia | Americas | Aurum |
| 460047016 | born in St. Vincent | Americas | Aurum |
| 460060010 | born in Togo | Africa | Aurum |
| 460062019 | born in Trinidad and Tobago | Americas | Aurum |
| 1117841000000115 | born in Dominica | Americas | Aurum |
| 1715661000000119 | born in Aruba | Americas | Aurum |
| 2359621000000117 | born in United States Virgin Islands | Americas | Aurum |
| 2359791000000114 | born in Saint Vincent and the Grenadines | Americas | Aurum |
| 2360011000000118 | born in Sint Maarten | Americas | Aurum |
| 2360051000000119 | born in saint-martin | Americas | Aurum |
| 2361081000000117 | born in Bonaire, Sint Eustatius and Saba | Americas | Aurum |
| 2369611000000119 | born in Curacao | Americas | Aurum |
| 459875013 | country of birth (Pacific) | Australasia | Aurum |
| 459940014 | born in Fiji | Australasia | Aurum |
| 459972012 | born in Kiribati | Australasia | Aurum |
| 460003010 | born in Nauru | Australasia | Aurum |
| 460016010 | born in Papua New Guinea | Australasia | Aurum |
| 460034014 | born in Seychelles | Africa | Aurum |
| 460039016 | born in Solomon Islands | Australasia | Aurum |
| 460061014 | born in Tonga | Australasia | Aurum |
| 2476509017 | born in Tuvalu | Australasia | Aurum |
| 460073014 | born in Vanuatu | Australasia | Aurum |
| 460078017 | born in Western Samoa | Australasia | Aurum |
| 2359521000000118 | born in Samoa | Australasia | Aurum |
| 2361021000000118 | born in American Samoa | Australasia | Aurum |
| 2359851000000114 | born in United States minor outlying islands | Australasia | Aurum |
| 2359891000000118 | born in Tokelau | Australasia | Aurum |
| 2361401000000115 | born in Cook Islands | Australasia | Aurum |
| 2361481000000113 | born in Guam | Australasia | Aurum |
| 2361641000000119 | born in Federated States of Micronesia | Australasia | Aurum |
| 2361681000000110 | born in Marshall Islands | Australasia | Aurum |
| 2361721000000115 | born in Niue | Australasia | Aurum |
| 2361761000000111 | born in Norfolk Island | Australasia | Aurum |
| 2361801000000118 | born in Northern Mariana Islands | Australasia | Aurum |
| 2361841000000115 | born in Palau | Australasia | Aurum |
| 2362501000000110 | born in Antarctica | Australasia | Aurum |
| 639751000000112 | born in British Overseas Territory | Migrant or unknown | Aurum |
| 639841000000119 | born in Montserrat | Americas | Aurum |
| 639901000000113 | born in Bermuda | Americas | Aurum |
| 1117781000000119 | born in Anguilla | Americas | Aurum |
| 2359751000000118 | born in British Virgin Islands | Americas | Aurum |
| 2359971000000113 | born in Turks and Caicos Islands | Americas | Aurum |
| 2360131000000113 | born in Saint Helena, Ascension and Tristan da Cunha | Americas | Aurum |
| 2360171000000110 | born in South Georgia and the South Sandwich Islands | Americas | Aurum |
| 2360291000000116 | born in Falkland Islands | Americas | Aurum |
| 2361121000000119 | born in British Indian Ocean Territory | Asia | Aurum |
| 2361161000000110 | born in Cayman Islands | Americas | Aurum |
| 2361281000000112 | born in Pitcairn Islands | Australasia | Aurum |
| 1677931000006116 | born in French overseas region, department, collectivity or territory | Migrant or unknown | Aurum |
| 647801000000112 | born in Martinique | Americas | Aurum |
| 2360091000000110 | born in saint Pierre and Miquelon | Americas | Aurum |
| 2359561000000114 | born in Wallis and Futuna | Australasia | Aurum |
| 2360211000000113 | born in French Polynesia | Australasia | Aurum |
| 2360251000000112 | born in French Guiana | Americas | Aurum |
| 2361201000000119 | born in French southern territories | Australasia | Aurum |
| 2361241000000116 | born in Mayotte | Africa | Aurum |
| 2361441000000117 | born in Guadeloupe | Americas | Aurum |
| 2369431000000113 | born in Reunion | Africa | Aurum |
| 2363431000000110 | born in New Caledonia | Australasia | Aurum |
| 1227731017 | [v]social migrant | Migrant or unknown | Aurum |

## Code list for determining language in CPRD

The CPRD Code Browser was used to generate a list of codes, both in CPRD Gold and CPRD Aurum. The search strategy included terms such as “*language*”, “*interpreter*”, etc.

All codes that are related to language were considered. The final code lists had 259 codes for CPRD Gold and 985 codes for CPRD Aurum.

| **Medcode** | **Description** | **Main Spoken Language Category** | **Source** |
| --- | --- | --- | --- |
| 96267 | main spoken language Marathi | South Asian | Gold |
| 101620 | main spoken language Malagasy | Africa | Gold |
| 63944 | Hindi as a second language | South Asian | Gold |
| 108184 | Turkmen language interpreter needed | West & Central Asia | Gold |
| 96868 | main spoken language Afar | Africa | Gold |
| 98285 | main spoken language Filipino | East Asian | Gold |
| 105454 | Lingala as a second language | Africa | Gold |
| 96268 | main spoken language Kannada | South Asian | Gold |
| 52162 | Spanish as a second language | European | Gold |
| 96768 | Somali as a second language | Africa | Gold |
| 115124 | main spoken language Bislama | Australasia | Gold |
| 63945 | Chinese as a second language | East Asian | Gold |
| 26043 | using British Sign Language | UK languages | Gold |
| 56977 | main spoken language Welsh | UK languages | Gold |
| 113132 | Inuktitut language interpreter needed | Americas | Gold |
| 106806 | Akan as a second language | Africa | Gold |
| 102259 | main spoken language Bashkir | European | Gold |
| 102877 | main spoken language Tswana | Africa | Gold |
| 115123 | Luganda language interpreter needed | Africa | Gold |
| 25410 | Vietnamese language | East Asian | Gold |
| 102218 | main spoken language Assamese | South Asian | Gold |
| 114188 | main spoken language Hindko | South Asian | Gold |
| 24296 | language Hindi | South Asian | Gold |
| 86389 | Tigrinya as a second language | Africa | Gold |
| 46014 | main spoken language Kurdish | West & Central Asia | Gold |
| 26361 | main spoken language Urdu | South Asian | Gold |
| 111820 | Ethiopian language interpreter needed | Africa | Gold |
| 102127 | main spoken language Dzongkha | South Asian | Gold |
| 24668 | language not recorded | English/Unknown | Gold |
| 46973 | main spoken language Lingala | Africa | Gold |
| 103821 | Croatian as a second language | European | Gold |
| 26337 | main spoken language Arabic | West & Central Asia | Gold |
| 26196 | language Pashtu | West & Central Asia | Gold |
| 102007 | main spoken language Greenlandic | European | Gold |
| 101788 | main spoken language Corsican | European | Gold |
| 108098 | first language not English | English/Unknown | Gold |
| 96146 | main spoken language Irish | UK languages | Gold |
| 100743 | main spoken language Galician | European | Gold |
| 96559 | main spoken language Burmese | East Asian | Gold |
| 98762 | main spoken language Aymara | Americas | Gold |
| 54410 | main spoken language Croatian | European | Gold |
| 96370 | main spoken language Maltese | European | Gold |
| 63933 | Lithuanian as a second language | European | Gold |
| 105960 | main spoken language Tajik | West & Central Asia | Gold |
| 36852 | main spoken language Japanese | East Asian | Gold |
| 96240 | main spoken language Sandhi | South Asian | Gold |
| 36862 | main spoken language Serbian | European | Gold |
| 101614 | Telugu language interpreter needed | South Asian | Gold |
| 111303 | Romansh language interpreter needed | European | Gold |
| 95463 | Arabic as a second language | West & Central Asia | Gold |
| 96164 | Igbo as a second language | Africa | Gold |
| 105785 | Amharic as a second language | Africa | Gold |
| 96784 | main spoken language Uzbek | West & Central Asia | Gold |
| 111919 | main spoken language Sango | Africa | Gold |
| 105523 | main spoken language Tetum | East Asian | Gold |
| 26335 | main spoken language Vietnamese | East Asian | Gold |
| 111709 | main spoken language Swati | Africa | Gold |
| 63938 | Polish as a second language | European | Gold |
| 115316 | Assamese language interpreter needed | South Asian | Gold |
| 111734 | main spoken language Nauru | Australasia | Gold |
| 98194 | main spoken language Kinyarwanda | Africa | Gold |
| 111789 | main spoken language Zhuang | East Asian | Gold |
| 24741 | language Bengali | South Asian | Gold |
| 96877 | supplemental main language spoken | English/Unknown | Gold |
| 96928 | main spoken language Estonian | European | Gold |
| 54415 | main spoken language Korean | East Asian | Gold |
| 115923 | Indonesian language interpreter needed | East Asian | Gold |
| 26247 | main spoken language Spanish | European | Gold |
| 57462 | Creole language | Americas | Gold |
| 96152 | main spoken language Romanian | European | Gold |
| 96873 | main spoken language Zulu | Africa | Gold |
| 96375 | Thai as a second language | East Asian | Gold |
| 24353 | language nos | English/Unknown | Gold |
| 63931 | Gujarati as a second language | South Asian | Gold |
| 96230 | main spoken language Slovenian | European | Gold |
| 100813 | interlingua language interpreter needed |  | Gold |
| 97206 | Swedish as a second language | European | Gold |
| 95970 | main spoken language Persian | West & Central Asia | Gold |
| 47643 | main spoken language Sinhala | South Asian | Gold |
| 47029 | main spoken language Farsi | West & Central Asia | Gold |
| 57765 | Russian as a second language | European | Gold |
| 95897 | main spoken language Bulgarian | European | Gold |
| 89689 | Turkish as a second language | West & Central Asia | Gold |
| 96611 | main spoken language Macedonian | European | Gold |
| 32728 | main spoken language Portuguese | European | Gold |
| 96560 | main spoken language Finnish | European | Gold |
| 97273 | main spoken language Xhosa | Africa | Gold |
| 64391 | main spoken language Gaelic | UK languages | Gold |
| 47641 | main spoken language Swahili | Africa | Gold |
| 99556 | Hausa as a second language | Africa | Gold |
| 47644 | main spoken language Tigrinya | Africa | Gold |
| 97083 | main spoken language Azerbaijani | European | Gold |
| 96857 | main spoken language Oromo | Africa | Gold |
| 101189 | main spoken language central Khmer | East Asian | Gold |
| 101284 | Nepali language interpreter needed | South Asian | Gold |
| 63932 | English as a second language | English/Unknown | Gold |
| 101220 | main spoken language Dari | West & Central Asia | Gold |
| 25609 | main spoken language Thai | East Asian | Gold |
| 89462 | interpreter needed - British sign language | UK languages | Gold |
| 99794 | Tsonga language interpreter needed | Africa | Gold |
| 96147 | main spoken language Panjabi | South Asian | Gold |
| 63940 | French as a second language | European | Gold |
| 25616 | main spoken language Malayalam | South Asian | Gold |
| 25829 | main spoken language Punjabi | South Asian | Gold |
| 97131 | main spoken language Sundanese | English/Unknown | Gold |
| 47646 | main spoken language Igbo | Africa | Gold |
| 112321 | main spoken language Tatar | European | Gold |
| 25665 | main spoken language French | European | Gold |
| 63939 | German as a second language | European | Gold |
| 102173 | Tagalog as a second language | East Asian | Gold |
| 24691 | language Urdu | South Asian | Gold |
| 100716 | main spoken language Inuktitut | Americas | Gold |
| 105079 | Catalan language interpreter needed | European | Gold |
| 101659 | main spoken language interlingua |  | Gold |
| 100828 | main spoken language Belarusian | European | Gold |
| 47627 | main spoken language Sylheti | South Asian | Gold |
| 98604 | main spoken language Icelandic | European | Gold |
| 54409 | main spoken language Yoruba | Africa | Gold |
| 97644 | Bulgarian language interpreter needed | European | Gold |
| 93691 | Dutch as a second language | European | Gold |
| 97997 | main spoken language Catalan | European | Gold |
| 95978 | main spoken language Nepali | South Asian | Gold |
| 98201 | Scottish Gaelic as a second language | UK languages | Gold |
| 101761 | main spoken language Iban | South Asian | Gold |
| 100714 | main spoken language Fijian | Australasia | Gold |
| 96376 | main spoken language Malay | East Asian | Gold |
| 111739 | visual frame sign language interpreter needed | UK languages | Gold |
| 100013 | main spoken language Slovak | European | Gold |
| 112222 | Sundanese language interpreter needed | English/Unknown | Gold |
| 98841 | Romanian language interpreter needed | European | Gold |
| 111788 | Moldavian language interpreter needed | European | Gold |
| 98510 | main spoken language Kalaallit | European | Gold |
| 96148 | main spoken language Afrikaans | Africa | Gold |
| 103219 | main spoken language Samoan | Australasia | Gold |
| 104635 | Latvian language interpreter needed | European | Gold |
| 25472 | main spoken language Cantonese | East Asian | Gold |
| 57755 | main spoken language Hebrew | West & Central Asia | Gold |
| 98062 | Burmese language interpreter needed | East Asian | Gold |
| 98202 | Norwegian as a second language | European | Gold |
| 97597 | Yoruba as a second language | Africa | Gold |
| 26464 | main spoken language Czech | European | Gold |
| 95940 | main spoken language Latvian | European | Gold |
| 112961 | Armenian language interpreter needed | West & Central Asia | Gold |
| 47630 | main spoken language German | European | Gold |
| 25423 | main spoken language Albanian | European | Gold |
| 32427 | main spoken language Russian | European | Gold |
| 97041 | main spoken language Turkmen | West & Central Asia | Gold |
| 54416 | main spoken language Iba | South Asian | Gold |
| 112260 | main spoken language Rundi | Africa | Gold |
| 99501 | Ukrainian as a second language | European | Gold |
| 102184 | main spoken language Abkhazian | European | Gold |
| 98132 | main spoken language Lao | East Asian | Gold |
| 97038 | main spoken language Mongolian | East Asian | Gold |
| 54413 | main spoken language Akan | Africa | Gold |
| 96290 | main spoken language Armenian | West & Central Asia | Gold |
| 66685 | main spoken language Ukrainian | European | Gold |
| 111874 | main spoken language Yiddish | European | Gold |
| 25606 | main spoken language English | English/Unknown | Gold |
| 44297 | using Makaton sign language | UK languages | Gold |
| 63934 | Italian as a second language | European | Gold |
| 96589 | interpreter needed - Makaton sign language | UK languages | Gold |
| 24295 | language Punjabi | South Asian | Gold |
| 97439 | main spoken language southern Sotho | Africa | Gold |
| 97212 | main spoken language Georgian | European | Gold |
| 32456 | main spoken language Somali | Africa | Gold |
| 104886 | main spoken language Ndebele | Africa | Gold |
| 95968 | main spoken language Serbo-Croatian | European | Gold |
| 95462 | Vietnamese as a second language | East Asian | Gold |
| 101038 | main spoken language Konkani | South Asian | Gold |
| 97149 | Kurdish as a second language | West & Central Asia | Gold |
| 91758 | Shona as a second language | Africa | Gold |
| 96289 | main spoken language Danish | European | Gold |
| 97297 | main spoken language Oriya | South Asian | Gold |
| 97685 | main spoken language Tsonga | Africa | Gold |
| 46861 | main spoken language Tamil | South Asian | Gold |
| 100759 | Slovenian language interpreter needed | European | Gold |
| 100452 | preferred method of communication: British sign language | UK languages | Gold |
| 96600 | additional main spoken language | English/Unknown | Gold |
| 91761 | Swahili as a second language | Africa | Gold |
| 46325 | main spoken language Lithuanian | European | Gold |
| 100707 | main spoken language Faroese | European | Gold |
| 101814 | Punjabi language interpreter needed | South Asian | Gold |
| 98439 | Portuguese as a second language | European | Gold |
| 100949 | main spoken language Bihari | South Asian | Gold |
| 97298 | Persian language interpreter needed | West & Central Asia | Gold |
| 98581 | Serbian as a second language | European | Gold |
| 26078 | main spoken language Shona | Africa | Gold |
| 99712 | main spoken language Uighur | East Asian | Gold |
| 96485 | main spoken language Brawa | Africa | Gold |
| 24712 | language Gujarati | South Asian | Gold |
| 97595 | main spoken language Kazakh | West & Central Asia | Gold |
| 95974 | main spoken language Sinhalese | South Asian | Gold |
| 96163 | main spoken language Telugu | South Asian | Gold |
| 58193 | main spoken language Hausa | Africa | Gold |
| 54414 | main spoken language Dutch | European | Gold |
| 98255 | main spoken language Māori | Australasia | Gold |
| 98809 | Hungarian language interpreter needed | European | Gold |
| 115120 | using sign language to communicate with client | UK languages | Gold |
| 96317 | main spoken language Quechua | Americas | Gold |
| 47628 | main spoken language Amharic | Africa | Gold |
| 97015 | main spoken language Kashmiri | South Asian | Gold |
| 104071 | main spoken language Bamun | Africa | Gold |
| 100011 | main spoken language Javanese | East Asian | Gold |
| 98215 | main spoken language Moldavian | European | Gold |
| 98070 | main spoken language Guarani | Americas | Gold |
| 98576 | Malayalam as a second language | South Asian | Gold |
| 113326 | main spoken language Bamoun | Africa | Gold |
| 97789 | main spoken language Esperanto |  | Gold |
| 102128 | main spoken language Breton | European | Gold |
| 48002 | main spoken language Gujarati | South Asian | Gold |
| 95877 | Welsh as a second language | UK languages | Gold |
| 47631 | main spoken language Tagalog | East Asian | Gold |
| 96223 | main spoken language Twi | Africa | Gold |
| 36980 | main spoken language Italian | European | Gold |
| 32776 | main spoken language Mandarin | East Asian | Gold |
| 72379 | main spoken language Kutchi | South Asian | Gold |
| 22294 | main spoken language Turkish | West & Central Asia | Gold |
| 111921 | Georgian language interpreter needed | European | Gold |
| 56879 | main spoken language Flemish | European | Gold |
| 102129 | main spoken language interlingua |  | Gold |
| 25802 | main spoken language Bengali | South Asian | Gold |
| 96634 | main spoken language Tongan | Australasia | Gold |
| 97574 | main spoken language Basque | European | Gold |
| 69139 | main spoken language Ethiopian | Africa | Gold |
| 47007 | main spoken language French creole | Americas | Gold |
| 97274 | main spoken language Indonesian | East Asian | Gold |
| 52200 | Mirpuri language | South Asian | Gold |
| 100438 | Macedonian language interpreter needed | European | Gold |
| 101061 | Irish Gaelic as a second language | UK languages | Gold |
| 91936 | Bengali as a second language | South Asian | Gold |
| 105171 | Hebrew as a second language | West & Central Asia | Gold |
| 63941 | Japanese as a second language | East Asian | Gold |
| 63937 | Czech as a second language | European | Gold |
| 69153 | main spoken language Luganda | Africa | Gold |
| 104901 | Brawa language interpreter needed | Africa | Gold |
| 96088 | Urdu as a second language | South Asian | Gold |
| 46029 | main spoken language Hindi | South Asian | Gold |
| 100990 | Albanian as a second language | European | Gold |
| 109896 | main spoken language Gujarati | South Asian | Gold |
| 96558 | main spoken language Tibetan | South Asian | Gold |
| 57758 | main spoken language Norwegian | European | Gold |
| 97440 | main spoken language Romansh | European | Gold |
| 24881 | main spoken language polish | European | Gold |
| 46974 | main spoken language Greek | European | Gold |
| 96858 | main spoken language Occitan | European | Gold |
| 98887 | Greek as a second language | European | Gold |
| 103200 | main spoken language Frisian | European | Gold |
| 98195 | Tamil as a second language | South Asian | Gold |
| 109489 | Kinyarwanda language interpreter needed | Africa | Gold |
| 104678 | Oromo language interpreter needed | Africa | Gold |
| 64948 | main spoken language Patois | Americas | Gold |
| 54417 | main spoken language Swedish | European | Gold |
| 91422 | main spoken language Hakka | East Asian | Gold |
| 58643 | main spoken language Pashto | West & Central Asia | Gold |
| 97039 | main spoken language Wolof | Africa | Gold |
| 96041 | main spoken language Hungarian | European | Gold |
| 23523 | English as a second language | English/Unknown | Gold |
| 105529 | main spoken language Aragonese | European | Gold |
| 5838931000006111 | Harari language | Africa | Aurum |
| 1778531000006117 | preferred communication language: Hakka | East Asian | Aurum |
| 5843931000006117 | Algonkin language | Americas | Aurum |
| 1861891000006119 | preferred written language: Belarusian | European | Aurum |
| 672471000000117 | main spoken language Kannada | South Asian | Aurum |
| 1854241000006117 | preferred written language: Gaelic | UK languages | Aurum |
| 1929141000006115 | main spoken language Sorani Kurdish | West & Central Asia | Aurum |
| 4538971000006118 | Urdu language | South Asian | Aurum |
| 1863171000006112 | preferred written language: western Frisian | European | Aurum |
| 1862431000006113 | preferred written language: Kazakh | West & Central Asia | Aurum |
| 1778821000006117 | preferred communication language: Tamil | South Asian | Aurum |
| 5844251000006117 | Mayan language | Americas | Aurum |
| 1854631000006112 | preferred written language: Yoruba | Africa | Aurum |
| 1778851000006114 | preferred communication language: Turkish | West & Central Asia | Aurum |
| 1862751000006114 | preferred written language: Nepali | South Asian | Aurum |
| 672411000000110 | main spoken language Javanese | East Asian | Aurum |
| 2160084010 | main spoken language Patois | Americas | Aurum |
| 992881000006116 | Gaelic as a second language | UK languages | Aurum |
| 8356291000006114 | main spoken language Scottish Gaelic | UK languages | Aurum |
| 678271000000115 | Kannada language interpreter needed | South Asian | Aurum |
| 1862811000006116 | preferred written language: Oromo | Africa | Aurum |
| 2160075010 | main spoken language Akan | Africa | Aurum |
| 5843291000006113 | Yao language - Bantu | Africa | Aurum |
| 5841451000006119 | Konkani language | South Asian | Aurum |
| 2160073015 | main spoken language Hakka | East Asian | Aurum |
| 673191000000119 | main spoken language Maltese | European | Aurum |
| 309521000000110 | Portuguese as a second language | European | Aurum |
| 1854351000006114 | preferred written language: Korean | East Asian | Aurum |
| 5839821000006110 | central and south American Indian language | Americas | Aurum |
| 5845661000006118 | Chin language | East Asian | Aurum |
| 674151000000112 | main spoken language southern Sotho | Africa | Aurum |
| 5841671000006110 | Baluchi language | South Asian | Aurum |
| 309121000000116 | Arabic as a second language | West & Central Asia | Aurum |
| 1862861000006118 | preferred written language: Romansh | European | Aurum |
| 8063471000006113 | Croatian language interpreter needed | European | Aurum |
| 1861761000006117 | preferred written language: afar | Africa | Aurum |
| 1778741000006117 | preferred communication language: Serbian/Croatian | European | Aurum |
| 1862211000006115 | preferred written language: Galician | European | Aurum |
| 1854301000006110 | preferred written language: Hebrew | West & Central Asia | Aurum |
| 672111000000115 | main spoken language interlingua |  | Aurum |
| 8064061000006113 | Pashto language interpreter needed | West & Central Asia | Aurum |
| 2160085011 | main spoken language Serbian | European | Aurum |
| 5842601000006114 | Banda language | Africa | Aurum |
| 1863201000006111 | preferred written language: Yiddish | European | Aurum |
| 684881000000117 | Tsonga language interpreter needed | Africa | Aurum |
| 8063281000006112 | Akan language interpreter needed | Africa | Aurum |
| 687271000000112 | Nauru language interpreter needed | Australasia | Aurum |
| 6994291000006111 | language barrier | English/Unknown | Aurum |
| 1778511000006111 | preferred communication language: Greek | European | Aurum |
| 310171000000110 | Thai as a second language | East Asian | Aurum |
| 5838881000006117 | Ethiopic language | Africa | Aurum |
| 5843151000006110 | Sotho language | Africa | Aurum |
| 1861791000006113 | preferred written language: Armenian | West & Central Asia | Aurum |
| 675411000000117 | main spoken language Zhuang | East Asian | Aurum |
| 1863101000006118 | preferred written language: Twi | Africa | Aurum |
| 2160074014 | main spoken language Hebrew | West & Central Asia | Aurum |
| 1778491000006117 | preferred communication language: Gaelic | UK languages | Aurum |
| 2159254019 | main spoken language Japanese | East Asian | Aurum |
| 251253015 | language Urdu | South Asian | Aurum |
| 309411000000115 | Japanese as a second language | East Asian | Aurum |
| 1706141000006110 | main spoken language Faroese | European | Aurum |
| 682231000000113 | Danish language interpreter needed | European | Aurum |
| 251238011 | English as a second language | English/Unknown | Aurum |
| 457311012 | Creole language | Americas | Aurum |
| 1854611000006118 | preferred written language: Vietnamese | East Asian | Aurum |
| 5842091000006112 | Tagalog language | East Asian | Aurum |
| 5841231000006110 | Ukrainian language | European | Aurum |
| 5842211000006114 | Fijian language | Australasia | Aurum |
| 687071000000116 | Nepali language interpreter needed | South Asian | Aurum |
| 1778401000006112 | preferred communication language: Creole | Americas | Aurum |
| 5843491000006112 | Kwa language | Africa | Aurum |
| 644471000000110 | Persian language interpreter needed | West & Central Asia | Aurum |
| 1778631000006118 | preferred communication language: Luganda | Africa | Aurum |
| 2159252015 | main spoken language Greek | European | Aurum |
| 674451000000119 | main spoken language Slovenian | European | Aurum |
| 1863131000006114 | preferred written language: Uzbek | West & Central Asia | Aurum |
| 460099019 | main spoken language Punjabi | South Asian | Aurum |
| 309241000000118 | French as a second language | European | Aurum |
| 1854151000006111 | preferred written language: Creole | Americas | Aurum |
| 674391000000118 | main spoken language Swati | Africa | Aurum |
| 8064421000006110 | Sylheti language interpreter needed | South Asian | Aurum |
| 1854171000006118 | preferred written language: English | English/Unknown | Aurum |
| 309291000000111 | Greek as a second language | European | Aurum |
| 671351000000111 | main spoken language Estonian | European | Aurum |
| 1854651000006117 | preferred written language: bilingual Welsh/English | UK languages | Aurum |
| 1778801000006110 | preferred communication language: Sylheti | South Asian | Aurum |
| 1706121000006115 | main spoken language Dhivehi | South Asian | Aurum |
| 1854471000006112 | preferred written language: Russian | European | Aurum |
| 959041000006115 | language barrier | English/Unknown | Aurum |
| 2159250011 | main spoken language Croatian | European | Aurum |
| 993301000006112 | language of interpreter - Kutchi | South Asian | Aurum |
| 451305019 | language interpreter | English/Unknown | Aurum |
| 1706201000006115 | main spoken language Herero | Africa | Aurum |
| 670661000000114 | main spoken language Bihari | South Asian | Aurum |
| 1854401000006118 | preferred written language: Mandarin | East Asian | Aurum |
| 5841471000006112 | Marathi language | South Asian | Aurum |
| 5845821000006111 | Tibetan language | South Asian | Aurum |
| 2160063017 | main spoken language Brawa | Africa | Aurum |
| 682431000000114 | Belarusian language interpreter needed | European | Aurum |
| 310111000000117 | Shona as a second language | Africa | Aurum |
| 5841631000006112 | Sinhalese language | South Asian | Aurum |
| 1706411000006116 | main spoken language Ojibwe | Americas | Aurum |
| 5840331000006112 | Nuer language | Africa | Aurum |
| 314871000000112 | Scottish Gaelic as a second language | UK languages | Aurum |
| 1488809010 | main spoken language Shona | Africa | Aurum |
| 309141000000111 | Bengali as a second language | South Asian | Aurum |
| 5841501000006117 | Pakistani Punjabi language | South Asian | Aurum |
| 337703011 | interpreter for British sign language | UK languages | Aurum |
| 1706471000006113 | main spoken language Sardinian | European | Aurum |
| 673551000000118 | main spoken language Nepali | South Asian | Aurum |
| 1861811000006112 | preferred written language: avarice | European | Aurum |
| 1706281000006112 | main spoken language Kanyama | Africa | Aurum |
| 5840991000006112 | Greek language | European | Aurum |
| 251257019 | language nos | English/Unknown | Aurum |
| 677591000000115 | Luganda language interpreter needed | Africa | Aurum |
| 672051000000117 | main spoken language Indonesian | East Asian | Aurum |
| 310051000000119 | Urdu as a second language | South Asian | Aurum |
| 1706171000006119 | main spoken language Manx | UK languages | Aurum |
| 674211000000117 | main spoken language Tswana | Africa | Aurum |
| 5841181000006115 | eastern Slavic language | European | Aurum |
| 1778761000006118 | preferred communication language: Somali | Africa | Aurum |
| 678691000000118 | Inuktitut language interpreter needed | Americas | Aurum |
| 309391000000115 | Igbo as a second language | Africa | Aurum |
| 1854071000006119 | preferred written language: Akan (Ashanti) | Africa | Aurum |
| 7511141000006115 | main spoken language Faroese | European | Aurum |
| 1778611000006112 | preferred communication language: Kurdish | West & Central Asia | Aurum |
| 1861901000006115 | preferred written language: Bihari | South Asian | Aurum |
| 5843971000006119 | Cree language | Americas | Aurum |
| 642161000000117 | main spoken language Romanian | European | Aurum |
| 1706481000006111 | main spoken language Tahitian | Australasia | Aurum |
| 1854191000006117 | preferred written language: Farsi (Persian) | West & Central Asia | Aurum |
| 6750051000006116 | main spoken language Croat | European | Aurum |
| 310071000000111 | Yoruba as a second language | Africa | Aurum |
| 5840771000006114 | Scottish Gaelic language | UK languages | Aurum |
| 1862371000006115 | preferred written language: Irish | UK languages | Aurum |
| 1854371000006116 | preferred written language: Lingala | Africa | Aurum |
| 1706031000006118 | main spoken language Bambara | Africa | Aurum |
| 8063301000006111 | Albanian language interpreter needed | European | Aurum |
| 684441000000111 | Oromo language interpreter needed | Africa | Aurum |
| 1862301000006114 | preferred written language: Icelandic | European | Aurum |
| 460103012 | main spoken language Swahili | Africa | Aurum |
| 1162181000000117 | preferred method of communication: British sign language | UK languages | Aurum |
| 685971000000111 | Sindhi language interpreter needed | South Asian | Aurum |
| 992871000006119 | Sylheti as a second language | South Asian | Aurum |
| 1706251000006116 | main spoken language Kikuyu | Africa | Aurum |
| 5844431000006111 | Chinantec language | Americas | Aurum |
| 1862061000006113 | preferred written language: Corsican | European | Aurum |
| 1661561000000112 | main spoken language Dari | West & Central Asia | Aurum |
| 673251000000116 | main spoken language Māori | Australasia | Aurum |
| 1778871000006116 | preferred communication language: Vietnamese | East Asian | Aurum |
| 1862801000006119 | preferred written language: Oriya | South Asian | Aurum |
| 1656471000006110 | main spoken language Romanian | European | Aurum |
| 5839431000006111 | Uzbek language | West & Central Asia | Aurum |
| 8063831000006112 | Korean language interpreter needed | East Asian | Aurum |
| 1862391000006119 | preferred written language: Kalaallit | European | Aurum |
| 1696131000006112 | main spoken language Slovak | European | Aurum |
| 686571000000111 | Quechua language interpreter needed | Americas | Aurum |
| 4538831000006114 | Bengali language | South Asian | Aurum |
| 1854591000006112 | preferred written language: Turkish | West & Central Asia | Aurum |
| 5841731000006115 | Tajik language | West & Central Asia | Aurum |
| 1854541000006115 | preferred written language: Sylheti | South Asian | Aurum |
| 1862521000006114 | preferred written language: Lao | East Asian | Aurum |
| 670461000000111 | main spoken language Bashkir | European | Aurum |
| 1861931000006111 | preferred written language: Bosnian | European | Aurum |
| 681271000000116 | Frisian language interpreter needed | European | Aurum |
| 1862251000006119 | preferred written language: Herero | Africa | Aurum |
| 1778751000006115 | preferred communication language: Sinhala | South Asian | Aurum |
| 5839871000006111 | Guarani language | Americas | Aurum |
| 678751000000117 | interlingua language interpreter needed |  | Aurum |
| 1778411000006110 | preferred communication language: Dutch | European | Aurum |
| 1862031000006116 | preferred written language: church Slavic | European | Aurum |
| 5838581000006115 | Afro-asiatic language | Other/Non-specific languages | Aurum |
| 1854331000006119 | preferred written language: Italian | European | Aurum |
| 312731000000113 | interpreter needed - Makaton sign language | UK languages | Aurum |
| 1854251000006115 | preferred written language: German | European | Aurum |
| 1706291000006110 | main spoken language Latin | European | Aurum |
| 5840811000006114 | Danish language | European | Aurum |
| 1778721000006112 | preferred communication language: Punjabi | South Asian | Aurum |
| 681931000000111 | Fijian language interpreter needed | Australasia | Aurum |
| 2615731000000114 | main spoken language Romany | European | Aurum |
| 8064201000006118 | Panjabi language interpreter needed | South Asian | Aurum |
| 6763581000006115 | main spoken language Ganda | Africa | Aurum |
| 1778311000006117 | preferred communication language: Akan (Ashanti) | Africa | Aurum |
| 1854291000006114 | preferred written language: Hausa | Africa | Aurum |
| 251241019 | language Gujarati | South Asian | Aurum |
| 8355381000006113 | Romanian as a second language | European | Aurum |
| 310211000000113 | Turkish as a second language | West & Central Asia | Aurum |
| 5840431000006117 | Kurukh language | South Asian | Aurum |
| 1706081000006117 | main spoken language church Slavic | European | Aurum |
| 1861781000006110 | preferred written language: aragonite | European | Aurum |
| 677771000000110 | Kirgiz language interpreter needed | West & Central Asia | Aurum |
| 683471000000119 | Aymara language interpreter needed | Americas | Aurum |
| 5845611000006116 | Thai language | East Asian | Aurum |
| 1863191000006113 | preferred written language: Xhosa | Africa | Aurum |
| 460083013 | main spoken language Arabic | West & Central Asia | Aurum |
| 671811000000118 | main spoken language Greenlandic | European | Aurum |
| 309221000000113 | Dutch as a second language | European | Aurum |
| 683531000000115 | Afrikaans language interpreter needed | Africa | Aurum |
| 1862191000006116 | preferred written language: Frisian | European | Aurum |
| 673371000000115 | main spoken language Moldavian | European | Aurum |
| 682581000000114 | Breton language interpreter needed | European | Aurum |
| 8063811000006118 | Japanese language interpreter needed | East Asian | Aurum |
| 1854281000006111 | preferred written language: Hakka | East Asian | Aurum |
| 2160070017 | main spoken language Flemish | European | Aurum |
| 2159259012 | main spoken language Ukrainian | European | Aurum |
| 1863231000006115 | preferred written language: Abkhazian | European | Aurum |
| 993201000006116 | language of interpreter needed | English/Unknown | Aurum |
| 1862881000006111 | preferred written language: Samoan | Australasia | Aurum |
| 1854531000006113 | preferred written language: Swedish | European | Aurum |
| 1706101000006113 | main spoken language Cornish | UK languages | Aurum |
| 673911000000114 | main spoken language Samoan | Australasia | Aurum |
| 1862651000006116 | preferred written language: Marathi | South Asian | Aurum |
| 1778481000006115 | preferred communication language: French creole | Americas | Aurum |
| 671231000000110 | main spoken language Danish | European | Aurum |
| 2643231000000111 | Romanes language interpreter needed | European | Aurum |
| 684771000000112 | Tibetan language interpreter needed | South Asian | Aurum |
| 678391000000112 | Malay language interpreter needed | East Asian | Aurum |
| 682111000000113 | Estonian language interpreter needed | European | Aurum |
| 8064461000006116 | Tagalog language interpreter needed | East Asian | Aurum |
| 2112371000000116 | main spoken language Aragonese | European | Aurum |
| 1854571000006111 | preferred written language: Thai | East Asian | Aurum |
| 460084019 | main spoken language Bengali | South Asian | Aurum |
| 684821000000118 | Punjabi language interpreter needed | South Asian | Aurum |
| 5841991000006119 | Madurese language | East Asian | Aurum |
| 5840911000006119 | Flemish language | European | Aurum |
| 8113251000006110 | Ndebele language interpreter needed | Africa | Aurum |
| 1863141000006116 | preferred written language: Venda | Africa | Aurum |
| 5845731000006118 | Lahu language | East Asian | Aurum |
| 7584761000006117 | Makaton language | UK languages | Aurum |
| 678011000000118 | Macedonian language interpreter needed | European | Aurum |
| 5841491000006113 | Oriya language | South Asian | Aurum |
| 1599281000000110 | main spoken language Nyanja | Africa | Aurum |
| 5841031000006117 | Catalan language | European | Aurum |
| 1778711000006116 | preferred communication language: Portuguese | European | Aurum |
| 676371000000112 | main spoken language Serbo-Croatian | European | Aurum |
| 674691000000112 | main spoken language Telugu | South Asian | Aurum |
| 1861881000006117 | preferred written language: Basque | European | Aurum |
| 8063781000006115 | Hindi language interpreter needed | South Asian | Aurum |
| 8064501000006116 | Tamil language interpreter needed | South Asian | Aurum |
| 8113231000006115 | Slovak language interpreter needed | European | Aurum |
| 5843321000006116 | Zulu language | Africa | Aurum |
| 1778451000006111 | preferred communication language: Finnish | European | Aurum |
| 8064551000006117 | Tigrinya language interpreter needed | Africa | Aurum |
| 1861921000006113 | preferred written language: Bokmal, Norwegian | European | Aurum |
| 1706441000006117 | main spoken language Pushto | West & Central Asia | Aurum |
| 992771000006111 | Punjabi as a second language | South Asian | Aurum |
| 1706521000006111 | using American sign language | Sign language | Aurum |
| 5839671000006112 | Dargin language | European | Aurum |
| 1862181000006119 | preferred written language: Filipino | East Asian | Aurum |
| 8228301000006119 | main spoken language Fulani | Africa | Aurum |
| 1854091000006118 | preferred written language: Amharic | Africa | Aurum |
| 5840671000006116 | Latvian language | European | Aurum |
| 1725911000006111 | main spoken language bemoan | Africa | Aurum |
| 1863151000006119 | preferred written language: Volapuk |  | Aurum |
| 1862291000006113 | preferred written language: Iba | South Asian | Aurum |
| 8064791000006115 | British sign language interpreter needed | UK languages | Aurum |
| 1862221000006111 | preferred written language: Georgian | European | Aurum |
| 1778661000006110 | preferred communication language: Mandarin | East Asian | Aurum |
| 1854501000006117 | preferred written language: Somali | Africa | Aurum |
| 5845331000006117 | Kanuri language | Africa | Aurum |
| 1862501000006116 | preferred written language: Kanyama | Africa | Aurum |
| 5845721000006116 | Karen language | East Asian | Aurum |
| 671751000000110 | main spoken language Georgian | European | Aurum |
| 2160065012 | main spoken language Ethiopian | Africa | Aurum |
| 8063701000006112 | Gujarati language interpreter needed | South Asian | Aurum |
| 1862871000006113 | preferred written language: Rundi | Africa | Aurum |
| 1862441000006115 | preferred written language: kikuyu | Africa | Aurum |
| 1861911000006117 | preferred written language: Bislama | Australasia | Aurum |
| 1862081000006115 | preferred written language: Czech | European | Aurum |
| 673491000000113 | main spoken language Nauru | Australasia | Aurum |
| 8354721000006114 | Hungarian as a second language | European | Aurum |
| 1862731000006119 | preferred written language: Ndebele, south | Africa | Aurum |
| 1862381000006117 | preferred written language: Javanese | East Asian | Aurum |
| 681871000000117 | Faroese language interpreter needed | European | Aurum |
| 460089012 | main spoken language Gujarati | South Asian | Aurum |
| 8063571000006112 | French language interpreter needed | European | Aurum |
| 1854211000006116 | preferred written language: Flemish | European | Aurum |
| 460102019 | main spoken language Spanish | European | Aurum |
| 1778691000006119 | preferred communication language: Patois | Americas | Aurum |
| 685121000000110 | Twi language interpreter needed | Africa | Aurum |
| 1778541000006110 | preferred communication language: Hausa | Africa | Aurum |
| 5845901000006112 | Finnish language | European | Aurum |
| 1757941000006116 | main spoken language nos | English/Unknown | Aurum |
| 993451000006116 | language of interpreter - Flemish | European | Aurum |
| 676391000000111 | main spoken language Serbo-Croatian | European | Aurum |
| 1706261000006119 | main spoken language Komi | European | Aurum |
| 8064531000006112 | Thai language interpreter needed | East Asian | Aurum |
| 5841461000006117 | Maldivian language | South Asian | Aurum |
| 6029861000006115 | Creole language | Americas | Aurum |
| 673791000000115 | main spoken language Quechua | Americas | Aurum |
| 8064141000006113 | Portuguese language interpreter needed | European | Aurum |
| 1854411000006115 | preferred written language: Norwegian | European | Aurum |
| 5838731000006114 | Oromo language | Africa | Aurum |
| 8064231000006114 | Russian language interpreter needed | European | Aurum |
| 670041000000119 | main spoken language afar | Africa | Aurum |
| 1706331000006115 | main spoken language Marshallese | Australasia | Aurum |
| 1863051000006118 | preferred written language: Tibetan | South Asian | Aurum |
| 1854391000006115 | preferred written language: Malayalam | South Asian | Aurum |
| 672171000000113 | main spoken language interlingua |  | Aurum |
| 1706491000006114 | main spoken language Venda | Africa | Aurum |
| 677651000000110 | Ethiopian language interpreter needed | Africa | Aurum |
| 678511000000111 | Kazakh language interpreter needed | West & Central Asia | Aurum |
| 460106016 | main spoken language Urdu | South Asian | Aurum |
| 1862591000006111 | preferred written language: Macedonian | European | Aurum |
| 5843901000006113 | Wolof language | Africa | Aurum |
| 675111000000112 | main spoken language Uzbek | West & Central Asia | Aurum |
| 2160076011 | main spoken language Lingala | Africa | Aurum |
| 682311000000110 | Kalaallit language interpreter needed | European | Aurum |
| 7302451000006118 | request for language interpreter service | English/Unknown | Aurum |
| 1706041000006111 | main spoken language Bosnian | European | Aurum |
| 5843691000006110 | Twi language | Africa | Aurum |
| 1862901000006113 | preferred written language: Sanskrit | South Asian | Aurum |
| 4538941000006114 | Punjabi language | South Asian | Aurum |
| 677891000000118 | Māori language interpreter needed | Australasia | Aurum |
| 678451000000111 | Kashmiri language interpreter needed | South Asian | Aurum |
| 8064331000006118 | Somali language interpreter needed | Africa | Aurum |
| 460095013 | main spoken language Kutchi | South Asian | Aurum |
| 460087014 | main spoken language English | English/Unknown | Aurum |
| 8110701000006114 | English language interpreter needed | English/Unknown | Aurum |
| 5838691000006118 | Hausa language | Africa | Aurum |
| 1599291000000112 | main spoken language Chewa | Africa | Aurum |
| 5843561000006115 | Ewe language | Africa | Aurum |
| 8063611000006119 | French creole language interpreter needed | Americas | Aurum |
| 1706151000006112 | main spoken language western Frisian | European | Aurum |
| 1863001000006117 | preferred written language: Tahitian | Australasia | Aurum |
| 1706391000006116 | main spoken language Norwegian Bokmal | European | Aurum |
| 1778701000006119 | preferred communication language: polish | European | Aurum |
| 2160057014 | main spoken language Amharic | Africa | Aurum |
| 1862921000006115 | preferred written language: Shona | Africa | Aurum |
| 1862491000006112 | preferred written language: Konkani | South Asian | Aurum |
| 670581000000118 | main spoken language Dzongkha | South Asian | Aurum |
| 1862711000006113 | preferred written language: Ndebele | Africa | Aurum |
| 5843241000006116 | Tswana language | Africa | Aurum |
| 5839241000006119 | Balkar language | European | Aurum |
| 5840881000006119 | Afrikaans language | Africa | Aurum |
| 2615761000000116 | main spoken language Romsky | European | Aurum |
| 1863111000006115 | preferred written language: Uighur | East Asian | Aurum |
| 309981000000118 | Ukrainian as a second language | European | Aurum |
| 1854481000006110 | preferred written language: Serbian/Croatian | European | Aurum |
| 674571000000112 | main spoken language Tajik | West & Central Asia | Aurum |
| 309551000000117 | Russian as a second language | European | Aurum |
| 8354541000006112 | Danish as a second language | European | Aurum |
| 1862141000006113 | preferred written language: Estonian | European | Aurum |
| 5843141000006113 | Shona language | Africa | Aurum |
| 1778431000006116 | preferred communication language: Ethiopian | Africa | Aurum |
| 1854121000006119 | preferred written language: Brawa & Somali | Africa | Aurum |
| 1854141000006114 | preferred written language: Cantonese and Vietnamese | East Asian | Aurum |
| 2160079016 | main spoken language Malayalam | South Asian | Aurum |
| 1863081000006114 | preferred written language: Tswana | Africa | Aurum |
| 5841321000006117 | Slovak language | European | Aurum |
| 5840691000006115 | Lithuanian language | European | Aurum |
| 2160127015 | using Makaton sign language | UK languages | Aurum |
| 4930471000006116 | Hinko language | South Asian | Aurum |
| 2160078012 | main spoken language Dutch | European | Aurum |
| 1706161000006114 | main spoken language Fulah | Africa | Aurum |
| 678811000000114 | Lao language interpreter needed | East Asian | Aurum |
| 683001000000115 | central Khmer language interpreter needed | East Asian | Aurum |
| 1862261000006117 | preferred written language: Hindko | South Asian | Aurum |
| 312701000000119 | interpreter needed - British sign language | UK languages | Aurum |
| 251244010 | language Hindi | South Asian | Aurum |
| 2533545014 | main spoken language Finnish | European | Aurum |
| 1778371000006114 | preferred communication language: British signing language | UK languages | Aurum |
| 671171000000117 | main spoken language Corsican | European | Aurum |
| 5843331000006118 | Efik language | Africa | Aurum |
| 1862361000006110 | preferred written language: Inupiaq | Americas | Aurum |
| 1677951000006111 | supplemental main language spoken | English/Unknown | Aurum |
| 8063741000006114 | Hausa language interpreter needed | Africa | Aurum |
| 1778421000006119 | preferred communication language: English | English/Unknown | Aurum |
| 5838771000006112 | Somali language | Africa | Aurum |
| 4538851000006119 | Gujarati language | South Asian | Aurum |
| 671031000000118 | main spoken language Catalan | European | Aurum |
| 5838901000006115 | Amharic language | Africa | Aurum |
| 310361000000118 | Welsh as a second language | UK languages | Aurum |
| 2638711000000115 | Romany language interpreter needed | European | Aurum |
| 5845701000006114 | Kachin language | East Asian | Aurum |
| 642141000000118 | main spoken language Bulgarian | European | Aurum |
| 685651000000113 | Turkmen language interpreter needed | West & Central Asia | Aurum |
| 309691000000114 | Tamil as a second language | South Asian | Aurum |
| 671931000000117 | main spoken language Hungarian | European | Aurum |
| 5839591000006114 | Australian language | Australasia | Aurum |
| 1778321000006113 | preferred communication language: Albanian | European | Aurum |
| 674271000000110 | main spoken language Sindhi | South Asian | Aurum |
| 672231000000115 | main spoken language Inupiaq | Americas | Aurum |
| 1706371000006117 | main spoken language Ndonga | Africa | Aurum |
| 5840851000006110 | Norwegian language | European | Aurum |
| 5839021000006117 | Mongolian language | East Asian | Aurum |
| 681991000000112 | Galician language interpreter needed | European | Aurum |
| 1706021000006116 | main spoken language Avestan | West & Central Asia | Aurum |
| 5840081000006110 | central Sudanic language | Africa | Aurum |
| 1862761000006111 | preferred written language: northern Sami | European | Aurum |
| 1658371000000110 | main spoken language Konkani | South Asian | Aurum |
| 671411000000115 | main spoken language Faroese | European | Aurum |
| 5839471000006114 | Turkish language | West & Central Asia | Aurum |
| 1862281000006110 | preferred written language: Hungarian | European | Aurum |
| 672711000000119 | main spoken language Kirghiz | West & Central Asia | Aurum |
| 678211000000113 | Latvian language interpreter needed | European | Aurum |
| 5843341000006111 | Ibibio language | Africa | Aurum |
| 1706071000006115 | main spoken language Chinese | East Asian | Aurum |
| 1778791000006114 | preferred communication language: Swedish | European | Aurum |
| 1862011000006110 | preferred written language: Chichewa | Africa | Aurum |
| 673071000000117 | main spoken language Malagasy | Africa | Aurum |
| 1778881000006118 | preferred communication language: Welsh | UK languages | Aurum |
| 993431000006111 | language of interpreter - Ethiopian | Africa | Aurum |
| 682821000000110 | Armenian language interpreter needed | West & Central Asia | Aurum |
| 1778461000006113 | preferred communication language: Flemish | European | Aurum |
| 5840651000006114 | Armenian language | West & Central Asia | Aurum |
| 5840661000006111 | Baltic language | European | Aurum |
| 2638731000000111 | Romani language interpreter needed | European | Aurum |
| 1706111000006111 | main spoken language Cree | Americas | Aurum |
| 1854381000006118 | preferred written language: Luganda | Africa | Aurum |
| 309101000000113 | Amharic as a second language | Africa | Aurum |
| 460096014 | main spoken language mandarin | East Asian | Aurum |
| 685731000000111 | Slovenian language interpreter needed | European | Aurum |
| 676151000000117 | main spoken language Kalaallisut | European | Aurum |
| 1778601000006114 | preferred communication language: Korean | East Asian | Aurum |
| 5838861000006110 | Hebrew language | West & Central Asia | Aurum |
| 678931000000110 | Hungarian language interpreter needed | European | Aurum |
| 1862641000006118 | preferred written language: Māori | Australasia | Aurum |
| 309261000000117 | Irish Gaelic as a second language | UK languages | Aurum |
| 8355321000006114 | Panjabi as a second language | South Asian | Aurum |
| 1854421000006111 | preferred written language: Pashto (Pushtoo) | West & Central Asia | Aurum |
| 5845601000006119 | Shan language | East Asian | Aurum |
| 5842001000006117 | Malagasy language | Africa | Aurum |
| 5839481000006112 | Turkmen language | West & Central Asia | Aurum |
| 1862911000006111 | preferred written language: Sardinian | European | Aurum |
| 1862091000006117 | preferred written language: Danish | European | Aurum |
| 1862511000006118 | preferred written language: Kutchi | South Asian | Aurum |
| 251251018 | language Punjabi | South Asian | Aurum |
| 2339911000000119 | first language not English | English/Unknown | Aurum |
| 1861801000006114 | preferred written language: Assamese | South Asian | Aurum |
| 5840591000006119 | Japanese language | East Asian | Aurum |
| 1862321000006116 | preferred written language: Indonesian | East Asian | Aurum |
| 1862471000006111 | preferred written language: Komi | European | Aurum |
| 1778361000006119 | preferred communication language: Brawa & Somali | Africa | Aurum |
| 5841621000006114 | Sindhi language | South Asian | Aurum |
| 460107013 | main spoken language Yoruba | Africa | Aurum |
| 5840731000006111 | Welsh language | UK languages | Aurum |
| 675471000000110 | main spoken language Zulu | Africa | Aurum |
| 683621000000116 | Azerbaijani language interpreter needed | European | Aurum |
| 678091000000110 | Malagasy language interpreter needed | Africa | Aurum |
| 1862481000006114 | preferred written language: Kongo | Africa | Aurum |
| 309061000000111 | Akan as a second language | Africa | Aurum |
| 1854561000006116 | preferred written language: Tamil | South Asian | Aurum |
| 1862171000006117 | preferred written language: Fijian | Australasia | Aurum |
| 1712821000006110 | main spoken language Konkani | South Asian | Aurum |
| 5841171000006118 | Slavic language | European | Aurum |
| 1854431000006114 | preferred written language: Patois | Americas | Aurum |
| 5840451000006112 | Malayalam language | South Asian | Aurum |
| 251240018 | language Bengali | South Asian | Aurum |
| 5841381000006118 | Assamese language | South Asian | Aurum |
| 309161000000112 | Chinese as a second language | East Asian | Aurum |
| 5841261000006118 | Macedonian language | European | Aurum |
| 1778521000006115 | preferred communication language: Gujarati | South Asian | Aurum |
| 8354261000006114 | Afrikaans as a second language | Africa | Aurum |
| 309631000000113 | Swahili as a second language | Africa | Aurum |
| 1854621000006114 | preferred written language: Welsh | UK languages | Aurum |
| 678871000000116 | Icelandic language interpreter needed | European | Aurum |
| 1862821000006112 | preferred written language: Ossetian | European | Aurum |
| 5842391000006110 | Khasi language | South Asian | Aurum |
| 1854111000006110 | preferred written language: Bengali & Sylheti | South Asian | Aurum |
| 1706061000006110 | main spoken language Chechen | European | Aurum |
| 1863031000006113 | preferred written language: Telugu | South Asian | Aurum |
| 1861981000006112 | preferred written language: central Khmer | East Asian | Aurum |
| 1706421000006112 | main spoken language Ossetian | European | Aurum |
| 8354421000006116 | Bulgarian as a second language | European | Aurum |
| 684061000000119 | Zulu language interpreter needed | Africa | Aurum |
| 5843601000006115 | Ga language | Africa | Aurum |
| 1863181000006110 | preferred written language: Wolof | Africa | Aurum |
| 2160066013 | main spoken language Swedish | European | Aurum |
| 674631000000111 | main spoken language Tatar | European | Aurum |
| 672951000000118 | main spoken language Latvian | European | Aurum |
| 8064371000006115 | Swahili language interpreter needed | Africa | Aurum |
| 683681000000115 | Basque language interpreter needed | European | Aurum |
| 2160080018 | main spoken language Norwegian | European | Aurum |
| 673431000000112 | main spoken language Mongolian | East Asian | Aurum |
| 993821000006110 | sign interpreter needed - British sign language | UK languages | Aurum |
| 684691000000116 | Serbo-Croatian language interpreter needed | European | Aurum |
| 216247015 | using British sign language | UK languages | Aurum |
| 1706341000006113 | main spoken language Navajo | Americas | Aurum |
| 672351000000119 | main spoken language Irish | UK languages | Aurum |
| 437977019 | Vietnamese language | East Asian | Aurum |
| 5843551000006117 | Edo language | Africa | Aurum |
| 2160072013 | main spoken language Gaelic | UK languages | Aurum |
| 5843731000006119 | Bambara language | Africa | Aurum |
| 2643221000000114 | Romanes as a second language | European | Aurum |
| 2159261015 | main spoken language Vietnamese | East Asian | Aurum |
| 1778641000006111 | preferred communication language: Makaton (sign language) | UK languages | Aurum |
| 1854581000006114 | preferred written language: Tigrinya | Africa | Aurum |
| 1862311000006112 | preferred written language: Ido |  | Aurum |
| 8355191000006114 | Nepali as a second language | South Asian | Aurum |
| 2159242019 | main spoken language Italian | European | Aurum |
| 678631000000119 | Javanese language interpreter needed | East Asian | Aurum |
| 8063901000006110 | Lithuanian language interpreter needed | European | Aurum |
| 5838831000006118 | Assyrian language | West & Central Asia | Aurum |
| 1863091000006112 | preferred written language: Turkmen | West & Central Asia | Aurum |
| 8063431000006110 | Italian language interpreter needed | European | Aurum |
| 2160068014 | main spoken language Sinhala | South Asian | Aurum |
| 5841691000006111 | Kurdish language | West & Central Asia | Aurum |
| 686991000000118 | Oriya language interpreter needed | South Asian | Aurum |
| 1861861000006110 | preferred written language: Bamun | Africa | Aurum |
| 1118721000000116 | main spoken language Filipino | East Asian | Aurum |
| 2160069018 | main spoken language Thai | East Asian | Aurum |
| 1778781000006111 | preferred communication language: Swahili | Africa | Aurum |
| 460098010 | main spoken language Portuguese | European | Aurum |
| 692821000000111 | Greenlandic language interpreter needed | European | Aurum |
| 5841071000006119 | Italian language | European | Aurum |
| 5842401000006112 | Khmer language | East Asian | Aurum |
| 1861821000006116 | preferred written language: Avestan | West & Central Asia | Aurum |
| 5844681000006110 | Tanoan language | Americas | Aurum |
| 8064301000006114 | Sinhala language interpreter needed | South Asian | Aurum |
| 1854491000006113 | preferred written language: Sinhala | South Asian | Aurum |
| 8063521000006111 | Dutch language interpreter needed | European | Aurum |
| 2615751000000119 | main spoken language Romani | European | Aurum |
| 5840861000006112 | Swedish language | European | Aurum |
| 8355081000006110 | Maltese as a second language | European | Aurum |
| 5841131000006116 | Rumanian language | European | Aurum |
| 1863211000006114 | preferred written language: Zhuang | East Asian | Aurum |
| 1862111000006114 | preferred written language: Divehi | South Asian | Aurum |
| 1862661000006119 | preferred written language: Marshallese | Australasia | Aurum |
| 8064681000006112 | Yoruba language interpreter needed | Africa | Aurum |
| 5841041000006110 | French language | European | Aurum |
| 5841011000006111 | Latin language | European | Aurum |
| 1706381000006119 | main spoken language Norwegian Nynorsk | European | Aurum |
| 1854181000006115 | preferred written language: Ethiopian | Africa | Aurum |
| 664911000000115 | Romanian language interpreter needed | European | Aurum |
| 457312017 | Mirpuri language | South Asian | Aurum |
| 8063501000006118 | Czech language interpreter needed | European | Aurum |
| 5841431000006114 | Hindustani language | South Asian | Aurum |
| 1778591000006118 | preferred communication language: Japanese | East Asian | Aurum |
| 1862961000006114 | preferred written language: Slovenian | European | Aurum |
| 2615771000000111 | main spoken language Romanesa | European | Aurum |
| 8063321000006118 | Amharic language interpreter needed | Africa | Aurum |
| 5838991000006110 | south Arabic language | West & Central Asia | Aurum |
| 1677341000006110 | additional main spoken language | English/Unknown | Aurum |
| 992901000006119 | Hakka as a second language | East Asian | Aurum |
| 5841761000006112 | Bushman language | Africa | Aurum |
| 1778671000006115 | preferred communication language: Norwegian | European | Aurum |
| 5840941000006115 | German language | European | Aurum |
| 670521000000119 | main spoken language Basque | European | Aurum |
| 1862621000006113 | preferred written language: Maltese | European | Aurum |
| 1706231000006111 | main spoken language Sichuan Yi | East Asian | Aurum |
| 8064281000006110 | Shona language interpreter needed | Africa | Aurum |
| 5840781000006112 | Germanic language | European | Aurum |
| 309371000000119 | Hindi as a second language | South Asian | Aurum |
| 1862241000006116 | preferred written language: Haitian | Americas | Aurum |
| 1862941000006110 | preferred written language: Sindhi | South Asian | Aurum |
| 1778831000006119 | preferred communication language: Thai | East Asian | Aurum |
| 675291000000113 | main spoken language Xhosa | Africa | Aurum |
| 5843361000006110 | Ijo language | Africa | Aurum |
| 309471000000113 | Lithuanian as a second language | European | Aurum |
| 2159257014 | main spoken language Turkish | West & Central Asia | Aurum |
| 992841000006110 | Flemish as a second language | European | Aurum |
| 309451000000116 | Kurdish as a second language | West & Central Asia | Aurum |
| 1778561000006114 | preferred communication language: Hindi | South Asian | Aurum |
| 1706311000006114 | main spoken language Luxembourgish | European | Aurum |
| 1706511000006115 | main spoken language Walloon | European | Aurum |
| 5841301000006110 | Czech language | European | Aurum |
| 5838791000006113 | Egyptian language | Africa | Aurum |
| 8063681000006114 | Greek language interpreter needed | European | Aurum |
| 992681000006115 | Iba as a second language | South Asian | Aurum |
| 1854641000006119 | preferred written language: other | English/Unknown | Aurum |
| 5842011000006119 | Malay language | East Asian | Aurum |
| 5840951000006118 | Luxembourgian language | European | Aurum |
| 677531000000116 | Maltese language interpreter needed | European | Aurum |
| 670971000000114 | main spoken language central Khmer | East Asian | Aurum |
| 1778471000006118 | preferred communication language: French | European | Aurum |
| 8064641000006118 | Vietnamese language interpreter needed | East Asian | Aurum |
| 2160077019 | main spoken language Luganda | Africa | Aurum |
| 1778331000006111 | preferred communication language: Amharic | Africa | Aurum |
| 1862671000006114 | preferred written language: Moldavian | European | Aurum |
| 8064351000006113 | Spanish language interpreter needed | European | Aurum |
| 1862451000006118 | preferred written language: Kinyarwanda | Africa | Aurum |
| 5840101000006119 | Madi language | Africa | Aurum |
| 1862841000006117 | preferred written language: Quechua | Americas | Aurum |
| 685201000000110 | Telugu language interpreter needed | South Asian | Aurum |
| 5842791000006114 | Herero language | Africa | Aurum |
| 1706271000006114 | main spoken language Kongo | Africa | Aurum |
| 5838951000006116 | Tigrinya language | Africa | Aurum |
| 1706361000006112 | main spoken language northern Ndebele | Africa | Aurum |
| 5845531000006111 | Tai language | East Asian | Aurum |
| 5838981000006112 | classical Arabic language | West & Central Asia | Aurum |
| 1706241000006118 | main spoken language Kanuri | Africa | Aurum |
| 992781000006114 | Brawa as a second language | Africa | Aurum |
| 2160067016 | main spoken language Tagalog | East Asian | Aurum |
| 309651000000118 | Swedish as a second language | European | Aurum |
| 2159243012 | main spoken language German | European | Aurum |
| 5841221000006112 | Russian language | European | Aurum |
| 4538921000006119 | Pashto language | West & Central Asia | Aurum |
| 309201000000116 | Czech as a second language | European | Aurum |
| 1854341000006112 | preferred written language: Japanese | East Asian | Aurum |
| 1863121000006111 | preferred written language: Ukrainian | European | Aurum |
| 673611000000118 | main spoken language Occitan | European | Aurum |
| 672291000000119 | main spoken language Inuktitut | Americas | Aurum |
| 1778551000006112 | preferred communication language: Hebrew | West & Central Asia | Aurum |
| 5841361000006111 | Indo-Iranian language | Other/Non-specific languages | Aurum |
| 460100010 | main spoken language Russian | European | Aurum |
| 670171000000112 | main spoken language Armenian | West & Central Asia | Aurum |
| 309591000000113 | Somali as a second language | Africa | Aurum |
| 682521000000113 | Indonesian language interpreter needed | East Asian | Aurum |
| 8063451000006115 | Cantonese language interpreter needed | East Asian | Aurum |
| 460104018 | main spoken language Sylheti | South Asian | Aurum |
| 5841721000006118 | Persian language | West & Central Asia | Aurum |
| 684751000000115 | Serbo-Croatian language interpreter needed | European | Aurum |
| 669921000000113 | main spoken language Oromo | Africa | Aurum |
| 1862701000006110 | preferred written language: Navajo | Americas | Aurum |
| 1778771000006113 | preferred communication language: Spanish | European | Aurum |
| 1862201000006118 | preferred written language: Fulah | Africa | Aurum |
| 5843171000006117 | Swahili language | Africa | Aurum |
| 8355351000006117 | Pushto as a second language | West & Central Asia | Aurum |
| 1862051000006111 | preferred written language: Cornish | UK languages | Aurum |
| 8064661000006119 | Welsh language interpreter needed | UK languages | Aurum |
| 5838841000006111 | Syriac language | West & Central Asia | Aurum |
| 5842051000006118 | Pampanga language | East Asian | Aurum |
| 1854461000006117 | preferred written language: Punjabi | South Asian | Aurum |
| 2160081019 | main spoken language Pashto | West & Central Asia | Aurum |
| 5842811000006113 | Kikuyu language | Africa | Aurum |
| 1706211000006117 | main spoken language Hiri Motu | Australasia | Aurum |
| 1862851000006115 | preferred written language: Romanian | European | Aurum |
| 5838941000006118 | Tigre language | Africa | Aurum |
| 1862771000006116 | preferred written language: Norwegian Nynorsk | European | Aurum |
| 5838971000006114 | Arabic language | West & Central Asia | Aurum |
| 5840641000006112 | Albanian language | European | Aurum |
| 5845651000006115 | Burmese language | East Asian | Aurum |
| 677831000000119 | Iban language interpreter needed | South Asian | Aurum |
| 5842551000006112 | Niger-Congo language | Africa | Aurum |
| 5843381000006117 | western Sudanic language | Africa | Aurum |
| 2638741000000119 | Romsky language interpreter needed | European | Aurum |
| 1861771000006112 | preferred written language: Afrikaans | Africa | Aurum |
| 1862561000006115 | preferred written language: Lithuanian | European | Aurum |
| 672651000000112 | main spoken language Kinyarwanda | Africa | Aurum |
| 5840271000006118 | western Nilotic language | Africa | Aurum |
| 1854361000006111 | preferred written language: Kurdish | West & Central Asia | Aurum |
| 1888671000006114 | English not main spoken language at home | English/Unknown | Aurum |
| 1863161000006117 | preferred written language: Walloon | European | Aurum |
| 1862131000006115 | preferred written language: Esperanto |  | Aurum |
| 1861951000006116 | preferred written language: Bulgarian | European | Aurum |
| 5844881000006114 | Kwakiutl language | Americas | Aurum |
| 5840821000006118 | Faroese language | European | Aurum |
| 1778441000006114 | preferred communication language: Farsi (Persian) | West & Central Asia | Aurum |
| 5841001000006113 | Italic language | Other/Non-specific languages | Aurum |
| 1863041000006115 | preferred written language: Tetum | East Asian | Aurum |
| 310151000000118 | Norwegian as a second language | European | Aurum |
| 671291000000111 | main spoken language Esperanto |  | Aurum |
| 992981000006111 | Luganda as a second language | Africa | Aurum |
| 1862931000006117 | preferred written language: Sichuan Yi | East Asian | Aurum |
| 1862421000006110 | preferred written language: Kashmiri | South Asian | Aurum |
| 8230881000006118 | Dari as a second language | West & Central Asia | Aurum |
| 5838601000006113 | Berber language | Africa | Aurum |
| 5842851000006114 | Lingala language | Africa | Aurum |
| 5840891000006116 | Dutch language | European | Aurum |
| 992861000006114 | French creole as a second language | Americas | Aurum |
| 5838651000006112 | Tamazight language | Africa | Aurum |
| 1862151000006110 | preferred written language: Ewe | Africa | Aurum |
| 5842751000006115 | Ganda language | Africa | Aurum |
| 5841441000006116 | Kashmiri language | South Asian | Aurum |
| 662801000000116 | main spoken language Slovak | European | Aurum |
| 1861831000006118 | preferred written language: Aymara | Americas | Aurum |
| 678571000000118 | Inupiaq language interpreter needed | Americas | Aurum |
| 1862971000006119 | preferred written language: southern Sotho | Africa | Aurum |
| 677471000000115 | Marathi language interpreter needed | South Asian | Aurum |
| 1862331000006118 | preferred written language: interlingua |  | Aurum |
| 992821000006115 | Ethiopian as a second language | Africa | Aurum |
| 1862791000006115 | preferred written language: Ojibwa | Americas | Aurum |
| 1862571000006110 | preferred written language: Luba Katanga | Africa | Aurum |
| 1854261000006118 | preferred written language: Greek | European | Aurum |
| 5846131000006113 | language commonly spoken in Europe | European | Aurum |
| 5841661000006115 | Iranian language | West & Central Asia | Aurum |
| 4538911000006110 | Pushto language | West & Central Asia | Aurum |
| 5842731000006110 | Duala language | Africa | Aurum |
| 662861000000117 | main spoken language Ndebele | Africa | Aurum |
| 993391000006117 | language of interpreter - Brawa | Africa | Aurum |
| 1706451000006115 | main spoken language Sanskrit | South Asian | Aurum |
| 5839121000006116 | Manchu language | East Asian | Aurum |
| 993671000006112 | language of interpreter - Patois | Americas | Aurum |
| 645201000006112 | English as a second language | English/Unknown | Aurum |
| 683791000000113 | afar language interpreter needed | Africa | Aurum |
| 673671000000111 | main spoken language Oriya | South Asian | Aurum |
| 993591000006114 | language of interpreter - Luganda | Africa | Aurum |
| 1862531000006112 | preferred written language: Latin | European | Aurum |
| 1862101000006111 | preferred written language: Dari | West & Central Asia | Aurum |
| 309501000000118 | Polish as a second language | European | Aurum |
| 1862691000006110 | preferred written language: Nauru | Australasia | Aurum |
| 1778381000006112 | preferred communication language: Cantonese | East Asian | Aurum |
| 1778681000006117 | preferred communication language: Pashto (Pushtoo) | West & Central Asia | Aurum |
| 5845131000006119 | Enga language | Australasia | Aurum |
| 5840471000006119 | Telugu language | South Asian | Aurum |
| 1861971000006114 | preferred written language: Catalan | European | Aurum |
| 5842111000006115 | Visayan language | East Asian | Aurum |
| 5841591000006112 | Romany language | European | Aurum |
| 5841851000006116 | Malayo-Polynesian language | East Asian | Aurum |
| 5843191000006116 | Swazi language | Africa | Aurum |
| 1862541000006119 | preferred written language: Latvian | European | Aurum |
| 460094012 | main spoken language Iba | South Asian | Aurum |
| 677351000000117 | Mongolian language interpreter needed | East Asian | Aurum |
| 670311000000117 | main spoken language Aymara | Americas | Aurum |
| 5838821000006116 | Aramaic language | West & Central Asia | Aurum |
| 1854131000006116 | preferred written language: Cantonese | East Asian | Aurum |
| 670241000000111 | main spoken language Assamese | South Asian | Aurum |
| 1854311000006113 | preferred written language: Hindi | South Asian | Aurum |
| 1854221000006112 | preferred written language: French | European | Aurum |
| 1778391000006110 | preferred communication language: Cantonese and Vietnamese | East Asian | Aurum |
| 5841871000006114 | Indonesian language | East Asian | Aurum |
| 1778351000006116 | preferred communication language: Bengali & Sylheti | South Asian | Aurum |
| 460105017 | main spoken language Tamil | South Asian | Aurum |
| 992691000006117 | Kutchi as a second language | South Asian | Aurum |
| 5843721000006117 | Mande language | Africa | Aurum |
| 671691000000114 | main spoken language Galician | European | Aurum |
| 2615881000000116 | Romanesa as a second language | European | Aurum |
| 5839461000006119 | Azerbaijani language | European | Aurum |
| 1854521000006110 | preferred written language: Swahili | Africa | Aurum |
| 1706461000006118 | main spoken language northern Sami | European | Aurum |
| 2159256017 | main spoken language Lithuanian | European | Aurum |
| 675231000000112 | main spoken language Wolof | Africa | Aurum |
| 5845211000006119 | French Creole language | Americas | Aurum |
| 1862951000006112 | preferred written language: Slovak | European | Aurum |
| 1861871000006115 | preferred written language: Bashkir | European | Aurum |
| 673311000000113 | main spoken language Marathi | South Asian | Aurum |
| 8064571000006110 | Turkish language interpreter needed | West & Central Asia | Aurum |
| 1858911000006119 | preferred written language: not recorded | English/Unknown | Aurum |
| 5845881000006110 | Finnic language | European | Aurum |
| 682641000000119 | Bislama language interpreter needed | Australasia | Aurum |
| 1854101000006112 | preferred written language: Arabic | West & Central Asia | Aurum |
| 310801000000112 | Farsi language interpreter needed | West & Central Asia | Aurum |
| 1854321000006117 | preferred written language: Igbo (Ibo) | Africa | Aurum |
| 1778501000006113 | preferred communication language: German | European | Aurum |
| 1862271000006112 | preferred written language: Hiri Motu | Australasia | Aurum |
| 1861961000006119 | preferred written language: Burmese | East Asian | Aurum |
| 5841481000006110 | Nepali language | South Asian | Aurum |
| 1862041000006114 | preferred written language: Chuvash | European | Aurum |
| 2159255018 | main spoken language Korean | East Asian | Aurum |
| 1854271000006113 | preferred written language: Gujarati | South Asian | Aurum |
| 309331000000116 | Hausa as a second language | Africa | Aurum |
| 1488807012 | main spoken language Farsi | West & Central Asia | Aurum |
| 5843351000006113 | Tiv language | Africa | Aurum |
| 5843791000006115 | Soninke language | Africa | Aurum |
| 1706191000006118 | main spoken language Haitian | Americas | Aurum |
| 1861941000006118 | preferred written language: Breton | European | Aurum |
| 1488808019 | main spoken language Kurdish | West & Central Asia | Aurum |
| 5843701000006110 | Urhobo language | Africa | Aurum |
| 5841081000006116 | Moldavian language | European | Aurum |
| 1862681000006112 | preferred written language: Mongolian | East Asian | Aurum |
| 5843001000006112 | Ndebele language | Africa | Aurum |
| 5842591000006118 | eastern language (Niger-Congo) | Africa | Aurum |
| 5841561000006116 | Sikh Punjabi language | South Asian | Aurum |
| 1863021000006110 | preferred written language: Tatar | European | Aurum |
| 1728131000006113 | other main spoken language | English/Unknown | Aurum |
| 682371000000117 | Catalan language interpreter needed | European | Aurum |
| 5839751000006112 | Georgian language | European | Aurum |
| 686111000000112 | Sundanese language interpreter needed | English/Unknown | Aurum |
| 309311000000112 | Gujarati as a second language | South Asian | Aurum |
| 1778731000006110 | preferred communication language: Russian | European | Aurum |
| 682701000000112 | Bihari language interpreter needed | South Asian | Aurum |
| 309671000000110 | Tagalog as a second language | East Asian | Aurum |
| 310131000000113 | Malayalam as a second language | South Asian | Aurum |
| 1706321000006118 | main spoken language Luba Katanga | Africa | Aurum |
| 309961000000110 | German as a second language | European | Aurum |
| 7511041000006119 | main spoken language Abkhaz | European | Aurum |
| 671871000000111 | main spoken language Guarani | Americas | Aurum |
| 5839721000006115 | Chechen language | European | Aurum |
| 1862741000006112 | preferred written language: Ndonga | Africa | Aurum |
| 5843891000006114 | Temne language | Africa | Aurum |
| 5841021000006115 | romance language | European | Aurum |
| 5841191000006117 | Belarusian language | European | Aurum |
| 8063851000006117 | Kurdish language interpreter needed | West & Central Asia | Aurum |
| 686651000000115 | Sango language interpreter needed | Africa | Aurum |
| 1778621000006116 | preferred communication language: Lingala | Africa | Aurum |
| 687531000000112 | Romansh language interpreter needed | European | Aurum |
| 5840601000006110 | Korean language | East Asian | Aurum |
| 1862611000006117 | preferred written language: Malay | East Asian | Aurum |
| 686231000000112 | Samoan language interpreter needed | Australasia | Aurum |
| 5843531000006112 | Bassa language | Africa | Aurum |
| 1862351000006113 | preferred written language: Inuktitut | Americas | Aurum |
| 8064611000006117 | Urdu language interpreter needed | South Asian | Aurum |
| 684501000000112 | Yiddish language interpreter needed | European | Aurum |
| 675351000000113 | main spoken language Yiddish | European | Aurum |
| 686851000000116 | Uighur language interpreter needed | East Asian | Aurum |
| 670781000000113 | main spoken language Breton | European | Aurum |
| 2418501000000119 | main spoken language Gujarati | South Asian | Aurum |
| 5840841000006113 | Icelandic language | European | Aurum |
| 670101000000116 | main spoken language Afrikaans | Africa | Aurum |
| 683851000000113 | Abkhazian language interpreter needed | European | Aurum |
| 1706351000006110 | main spoken language south Ndebele | Africa | Aurum |
| 8354451000006113 | Catalan as a second language | European | Aurum |
| 1854451000006119 | preferred written language: Portuguese | European | Aurum |
| 5842661000006110 | Bemba language | Africa | Aurum |
| 1727721000006110 | main spoken language Tetum | East Asian | Aurum |
| 310191000000114 | Tigrinya as a second language | Africa | Aurum |
| 460086017 | main spoken language Czech | European | Aurum |
| 672771000000112 | main spoken language Rundi | Africa | Aurum |
| 675961000000112 | main spoken language Sinhalese | South Asian | Aurum |
| 1778901000006116 | preferred communication language: other | English/Unknown | Aurum |
| 1862071000006118 | preferred written language: Cree | Americas | Aurum |
| 670911000000116 | main spoken language Belarusian | European | Aurum |
| 5843051000006111 | Nyanja language | Africa | Aurum |
| 993091000006115 | Sinhala as a second language | South Asian | Aurum |
| 1138281000000117 | main spoken language Hindko | South Asian | Aurum |
| 1778811000006113 | preferred communication language: Tagalog (Filipino) | East Asian | Aurum |
| 1706221000006113 | main spoken language Ido |  | Aurum |
| 1778341000006118 | preferred communication language: Arabic | West & Central Asia | Aurum |
| 309611000000117 | Spanish as a second language | European | Aurum |
| 8063341000006113 | Arabic language interpreter needed | West & Central Asia | Aurum |
| 251248013 | language Pashtu | West & Central Asia | Aurum |
| 8354761000006115 | Indonesian as a second language | East Asian | Aurum |
| 673011000000110 | main spoken language Macedonian | European | Aurum |
| 684381000000113 | Uzbek language interpreter needed | West & Central Asia | Aurum |
| 1778891000006115 | preferred communication language: Yoruba | Africa | Aurum |
| 1861991000006110 | preferred written language: Chamorro | Australasia | Aurum |
| 8064021000006119 | Mandarin language interpreter needed | East Asian | Aurum |
| 1862721000006117 | preferred written language: Ndebele, north | Africa | Aurum |
| 5845891000006113 | Estonian language | European | Aurum |
| 460093018 | main spoken language Hindi | South Asian | Aurum |
| 674931000000115 | main spoken language Turkmen | West & Central Asia | Aurum |
| 672591000000117 | main spoken language Kazakh | West & Central Asia | Aurum |
| 4538871000006112 | Hindi language | South Asian | Aurum |
| 1778861000006111 | preferred communication language: Urdu | South Asian | Aurum |
| 1862161000006112 | preferred written language: Faroese | European | Aurum |
| 1862411000006119 | preferred written language: Kanuri | Africa | Aurum |
| 2615871000000118 | Romani as a second language | European | Aurum |
| 1778651000006113 | preferred communication language: Malayalam | South Asian | Aurum |
| 1854081000006116 | preferred written language: Albanian | European | Aurum |
| 1706431000006110 | main spoken language Pali | South Asian | Aurum |
| 8064811000006116 | Makaton sign language interpreter needed | UK languages | Aurum |
| 1862461000006116 | preferred written language: Kirgiz | West & Central Asia | Aurum |
| 7263671000006111 | sign language | UK languages | Aurum |
| 1166931000000118 | main spoken language Bamoun | Africa | Aurum |
| 8064391000006119 | Swedish language interpreter needed | European | Aurum |
| 8064041000006114 | Norwegian language interpreter needed | European | Aurum |
| 5841311000006113 | Polish language | European | Aurum |
| 678331000000111 | Kinyarwanda language interpreter needed | Africa | Aurum |
| 5845991000006117 | Hungarian language | European | Aurum |
| 1854161000006113 | preferred written language: Dutch | European | Aurum |
| 681151000000119 | Guarani language interpreter needed | Americas | Aurum |
| 5841281000006111 | Slovenian language | European | Aurum |
| 5845261000006116 | Pidgin English language | Africa | Aurum |
| 728541000000119 | main spoken language Iban | South Asian | Aurum |
| 674811000000116 | main spoken language Tongan | Australasia | Aurum |
| 5841091000006118 | Portuguese language | European | Aurum |
| 5840981000006114 | Hellenic language | European | Aurum |
| 1706301000006111 | main spoken language Limburgan | European | Aurum |
| 993041000006112 | Pashto as a second language | West & Central Asia | Aurum |
| 1862231000006114 | preferred written language: Guarani | Americas | Aurum |
| 5845411000006118 | Chinese language | East Asian | Aurum |
| 1166921000000115 | main spoken language Bamun | Africa | Aurum |
| 5845241000006115 | Krio language | Africa | Aurum |
| 8064121000006118 | Polish language interpreter needed | European | Aurum |
| 992971000006113 | Farsi as a second language | West & Central Asia | Aurum |
| 1862001000006112 | preferred written language: Chechen | European | Aurum |
| 992711000006119 | Mandarin as a second language | East Asian | Aurum |
| 8354971000006112 | Latvian as a second language | European | Aurum |
| 337036017 | second language |  | Aurum |
| 1862981000006116 | preferred written language: Sundanese | English/Unknown | Aurum |
| 1778581000006116 | preferred communication language: Italian | European | Aurum |
| 309351000000111 | Hebrew as a second language | West & Central Asia | Aurum |
| 1706051000006113 | main spoken language Chamorro | Australasia | Aurum |
| 1861841000006111 | preferred written language: Azerbaijani | European | Aurum |
| 1854601000006116 | preferred written language: Urdu | South Asian | Aurum |
| 5841521000006110 | Lehnda Punjabi language | South Asian | Aurum |
| 1863011000006119 | preferred written language: Tajik | West & Central Asia | Aurum |
| 674871000000114 | main spoken language Tsonga | Africa | Aurum |
| 671991000000116 | main spoken language Icelandic | European | Aurum |
| 671491000000112 | main spoken language Fijian | Australasia | Aurum |
| 5840481000006116 | Tulu language | South Asian | Aurum |
| 1862601000006115 | preferred written language: Malagasy | Africa | Aurum |
| 669981000000114 | main spoken language Abkhazian | European | Aurum |
| 673131000000115 | main spoken language Malay | East Asian | Aurum |
| 1672671000006110 | main spoken language Finnish | European | Aurum |
| 5843851000006115 | Fulani language | Africa | Aurum |
| 1854511000006119 | preferred written language: Spanish | European | Aurum |
| 1854441000006116 | preferred written language: Polish | European | Aurum |
| 1706181000006116 | main spoken language Gujarati | South Asian | Aurum |
| 5841841000006118 | Maba language | Africa | Aurum |
| 8064591000006111 | Ukrainian language interpreter needed | European | Aurum |
| 1862551000006117 | preferred written language: Limburgan | European | Aurum |
| 1706011000006112 | main spoken language Avaric | European | Aurum |
| 670401000000112 | main spoken language Azerbaijani | European | Aurum |
| 5840401000006113 | Kanarese language | South Asian | Aurum |
| 1706401000006119 | main spoken language Chichewa | Africa | Aurum |
| 8063661000006116 | German language interpreter needed | European | Aurum |
| 5839281000006113 | Kazakh language | West & Central Asia | Aurum |
| 460085018 | main spoken language Cantonese | East Asian | Aurum |
| 1778841000006112 | preferred communication language: Tigrinya | Africa | Aurum |
| 993491000006110 | language of interpreter - Gaelic | UK languages | Aurum |
| 671631000000113 | main spoken language Frisian | European | Aurum |
| 2160064011 | main spoken language Igbo | Africa | Aurum |
| 1706501000006118 | main spoken language Volapuk |  | Aurum |
| 1854551000006118 | preferred written language: Tagalog (Filipino) | East Asian | Aurum |
| 5843631000006111 | Ibo language | Africa | Aurum |
| 8063871000006110 | Lingala language interpreter needed | Africa | Aurum |
| 670721000000112 | main spoken language Bislama | Australasia | Aurum |
| 5839161000006110 | Turkic language | English/Unknown | Aurum |
| 684581000000119 | Rundi language interpreter needed | Africa | Aurum |
| 309081000000119 | Albanian as a second language | European | Aurum |
| 1862891000006114 | preferred written language: Sango | Africa | Aurum |
| 460092011 | main spoken language Hausa | Africa | Aurum |
| 1862831000006110 | preferred written language: Pali | South Asian | Aurum |
| 5843681000006112 | Nupe language | Africa | Aurum |
| 673971000000116 | main spoken language Sango | Africa | Aurum |
| 674511000000119 | main spoken language Sundanese | English/Unknown | Aurum |
| 460101014 | main spoken language Somali | Africa | Aurum |
| 5838761000006117 | Sidamo language | Africa | Aurum |
| 456771011 | language not recorded | English/Unknown | Aurum |
| 1862021000006119 | preferred written language: Chinese | East Asian | Aurum |
| 1854201000006119 | preferred written language: Finnish | European | Aurum |
| 1706091000006119 | main spoken language Chuvash | European | Aurum |
| 670851000000110 | main spoken language Burmese | East Asian | Aurum |
| 1862401000006117 | preferred written language: Kannada | South Asian | Aurum |
| 2160082014 | main spoken language Welsh | UK languages | Aurum |
| 309181000000115 | Croatian as a second language | European | Aurum |
| 2615851000000110 | Romany as second language | European | Aurum |
| 685851000000112 | southern Sotho language interpreter needed | Africa | Aurum |
| 7222471000006114 | communication using speech and sign language simultaneously | UK languages | Aurum |
| 993291000006111 | language of interpreter - Iba | South Asian | Aurum |
| 672831000000116 | main spoken language Lao | East Asian | Aurum |
| 8063761000006113 | Hebrew language interpreter needed | West & Central Asia | Aurum |
| 5840631000006119 | Indo-European language | European | Aurum |
| 7592341000006112 | British sign language | UK languages | Aurum |
| 5841961000006110 | Igorot language | East Asian | Aurum |
| 677411000000113 | Moldavian language interpreter needed | European | Aurum |
| 460097017 | main spoken language Polish | European | Aurum |
| 5838961000006119 | north Arabic language | West & Central Asia | Aurum |
| 1658291000000118 | main spoken language Tetum | East Asian | Aurum |
| 2159248015 | main spoken language Albanian | European | Aurum |
| 2418601000000115 | visual frame sign language interpreter needed | UK languages | Aurum |
| 684831000000116 | main spoken language Panjabi | South Asian | Aurum |
| 2160083016 | main spoken language Tigrinya | Africa | Aurum |
| 677711000000117 | Brawa language interpreter needed | Africa | Aurum |
| 5840461000006114 | Tamil language | South Asian | Aurum |
| 8063371000006117 | Bengali language interpreter needed | South Asian | Aurum |
| 5843771000006116 | Malinke language | Africa | Aurum |
| 682761000000111 | Assamese language interpreter needed | South Asian | Aurum |
| 5843711000006113 | Yoruba language | Africa | Aurum |
| 1861851000006113 | preferred written language: Bambara | Africa | Aurum |
| 685911000000118 | Tajik language interpreter needed | West & Central Asia | Aurum |
| 6763501000006112 | main spoken language French Creole | Americas | Aurum |
| 5840901000006117 | English language | English/Unknown | Aurum |
| 4538891000006113 | Pashtu language | West & Central Asia | Aurum |
| 672531000000118 | main spoken language Kashmiri | South Asian | Aurum |
| 5841271000006113 | Serbo-Croatian language | European | Aurum |
| 1778571000006119 | preferred communication language: Igbo (Ibo) | Africa | Aurum |
| 5840321000006114 | Luo language | Africa | Aurum |
| 1854231000006110 | preferred written language: French Creole | Americas | Aurum |
| 8355771000006114 | Wolof as a second language | Africa | Aurum |
| 674751000000113 | main spoken language Tibetan | South Asian | Aurum |
| 1862341000006111 | preferred written language: interlingua |  | Aurum |
| 1862581000006113 | preferred written language: Luxembourgish | European | Aurum |
| 2160071018 | main spoken language French Creole | Americas | Aurum |
| 8102661000006117 | using sign language to communicate with client | UK languages | Aurum |
| 5842081000006114 | Sundanese language | English/Unknown | Aurum |
| 5839001000006110 | Maltese language | European | Aurum |
| 1863221000006118 | preferred written language: Zulu | Africa | Aurum |
| 309941000000114 | Italian as a second language | European | Aurum |
| 309431000000111 | Korean as a second language | East Asian | Aurum |
| 1862631000006111 | preferred written language: Manx | UK languages | Aurum |
| 2615891000000119 | Romsky as a second language | European | Aurum |
| 309571000000114 | Serbian as a second language | European | Aurum |
| 1862121000006118 | preferred written language: Dzongkha | South Asian | Aurum |
| 674991000000119 | main spoken language Twi | Africa | Aurum |
| 460088016 | main spoken language French | European | Aurum |
| 681211000000114 | Georgian language interpreter needed | European | Aurum |
| 5841161000006113 | Spanish language | European | Aurum |
| 5840751000006116 | Irish Gaelic language | UK languages | Aurum |
| 664991000000112 | Bulgarian language interpreter needed | European | Aurum |
| 1706131000006117 | main spoken language Ewe | Africa | Aurum |
| 5841251000006115 | Bulgarian language | European | Aurum |
| 1706531000006114 | using Australian sign language | Sign language | Aurum |
| 676361000000117 | main spoken language Persian | West & Central Asia | Aurum |
| 8063991000006115 | Malayalam language interpreter needed | South Asian | Aurum |
| 1862781000006118 | preferred written language: Occitan | European | Aurum |
| 1656481000006113 | main spoken language Bulgarian | European | Aurum |
| 8063631000006113 | Igbo language interpreter needed | Africa | Aurum |
| 2643211000000115 | main spoken language Romanes | European | Aurum |
| 1862991000006118 | preferred written language: Swati | Africa | Aurum |
| 682051000000111 | Esperanto language interpreter needed |  | Aurum |
| 2638721000000114 | Romanesa language interpreter needed | European | Aurum |
| 992621000006119 | Cantonese as a second language | East Asian | Aurum |
| 5843581000006113 | Fanti language | Africa | Aurum |
| 1863071000006111 | preferred written language: Tsonga | Africa | Aurum |
| 310021000000112 | Vietnamese as a second language | East Asian | Aurum |
| 1863061000006116 | preferred written language: Tongan | Australasia | Aurum |
| 673851000000115 | main spoken language Romansh | European | Aurum |
| 993061000006111 | Patois as a second language | Americas | Aurum |
| 5840521000006116 | Fur language | Africa | Aurum |
| 310091000000110 | Lingala as a second language | Africa | Aurum |
| 683091000000110 | Burmese language interpreter needed | East Asian | Aurum |
| 675051000000110 | main spoken language Uighur | East Asian | Aurum |

## Code list for determining religion in CPRD

The CPRD Code Browser was used to generate a list of codes, both in CPRD Gold and CPRD Aurum. The search strategy included terms such as “*religion*”, “*Christian*”, “*Muslim*”, etc.

All codes that are related to religion were considered. The final code lists had 150 codes for CPRD Gold and 465 codes for CPRD Aurum.

| **Medcode** | **Description** | **Category** | **Source** |
| --- | --- | --- | --- |
| 2053 | Jehovah’s witness | Christian | Gold |
| 12477 | Jewish | Jew | Gold |
| 12622 | spiritualist | No answer/Unknown | Gold |
| 12685 | Hindu | Hindu | Gold |
| 13450 | minister of religion | No answer/Unknown | Gold |
| 19559 | religion, none | No religion | Gold |
| 22191 | religion | No answer/Unknown | Gold |
| 24262 | religion nos | Other religion | Gold |
| 24263 | church of England | Christian | Gold |
| 24268 | roman catholic | Christian | Gold |
| 24271 | religion not given - patient refused | No answer/Unknown | Gold |
| 24273 | Buddhist | Buddhist | Gold |
| 24297 | Sikh | Sikh | Gold |
| 24298 | religion not recorded | No answer/Unknown | Gold |
| 24341 | Islam | Muslim | Gold |
| 24360 | [v]refusal of treatment for reasons of religion/conscience | No answer/Unknown | Gold |
| 24669 | Christian | Christian | Gold |
| 24672 | closed fracture of seventh cervical vertebra | No answer/Unknown | Gold |
| 25594 | Baptist | Christian | Gold |
| 25996 | Muslim | Muslim | Gold |
| 26609 | p/n - seventh day visit | No answer/Unknown | Gold |
| 29954 | Christian scientist | Christian | Gold |
| 31044 | church of Scotland | Christian | Gold |
| 31585 | Plymouth brethren | Christian | Gold |
| 32381 | minister of religion | No answer/Unknown | Gold |
| 33795 | spiritual healing | No answer/Unknown | Gold |
| 39751 | methodist | Christian | Gold |
| 39815 | church - religion | Christian | Gold |
| 39839 | atheist | No religion | Gold |
| 39934 | [v]refusal of treatment for reasons of religion | No answer/Unknown | Gold |
| 40568 | procedure refused - religion | No answer/Unknown | Gold |
| 45923 | seventh cranial nerve injury | No answer/Unknown | Gold |
| 46063 | Jewish - ethnic category 2001 census | Jew | Gold |
| 46473 | place of occurrence of accident or poisoning, church | Christian | Gold |
| 47091 | Muslim - ethnic category 2001 census | Muslim | Gold |
| 47329 | salvation army | Christian | Gold |
| 47914 | salvation army member | Christian | Gold |
| 47959 | Pentecostal | Christian | Gold |
| 47961 | evangelical | Christian | Gold |
| 47962 | mixed religion | No answer/Unknown | Gold |
| 47968 | agnostic | No religion | Gold |
| 47971 | Anglican | Christian | Gold |
| 47972 | presbyterian | Christian | Gold |
| 47998 | orthodox Christian | Christian | Gold |
| 47999 | Jainism | Other religion | Gold |
| 48000 | united reform church | Christian | Gold |
| 48026 | contact dermatitis due to jewellery | Jew | Gold |
| 49658 | Sikh - ethnic category 2001 census | Sikh | Gold |
| 50229 | Rastafarian | Other religion | Gold |
| 52201 | Christadelphian | Christian | Gold |
| 56127 | Hindu - ethnic category 2001 census | Hindu | Gold |
| 57757 | nonconformist | Christian | Gold |
| 58665 | Sunni Muslim | Muslim | Gold |
| 59646 | spiritual care | No answer/Unknown | Gold |
| 63262 | humanistic and integrative therapy | Other religion | Gold |
| 63316 | hit by object falling from burning church | Christian | Gold |
| 63872 | Buddhist - ethnic category 2001 census | Buddhist | Gold |
| 64041 | Shiite Muslim | Muslim | Gold |
| 64056 | pagan | Other religion | Gold |
| 64057 | Mormon | Christian | Gold |
| 64058 | Moravian religion | Christian | Gold |
| 71012 | patient religion unknown | No answer/Unknown | Gold |
| 73262 | jump from burning church | Christian | Gold |
| 92227 | eastern catholic | Christian | Gold |
| 99738 | protestant | Christian | Gold |
| 100125 | church of England, follower of religion | Christian | Gold |
| 100433 | Armenian orthodox | Christian | Gold |
| 100522 | church of Scotland, follower of religion | Christian | Gold |
| 100526 | seventh day Adventist | Christian | Gold |
| 100536 | Scottish episcopalian | Christian | Gold |
| 100713 | pure land Buddhist | Buddhist | Gold |
| 100794 | French protestant | Christian | Gold |
| 100867 | African religion, follower of religion | Other religion | Gold |
| 100951 | Yoruba, follower of religion | Other religion | Gold |
| 101115 | Lutheran | Christian | Gold |
| 101384 | orthodox jew | Jew | Gold |
| 101828 | Ukrainian catholic | Christian | Gold |
| 101837 | zen Buddhist | Buddhist | Gold |
| 101856 | Greek orthodox | Christian | Gold |
| 102123 | native American religion, follower of religion | Other religion | Gold |
| 102124 | catholic: non roman catholic | Christian | Gold |
| 102238 | Judaic Christian | Christian | Gold |
| 102459 | follower of united reformed church | Christian | Gold |
| 102498 | wiccan | Other religion | Gold |
| 102646 | Shinto | Other religion | Gold |
| 102697 | Romanian orthodox | Christian | Gold |
| 102814 | complement seventh component | No answer/Unknown | Gold |
| 102991 | shakti Hindu | Hindu | Gold |
| 103692 | Russian orthodox | Christian | Gold |
| 103845 | humanist | Other religion | Gold |
| 103929 | free church of Scotland | Christian | Gold |
| 104111 | Bulgarian orthodox | Christian | Gold |
| 104723 | congregationalist | Christian | Gold |
| 104737 | Ashkenazi jew | Jew | Gold |
| 104893 | Christian existentialist | Christian | Gold |
| 104965 | Smarta Hindu | Hindu | Gold |
| 105327 | religion - further affiliations | No answer/Unknown | Gold |
| 105407 | deist | Other religion | Gold |
| 105515 | has spiritual and cultural support | No answer/Unknown | Gold |
| 105874 | Taoist | Other religion | Gold |
| 106119 | unitarian universalist | Other religion | Gold |
| 106166 | church caretaker | Christian | Gold |
| 106797 | Ethiopian orthodox tewahedo | Christian | Gold |
| 107174 | Rastafarian | Other religion | Gold |
| 107288 | messianic jew | Jew | Gold |
| 107636 | reformed Christian | Christian | Gold |
| 107810 | Ukrainian orthodox | Christian | Gold |
| 107871 | heathen | Other religion | Gold |
| 107962 | reform jew | Jew | Gold |
| 108014 | Celtic Christian | Christian | Gold |
| 108015 | Christian spiritualist | Christian | Gold |
| 108066 | free church | Christian | Gold |
| 108225 | Zoroastrian | Other religion | Gold |
| 108497 | Arya samaj Hindu | Hindu | Gold |
| 108571 | Celtic orthodox Christian | Christian | Gold |
| 109080 | Mahayana Buddhist | Buddhist | Gold |
| 109590 | church in Wales | Christian | Gold |
| 109843 | old catholic | Christian | Gold |
| 109854 | shiva Hindu | Hindu | Gold |
| 110027 | brethren | Christian | Gold |
| 110042 | Wesleyan methodist | Christian | Gold |
| 110235 | evangelical Christian | Christian | Gold |
| 110368 | Syrian orthodox | Christian | Gold |
| 110761 | spiritual assessment | No answer/Unknown | Gold |
| 111108 | closed dislocation of seventh cervical vertebra (c7) | No answer/Unknown | Gold |
| 111201 | meeting spiritual needs | No answer/Unknown | Gold |
| 111224 | Greek catholic | Christian | Gold |
| 111329 | free methodist | Christian | Gold |
| 111387 | unitarian | Christian | Gold |
| 111549 | Advaitin Hindu | Hindu | Gold |
| 111610 | Tibetan Buddhist | Buddhist | Gold |
| 112297 | Armenian catholic | Christian | Gold |
| 112787 | secularist | Other religion | Gold |
| 112966 | generic humanistic therapy | Other religion | Gold |
| 112983 | open fracture of seventh cervical vertebra | No answer/Unknown | Gold |
| 113035 | follower of church of Nazarene | Christian | Gold |
| 113748 | new kadampa tradition Buddhist | Buddhist | Gold |
| 113785 | salvation army member | Christian | Gold |
| 114093 | free presbyterian | Christian | Gold |
| 114239 | independent methodist | Christian | Gold |
| 114462 | universalist | Other religion | Gold |
| 114618 | Jain | Other religion | Gold |
| 114779 | reformed protestant | Christian | Gold |
| 115434 | church of Ireland, follower of religion | Christian | Gold |
| 115704 | Coptic orthodox | Christian | Gold |
| 116176 | religion - additional affiliations | No answer/Unknown | Gold |
| 22969017 | Mormon | Christian | Aurum |
| 46891017 | Shiite Muslim | Muslim | Aurum |
| 85962014 | Sephardic jew | Jew | Aurum |
| 135537015 | Ashkenazi jew | Jew | Aurum |
| 142840016 | Jainism | Other religion | Aurum |
| 143912017 | seventh | No answer/Unknown | Aurum |
| 169458012 | spiritual assessment | No answer/Unknown | Aurum |
| 249672018 | salvation army member | Christian | Aurum |
| 250254015 | church - religion | Christian | Aurum |
| 250255019 | religion | No answer/Unknown | Aurum |
| 250259013 | roman catholic | Christian | Aurum |
| 250262011 | atheist | No religion | Aurum |
| 250265013 | Jewish | Jew | Aurum |
| 250267017 | Christian scientist | Christian | Aurum |
| 250268010 | Hindu | Hindu | Aurum |
| 250274010 | Christian | Christian | Aurum |
| 250276012 | mixed religion | No answer/Unknown | Aurum |
| 250278013 | religion, none | No religion | Aurum |
| 250284011 | Baptist | Christian | Aurum |
| 250285012 | methodist | Christian | Aurum |
| 250286013 | united reform church | Christian | Aurum |
| 250287016 | presbyterian | Christian | Aurum |
| 250288014 | church of Scotland | Christian | Aurum |
| 250290010 | Pentecostal | Christian | Aurum |
| 250293012 | evangelical | Christian | Aurum |
| 250297013 | Plymouth brethren | Christian | Aurum |
| 250298015 | Christadelphian | Christian | Aurum |
| 250299011 | agnostic | No religion | Aurum |
| 371004012 | Rastafarian | Other religion | Aurum |
| 397728017 | religion nos | Other religion | Aurum |
| 406160013 | Islam | Muslim | Aurum |
| 406240013 | Anglican | Christian | Aurum |
| 412121016 | nonconformist | Christian | Aurum |
| 453156010 | Buddhist | Buddhist | Aurum |
| 453387016 | Muslim | Muslim | Aurum |
| 453388014 | protestant | Christian | Aurum |
| 453390010 | church of England | Christian | Aurum |
| 456655015 | religion not recorded | No answer/Unknown | Aurum |
| 477048013 | Hasidic jew | Jew | Aurum |
| 490271012 | Sikh | Sikh | Aurum |
| 504463011 | Jehovah’s witness | Christian | Aurum |
| 2691802017 | nichiren Buddhist | Buddhist | Aurum |
| 2691976017 | Armenian catholic | Christian | Aurum |
| 2692183014 | seventh day Adventist | Christian | Aurum |
| 2692283017 | wiccan | Other religion | Aurum |
| 2692313014 | Vaishnava Hindu | Hindu | Aurum |
| 2692541014 | Arya samaj Hindu | Hindu | Aurum |
| 2692549011 | Theravada Buddhist | Buddhist | Aurum |
| 2692578012 | orthodox Christian | Christian | Aurum |
| 2692643017 | Smarta Hindu | Hindu | Aurum |
| 2692745019 | new kadampa tradition Buddhist | Buddhist | Aurum |
| 2692760016 | Greek orthodox | Christian | Aurum |
| 2692957017 | shakti Hindu | Hindu | Aurum |
| 2693056016 | Mahayana Buddhist | Buddhist | Aurum |
| 2693198016 | haredi jew | Jew | Aurum |
| 2693924013 | Elim pentecostalist | Christian | Aurum |
| 2693950012 | unitarian | Christian | Aurum |
| 2693984011 | congregationalist | Christian | Aurum |
| 2694018011 | zen Buddhist | Buddhist | Aurum |
| 2694241011 | shiva Hindu | Hindu | Aurum |
| 2694381019 | advaitin Hindu | Hindu | Aurum |
| 2694444017 | Lutheran | Christian | Aurum |
| 2694490013 | Russian orthodox | Christian | Aurum |
| 2694508012 | Christian spiritualist | Christian | Aurum |
| 2694566014 | Tibetan Buddhist | Buddhist | Aurum |
| 2694716017 | masorti jew | Jew | Aurum |
| 2694761018 | Taoist | Other religion | Aurum |
| 2694817017 | Zoroastrian | Other religion | Aurum |
| 2694897016 | anabaptist | Christian | Aurum |
| 2695096016 | liberal jew | Jew | Aurum |
| 2695239015 | Shinto | Other religion | Aurum |
| 2695421010 | Coptic orthodox | Christian | Aurum |
| 2695527012 | Ethiopian orthodox tewahedo | Christian | Aurum |
| 2695628010 | pure land Buddhist | Buddhist | Aurum |
| 2695820017 | Jain | Other religion | Aurum |
| 2695980016 | orthodox jew | Jew | Aurum |
| 2696089014 | reform jew | Jew | Aurum |
| 120361000006112 | Sunni Muslim | Muslim | Aurum |
| 138241000000113 | Jewish - ethnic category 2001 census | Jew | Aurum |
| 138281000000117 | Muslim - ethnic category 2001 census | Muslim | Aurum |
| 138631000006113 | quaker religion | Christian | Aurum |
| 142881000000117 | Buddhist - ethnic category 2001 census | Buddhist | Aurum |
| 142891000000115 | Sikh - ethnic category 2001 census | Sikh | Aurum |
| 157351000000115 | Hindu - ethnic category 2001 census | Hindu | Aurum |
| 191981000006117 | Rastafarian | Other religion | Aurum |
| 314411000000116 | eastern catholic religion | Christian | Aurum |
| 314431000000112 | spiritualism | Other religion | Aurum |
| 314451000000117 | Moravian church | Christian | Aurum |
| 314461000000119 | Moravian | Christian | Aurum |
| 314611000000118 | pagan | Other religion | Aurum |
| 539441000000110 | humanist | Other religion | Aurum |
| 540101000000111 | heathen | Other religion | Aurum |
| 642431000000118 | apostolic pentecostalist | Christian | Aurum |
| 642491000000117 | Armenian orthodox | Christian | Aurum |
| 642511000000113 | follower of shamanism | Other religion | Aurum |
| 642581000000118 | church of God of prophecy | Christian | Aurum |
| 642621000000118 | church of Scotland, follower of religion | Christian | Aurum |
| 642701000000115 | free church | Christian | Aurum |
| 642731000000114 | free church of Scotland | Christian | Aurum |
| 642791000000110 | Nazarene church | Christian | Aurum |
| 642801000000114 | follower of church of Nazarene | Christian | Aurum |
| 642831000000115 | follower of New Testament church of god | Christian | Aurum |
| 642851000000110 | New Testament pentecostalist | Christian | Aurum |
| 643011000000114 | Serbian orthodox | Christian | Aurum |
| 643101000000113 | united reformed church | Christian | Aurum |
| 643111000000110 | follower of united reformed church | Christian | Aurum |
| 712831000000118 | Celtic pagan | Other religion | Aurum |
| 713111000000113 | shaman | Other religion | Aurum |
| 713191000000116 | African religion, follower of religion | Other religion | Aurum |
| 713251000000118 | Yoruba, follower of religion | Other religion | Aurum |
| 713371000000117 | Bulgarian orthodox | Christian | Aurum |
| 713401000000115 | catholic: non roman catholic | Christian | Aurum |
| 713431000000114 | Celtic Christian | Christian | Aurum |
| 713461000000116 | Celtic orthodox Christian | Christian | Aurum |
| 713491000000110 | Chinese evangelical Christian | Christian | Aurum |
| 713521000000113 | Christian existentialist | Christian | Aurum |
| 713551000000115 | Christian humanist | Other religion | Aurum |
| 713581000000114 | church in Wales | Christian | Aurum |
| 713621000000114 | church of Ireland, follower of religion | Christian | Aurum |
| 713641000000119 | eastern orthodox | Christian | Aurum |
| 713671000000113 | evangelical Christian | Christian | Aurum |
| 713701000000112 | free evangelical presbyterian | Christian | Aurum |
| 713731000000118 | free presbyterian | Christian | Aurum |
| 713761000000111 | Greek catholic | Christian | Aurum |
| 713791000000117 | Judaic Christian | Christian | Aurum |
| 713921000000117 | old catholic | Christian | Aurum |
| 713951000000110 | reformed Christian | Christian | Aurum |
| 713981000000116 | reformed presbyterian | Christian | Aurum |
| 714011000000114 | reformed protestant | Christian | Aurum |
| 714041000000110 | Romanian orthodox | Christian | Aurum |
| 714111000000110 | Syrian orthodox | Christian | Aurum |
| 714141000000111 | Ukrainian catholic | Christian | Aurum |
| 714421000000114 | universalist | Other religion | Aurum |
| 714451000000116 | unitarian universalist | Other religion | Aurum |
| 714481000000110 | messianic jew | Jew | Aurum |
| 714711000000111 | native American religion, follower of religion | Other religion | Aurum |
| 729271000000115 | Scottish episcopalian | Christian | Aurum |
| 734861000000110 | secularist | Other religion | Aurum |
| 734911000000118 | free methodist | Christian | Aurum |
| 734941000000117 | independent methodist | Christian | Aurum |
| 734971000000111 | Indian orthodox | Christian | Aurum |
| 735001000000115 | Ukrainian orthodox | Christian | Aurum |
| 735041000000117 | brethren | Christian | Aurum |
| 735091000000110 | French protestant | Christian | Aurum |
| 940211000006118 | orthodox Jewish | Jew | Aurum |
| 958581000006112 | spirituality | Other religion | Aurum |
| 974321000006110 | spirituality nos | Other religion | Aurum |
| 987431000006115 | Jainism | Other religion | Aurum |
| 991941000006114 | arcane school religion | Other religion | Aurum |
| 991951000006111 | arminianist religion | Other religion | Aurum |
| 991961000006113 | Ashkenazi Jewish religion | Jew | Aurum |
| 991971000006118 | babis religion | Other religion | Aurum |
| 991981000006115 | Baha’is religion | No answer/Unknown | Aurum |
| 991991000006117 | Calvinist religion | Other religion | Aurum |
| 992001000006115 | central Asian Buddhist religion | Buddhist | Aurum |
| 992011000006117 | Chinese Buddhist religion | Buddhist | Aurum |
| 992021000006113 | church of Ireland | Christian | Aurum |
| 992031000006111 | Confucian religion | Other religion | Aurum |
| 992041000006118 | congregationalist religion | Christian | Aurum |
| 992051000006116 | conservative Jewish religion | Jew | Aurum |
| 992061000006119 | eckankar religion | Other religion | Aurum |
| 992071000006114 | eminist religion | Other religion | Aurum |
| 992081000006112 | Isma’ili’s religion | No answer/Unknown | Aurum |
| 992091000006110 | Japanese Buddhist religion | Buddhist | Aurum |
| 992101000006116 | Krishna consciousness religious movement | Christian | Aurum |
| 992111000006118 | lamaist religion | Buddhist | Aurum |
| 992121000006114 | liberal Jewish religion | Jew | Aurum |
| 992131000006112 | liberal protestant religion | Christian | Aurum |
| 992141000006119 | Lutheran religion | Christian | Aurum |
| 992151000006117 | Mahayana Buddhist religion | Buddhist | Aurum |
| 992181000006113 | orthodox Christian religion | Christian | Aurum |
| 992211000006112 | primal society religion | Other religion | Aurum |
| 992221000006116 | protestant religion | Christian | Aurum |
| 992231000006118 | pietist religion | Christian | Aurum |
| 992241000006111 | quaker religion | Christian | Aurum |
| 992251000006113 | reconstructionist Jewish religion | Jew | Aurum |
| 992261000006110 | reform Jewish religion | Jew | Aurum |
| 992271000006115 | Scottish protestant religion | Christian | Aurum |
| 992281000006117 | sefardi Jewish religion | Jew | Aurum |
| 992291000006119 | seventh day Adventist religion | Christian | Aurum |
| 992301000006118 | shintoist religion | Other religion | Aurum |
| 992311000006115 | Sinhalese Buddhist religion | Buddhist | Aurum |
| 992321000006111 | southeast Asia Buddhist religion | Buddhist | Aurum |
| 992331000006114 | Sufi Muslim religion | Muslim | Aurum |
| 992341000006116 | Theravada Buddhist religion | Buddhist | Aurum |
| 992351000006119 | Tibetan Buddhist religion | Buddhist | Aurum |
| 992361000006117 | unification church religious movement | Christian | Aurum |
| 992371000006112 | unitarian religion | Christian | Aurum |
| 992381000006110 | western Buddhist religion | Buddhist | Aurum |
| 992391000006113 | episcopal church | Christian | Aurum |
| 992401000006110 | Wesleyan methodist | Christian | Aurum |
| 992411000006113 | Russian orthodox church | Christian | Aurum |
| 992421000006117 | Greek orthodox church | Christian | Aurum |
| 992431000006119 | Ukrainian catholic church | Christian | Aurum |
| 992441000006112 | chapel religion | Christian | Aurum |
| 992451000006114 | New Testament church | Christian | Aurum |
| 992471000006116 | new apostolic church | Christian | Aurum |
| 992481000006118 | Welsh independent church | Christian | Aurum |
| 992491000006115 | church of God | Christian | Aurum |
| 992501000006111 | church of Christ | Christian | Aurum |
| 992551000006110 | Serbian orthodox church | Christian | Aurum |
| 992571000006117 | druid | Other religion | Aurum |
| 1058761000000113 | Uniate catholic | Christian | Aurum |
| 1672271000006113 | orthodox Jewish faith | Jew | Aurum |
| 1672281000006111 | Sunni Muslim religion | Muslim | Aurum |
| 1725191000006116 | new kadampa tradition Buddhist, follower of religion | Buddhist | Aurum |
| 1725201000006118 | nichiren Buddhist, follower of religion | Buddhist | Aurum |
| 1725211000006115 | pure land Buddhist, follower of religion | Buddhist | Aurum |
| 1725221000006111 | zen Buddhist, follower of religion | Buddhist | Aurum |
| 1725251000006119 | catholic: not roman catholic | Christian | Aurum |
| 1725281000006110 | wiccan, follower of religion | Other religion | Aurum |
| 1725291000006113 | Zoroastrian, follower of religion | Other religion | Aurum |
| 1725311000006112 | animist, follower of religion | Other religion | Aurum |
| 1725341000006111 | brahma Kumari, follower of religion | Other religion | Aurum |
| 1725371000006115 | deist, follower of religion | Other religion | Aurum |
| 1725381000006117 | humanist | Other religion | Aurum |
| 1725401000006117 | kabbalist, follower of religion | Other religion | Aurum |
| 1725441000006115 | peyotist, follower of religion | Other religion | Aurum |
| 1725461000006116 | pantheist, follower of religion | Other religion | Aurum |
| 1725481000006114 | Santeria, follower of religion | Other religion | Aurum |
| 1725501000006116 | secularist, follower of religion | Other religion | Aurum |
| 1725511000006118 | Shumei, follower of religion | Other religion | Aurum |
| 1725521000006114 | Taoist, follower of religion | Other religion | Aurum |
| 1725531000006112 | universalist, follower of religion | Other religion | Aurum |
| 1725541000006119 | unitarian universalist, follower of religion | Other religion | Aurum |
| 1725571000006110 | Celtic Christian, follower of religion | Christian | Aurum |
| 1725581000006113 | Celtic orthodox Christian, follower of religion | Christian | Aurum |
| 1725591000006111 | Christian existentialist, follower of religion | Christian | Aurum |
| 1725601000006115 | Christian humanist, follower of religion | Other religion | Aurum |
| 1725611000006117 | Christian spiritualist, follower of religion | Christian | Aurum |
| 1725621000006113 | Judaic Christian, follower of religion | Christian | Aurum |
| 1725631000006111 | reformed Christian, follower of religion | Christian | Aurum |
| 1725641000006118 | Elim pentecostalist, follower of religion | Christian | Aurum |
| 1725671000006114 | reformed presbyterian, follower of religion | Christian | Aurum |
| 1725681000006112 | free methodist, follower of religion | Christian | Aurum |
| 1725731000006119 | advaitin Hindu, follower of religion | Hindu | Aurum |
| 1725741000006112 | Arya samaj Hindu, follower of religion | Hindu | Aurum |
| 1725751000006114 | shakti Hindu, follower of religion | Hindu | Aurum |
| 1725761000006111 | shiva Hindu, follower of religion | Hindu | Aurum |
| 1725771000006116 | Vaishnava Hindu, follower of religion | Hindu | Aurum |
| 1725781000006118 | Ahmadi, follower of religion | Muslim | Aurum |
| 1725801000006119 | Celtic pagan, follower of religion | Other religion | Aurum |
| 1725811000006116 | Baha’i, follower of religion | No answer/Unknown | Aurum |
| 1725821000006112 | Mahayana Buddhist, follower of religion | Buddhist | Aurum |
| 1725831000006110 | Theravada Buddhist, follower of religion | Buddhist | Aurum |
| 1725841000006117 | Tibetan Buddhist, follower of religion | Buddhist | Aurum |
| 1725861000006118 | follower of church of England | Christian | Aurum |
| 1725901000006113 | evangelical Christian, follower of religion | Christian | Aurum |
| 1725921000006115 | Chinese evangelical Christian, follower of religion | Christian | Aurum |
| 1725941000006110 | Swedenborgian, follower of religion | Christian | Aurum |
| 1725951000006112 | messianic jew, follower of religion | Jew | Aurum |
| 1725961000006114 | Scottish episcopalian, follower of religion | Christian | Aurum |
| 1725971000006119 | Greek orthodox, follower of religion | Christian | Aurum |
| 1725991000006118 | Mormon, follower of religion | Christian | Aurum |
| 1726001000006117 | protestant, follower of religion | Christian | Aurum |
| 1726011000006119 | scientologist, follower of church of scientology | Other religion | Aurum |
| 1726021000006110 | Plymouth brethren, follower of religion | Christian | Aurum |
| 1726051000006118 | quaker, follower of religion | Christian | Aurum |
| 1726061000006116 | Russian orthodox, follower of religion | Christian | Aurum |
| 1726081000006114 | Serbian orthodox, follower of religion | Christian | Aurum |
| 1726091000006112 | seventh day Adventist, follower of religion | Christian | Aurum |
| 1726101000006118 | Ukrainian catholic, follower of religion | Christian | Aurum |
| 1726111000006115 | unitarian, follower of religion | Christian | Aurum |
| 1726131000006114 | Jain, follower of religion | Other religion | Aurum |
| 1726141000006116 | Ashkenazi jew, follower of religion | Jew | Aurum |
| 1726151000006119 | liberal jew, follower of religion | Jew | Aurum |
| 1726161000006117 | orthodox jew, follower of religion | Jew | Aurum |
| 1726171000006112 | reform jew, follower of religion | Jew | Aurum |
| 1726191000006113 | Shinto, follower of religion | Other religion | Aurum |
| 1726201000006111 | apostolic pentecostalist, follower of religion | Christian | Aurum |
| 1726211000006114 | Armenian catholic, follower of religion | Christian | Aurum |
| 1726221000006118 | Armenian orthodox, follower of religion | Christian | Aurum |
| 1726231000006115 | Bulgarian orthodox, follower of religion | Christian | Aurum |
| 1726241000006113 | Coptic orthodox, follower of religion | Christian | Aurum |
| 1726251000006110 | eastern orthodox, follower of religion | Christian | Aurum |
| 1726261000006112 | Ethiopian orthodox | Christian | Aurum |
| 1726271000006117 | Indian orthodox, follower of religion | Christian | Aurum |
| 1726281000006119 | Romanian orthodox, follower of religion | Christian | Aurum |
| 1726301000006115 | Ukrainian orthodox, follower of religion | Christian | Aurum |
| 1726311000006117 | brethren, follower of religion | Christian | Aurum |
| 1726321000006113 | church in Wales, follower of religion | Christian | Aurum |
| 1726331000006111 | church of God of prophecy, follower of religion | Christian | Aurum |
| 1726351000006116 | free church of Scotland, follower of religion | Christian | Aurum |
| 1726371000006114 | Greek catholic, follower of religion | Christian | Aurum |
| 1726381000006112 | Mennonite, follower of religion | Christian | Aurum |
| 1726391000006110 | old catholic, follower of religion | Christian | Aurum |
| 1726401000006112 | reformed protestant, follower of religion | Christian | Aurum |
| 1726411000006110 | Uniate catholic, follower of religion | Christian | Aurum |
| 1726471000006118 | Ismaili Muslim, follower of religion | Muslim | Aurum |
| 1726501000006113 | follower of free Christian church | Christian | Aurum |
| 1737121000006112 | salvation army member | Christian | Aurum |
| 1737711000006117 | Sanatana dharma, follower of religion | Hindu | Aurum |
| 1738021000006112 | follower of church of England | Christian | Aurum |
| 1738071000006113 | Wesleyan methodist | Christian | Aurum |
| 1804961000006116 | uniting church in Australia | Christian | Aurum |
| 1804981000006114 | churches of Christ | Christian | Aurum |
| 1804991000006112 | churches of Christ, follower of religion | Christian | Aurum |
| 1805001000006117 | oriental orthodox | Christian | Aurum |
| 1805011000006119 | Assyrian church of the east | Christian | Aurum |
| 1805021000006110 | Assyrian church of the east, follower of religion | Christian | Aurum |
| 2712991000006117 | church of Jesus Christ of latter-day saints | Christian | Aurum |
| 2713021000006114 | Mormon religion | Christian | Aurum |
| 2713031000006112 | church of Jesus Christ of latter-day saint | Christian | Aurum |
| 2739151000006112 | Greek orthodox church | Christian | Aurum |
| 2796921000006116 | Shintoism | Other religion | Aurum |
| 2949541000006113 | Shiite Muslim, follower of religion | Muslim | Aurum |
| 2958571000006110 | methodist church | Christian | Aurum |
| 2968391000006114 | Serbian orthodox church | Christian | Aurum |
| 3014141000006113 | Lutheran church | Christian | Aurum |
| 3025481000006115 | Baptist church | Christian | Aurum |
| 3145601000006115 | Russian orthodox church | Christian | Aurum |
| 3186831000006114 | evangelical church | Christian | Aurum |
| 3413131000006114 | Anglican church | Christian | Aurum |
| 3413141000006116 | episcopal church | Christian | Aurum |
| 3493721000006113 | Hinduism | Hindu | Aurum |
| 3557061000006114 | Sunni Muslim, follower of religion | Muslim | Aurum |
| 3605071000006112 | Sikhism | Sikh | Aurum |
| 3605081000006110 | Sikh religion | Sikh | Aurum |
| 3646121000006112 | presbyterian church | Christian | Aurum |
| 3810751000006117 | Jehovah’s witness, follower of religion | Christian | Aurum |
| 3810771000006110 | Jehovah witness, follower of religion | Christian | Aurum |
| 3828731000006119 | Ashkenazi jew, follower of religion | Jew | Aurum |
| 3900471000006111 | Jain religion | Other religion | Aurum |
| 4153301000006110 | Islam and/or derivative | Muslim | Aurum |
| 4532681000006114 | roman catholic, follower of religion | Christian | Aurum |
| 4532711000006110 | non-believer | No religion | Aurum |
| 4532721000006119 | Jewish, follower of religion | Jew | Aurum |
| 4532751000006111 | Christian scientist, follower of religion | Christian | Aurum |
| 4532781000006115 | Hindu, follower of religion | Hindu | Aurum |
| 4532801000006116 | Christian, follower of religion | Christian | Aurum |
| 4532901000006110 | Baptist, follower of religion | Christian | Aurum |
| 4532921000006117 | methodist, follower of religion | Christian | Aurum |
| 4532941000006112 | united reformed church | Christian | Aurum |
| 4532961000006111 | presbyterian, follower of religion | Christian | Aurum |
| 4532991000006115 | church of Scotland religion | Christian | Aurum |
| 4533001000006119 | pentecostalist, follower of religion | Christian | Aurum |
| 4533021000006112 | pentecostalist | Christian | Aurum |
| 4533041000006117 | evangelist, follower of religion | No answer/Unknown | Aurum |
| 4533091000006114 | Plymouth brethren religion | Christian | Aurum |
| 4533111000006117 | Christadelphian, follower of religion | Christian | Aurum |
| 4952701000006117 | Muslim diet | Muslim | Aurum |
| 4952731000006113 | Sikh diet | Sikh | Aurum |
| 5258781000006115 | Rastafarian, follower of religion | Other religion | Aurum |
| 5495861000006117 | nonconformist religion | Christian | Aurum |
| 5524081000006117 | Muslim religion | Muslim | Aurum |
| 5580941000006112 | agnostic movement | No religion | Aurum |
| 5580961000006111 | atheist movement | No religion | Aurum |
| 5581011000006116 | Christian religion | Christian | Aurum |
| 5581051000006115 | Christadelphian movement | Christian | Aurum |
| 5581061000006118 | Jehovah’s witness religion | Christian | Aurum |
| 5581071000006113 | Jehovah witness religion | Christian | Aurum |
| 5581091000006114 | catholic religion | Christian | Aurum |
| 5581151000006116 | Christian scientist religious movement | Christian | Aurum |
| 5581161000006119 | Rastafarian movement | Other religion | Aurum |
| 5687861000006110 | church | Christian | Aurum |
| 5847411000006112 | orthodox Christian religion | Christian | Aurum |
| 5847541000006112 | Shi’ite Muslim religion | Muslim | Aurum |
| 5991891000006112 | Buddhist, follower of religion | Buddhist | Aurum |
| 5993421000006112 | pentecostalist religion | Christian | Aurum |
| 5993601000006117 | Muslim, follower of religion | Muslim | Aurum |
| 5993621000006110 | protestant, follower of religion | Christian | Aurum |
| 5993641000006115 | quaker, follower of religion | Christian | Aurum |
| 6218141000006119 | Hasidic jew, follower of religion | Jew | Aurum |
| 6296681000006115 | Sikh, follower of religion | Sikh | Aurum |
| 7077881000006117 | Elim pentecostalist, follower of religion | Christian | Aurum |
| 7078251000006110 | liberal jew, follower of religion | Jew | Aurum |
| 7078281000006119 | Lutheran, follower of religion | Christian | Aurum |
| 7080171000006116 | congregationalist, follower of religion | Christian | Aurum |
| 7081201000006110 | haredi jew, follower of religion | Jew | Aurum |
| 7081421000006118 | advaitin Hindu, follower of religion | Hindu | Aurum |
| 7081631000006117 | zen Buddhist, follower of religion | Buddhist | Aurum |
| 7086661000006115 | anabaptist, follower of religion | Christian | Aurum |
| 7087141000006111 | Arya samaj Hindu, follower of religion | Hindu | Aurum |
| 7087551000006115 | Coptic orthodox, follower of religion | Christian | Aurum |
| 7087661000006117 | Ethiopian orthodox tewahedo, follower of religion | Christian | Aurum |
| 7087671000006112 | Ethiopian orthodox | Christian | Aurum |
| 7088151000006112 | Theravada Buddhist, follower of religion | Buddhist | Aurum |
| 7088171000006119 | wiccan, follower of religion | Other religion | Aurum |
| 7088201000006115 | shakti Hindu, follower of religion | Hindu | Aurum |
| 7089441000006112 | Tibetan Buddhist, follower of religion | Buddhist | Aurum |
| 7089531000006115 | orthodox Christian, follower of religion | Christian | Aurum |
| 7089551000006110 | Christian spiritualist, follower of religion | Christian | Aurum |
| 7091861000006114 | nichiren Buddhist, follower of religion | Buddhist | Aurum |
| 7094101000006119 | Ahmadi, follower of religion | Muslim | Aurum |
| 7094121000006112 | Armenian catholic, follower of religion | Christian | Aurum |
| 7094191000006114 | seventh day Adventist, follower of religion | Christian | Aurum |
| 7099161000006119 | Greek orthodox, follower of religion | Christian | Aurum |
| 7102411000006111 | shiva Hindu, follower of religion | Hindu | Aurum |
| 7104491000006111 | Vaishnava Hindu, follower of religion | Hindu | Aurum |
| 7104771000006115 | new kadampa tradition Buddhist, follower of religion | Buddhist | Aurum |
| 7104871000006112 | Mahayana Buddhist, follower of religion | Buddhist | Aurum |
| 7104891000006113 | masorti jew, follower of religion | Jew | Aurum |
| 7104991000006117 | Calvinist, follower of religion | Other religion | Aurum |
| 7105061000006113 | Taoist, follower of religion | Other religion | Aurum |
| 7105081000006115 | Russian orthodox, follower of religion | Christian | Aurum |
| 7105131000006115 | Shinto, follower of religion | Other religion | Aurum |
| 7106461000006113 | orthodox jew, follower of religion | Jew | Aurum |
| 7106481000006115 | orthodox Jewish | Jew | Aurum |
| 7107521000006118 | unitarian, follower of religion | Christian | Aurum |
| 7107891000006118 | Baha’i, follower of religion | No answer/Unknown | Aurum |
| 7108701000006118 | Jain, follower of religion | Other religion | Aurum |
| 7108751000006119 | Zoroastrian, follower of religion | Other religion | Aurum |
| 7211011000006112 | Ukrainian catholic church | Christian | Aurum |
| 7299181000006116 | apostolic pentecostalist, follower of religion | Christian | Aurum |
| 8110871000006112 | scientologist, follower of church of scientology | Other religion | Aurum |
| 8110891000006113 | Mormon, follower of religion | Christian | Aurum |
| 8110981000006115 | Armenian orthodox, follower of religion | Christian | Aurum |
| 8111011000006118 | church of England | Christian | Aurum |
| 8111041000006119 | church of God of prophecy, follower of religion | Christian | Aurum |
| 8111081000006113 | follower of free Christian church | Christian | Aurum |
| 8111101000006117 | free church of Scotland, follower of religion | Christian | Aurum |
| 8111231000006119 | exclusive brethren, follower of religion | Christian | Aurum |
| 8111261000006111 | Serbian orthodox, follower of religion | Christian | Aurum |
| 8118931000006119 | anthroposophist, follower of religion | Other religion | Aurum |
| 8118991000006115 | Asatruan, follower of religion | Other religion | Aurum |
| 8119021000006113 | black magic, follower of religion | Other religion | Aurum |
| 8119051000006116 | brahma Kumari, follower of religion | Other religion | Aurum |
| 8119081000006112 | Celtic pagan, follower of religion | Other religion | Aurum |
| 8119121000006114 | Confucianist, follower of religion | Other religion | Aurum |
| 8119141000006119 | kabbalist, follower of religion | Other religion | Aurum |
| 8119181000006113 | lightworker, follower of religion | Other religion | Aurum |
| 8119411000006113 | Amish, follower of religion | No answer/Unknown | Aurum |
| 8119431000006119 | British Israelite, follower of religion | Jew | Aurum |
| 8119471000006116 | Bulgarian orthodox, follower of religion | Christian | Aurum |
| 8119491000006115 | catholic: non roman catholic, follower of religion | Christian | Aurum |
| 8119511000006114 | Celtic Christian, follower of religion | Christian | Aurum |
| 8119531000006115 | Celtic orthodox Christian, follower of religion | Christian | Aurum |
| 8119551000006110 | Chinese evangelical Christian, follower of religion | Christian | Aurum |
| 8119571000006117 | Christian existentialist, follower of religion | Christian | Aurum |
| 8119591000006116 | Christian humanist, follower of religion | Other religion | Aurum |
| 8119611000006110 | church in Wales, follower of religion | Christian | Aurum |
| 8119651000006111 | eastern orthodox, follower of religion | Christian | Aurum |
| 8119671000006118 | evangelical Christian, follower of religion | Christian | Aurum |
| 8119691000006117 | free evangelical presbyterian, follower of religion | Christian | Aurum |
| 8119721000006110 | free presbyterian, follower of religion | Christian | Aurum |
| 8119741000006115 | Greek catholic, follower of religion | Christian | Aurum |
| 8119761000006116 | Judaic Christian, follower of religion | Christian | Aurum |
| 8119811000006111 | old catholic, follower of religion | Christian | Aurum |
| 8119831000006117 | reformed Christian, follower of religion | Christian | Aurum |
| 8119871000006119 | reformed protestant, follower of religion | Christian | Aurum |
| 8119891000006118 | Romanian orthodox, follower of religion | Christian | Aurum |
| 8119931000006110 | Swedenborgian, follower of religion | Christian | Aurum |
| 8119971000006113 | Syrian orthodox, follower of religion | Christian | Aurum |
| 8119991000006114 | Ukrainian catholic, follower of religion | Christian | Aurum |
| 8120041000006119 | Radha soami, follower of religion | Other religion | Aurum |
| 8120081000006113 | infinite way, follower of religion | Other religion | Aurum |
| 8120111000006119 | deist, follower of religion | Other religion | Aurum |
| 8120131000006113 | Druze, follower of religion | Muslim | Aurum |
| 8120191000006112 | pantheist, follower of religion | Other religion | Aurum |
| 8120231000006119 | universalist, follower of religion | Other religion | Aurum |
| 8120251000006114 | unitarian universalist, follower of religion | Other religion | Aurum |
| 8120851000006113 | Scottish episcopalian, follower of religion | Christian | Aurum |
| 8121591000006110 | animist, follower of religion | Other religion | Aurum |
| 8121611000006116 | ancestral worship, follower of religion | Other religion | Aurum |
| 8121631000006110 | secularist, follower of religion | Other religion | Aurum |
| 8121651000006115 | free methodist, follower of religion | Christian | Aurum |
| 8121671000006113 | independent methodist, follower of religion | Christian | Aurum |
| 8121691000006114 | Indian orthodox, follower of religion | Christian | Aurum |
| 8121711000006112 | Ukrainian orthodox, follower of religion | Christian | Aurum |
| 8121731000006118 | brethren, follower of religion | Christian | Aurum |
| 8121771000006115 | French protestant, follower of religion | Christian | Aurum |
| 8186601000006118 | chondogyo, follower of religion | Other religion | Aurum |
| 8186621000006111 | Uniate catholic, follower of religion | Christian | Aurum |
| 13809441000006119 | seventh day Adventist religion | Christian | Aurum |

## Code list for determining sexual orientation in CPRD

The CPRD Code Browser was used to generate a list of codes, both in CPRD Gold and CPRD Aurum. The search strategy included terms such as “*homosex*”, “*heterosex*”, “*lesbian*”, etc.

All codes that are related to sexual orientation were considered. The final code lists had 20 codes for CPRD Gold and 39 codes for CPRD Aurum.

| **Medcode** | **Description** | **Category** | **Source** |
| --- | --- | --- | --- |
| 1620 | homosexuality | Homosexual | Gold |
| 4510 | lesbianism | Homosexual | Gold |
| 22818 | bisexual | Bisexual | Gold |
| 35254 | male homosexuality | Homosexual | Gold |
| 55664 | female homosexuality | Homosexual | Gold |
| 56824 | homosexuality nos | Homosexual | Gold |
| 85654 | heterosexual | Heterosexual | Gold |
| 105754 | homosexual activity | Homosexual | Gold |
| 106319 | lesbianism | Homosexual | Gold |
| 106320 | heterosexual | Heterosexual | Gold |
| 106321 | homosexuality | Homosexual | Gold |
| 106461 | homosexual | Homosexual | Gold |
| 106584 | lesbian | Homosexual | Gold |
| 106605 | bisexual | Bisexual | Gold |
| 106732 | male homosexual | Homosexual | Gold |
| 107304 | male homosexuality | Homosexual | Gold |
| 107633 | homosexuality nos | Homosexual | Gold |
| 108396 | female homosexuality | Homosexual | Gold |
| 480697011 | heterosexual | Heterosexual | Aurum |
| 491706014 | homosexual | Homosexual | Aurum |
| 503200017 | male homosexual | Homosexual | Aurum |
| 1235434019 | lesbian | Homosexual | Aurum |
| 2955565014 | sexually attracted to neither gender | Other | Aurum |
| 1672441000006116 | homosexual marriage, female | Homosexual | Aurum |
| 1672451000006119 | homosexual marriage, male | Homosexual | Aurum |
| 1762691000006112 | homosexual | Homosexual | Aurum |
| 1805921000006114 | gay/lesbian | Homosexual | Aurum |
| 1839401000006116 | sexual partner - male | Unknown | Aurum |
| 1839411000006118 | sexual partner - female | Unknown | Aurum |
| 1839421000006114 | sexual partners - male and female | Bisexual | Aurum |
| 1842531000006118 | homosexual activity | Homosexual | Aurum |
| 1864861000006113 | bisexual | Bisexual | Aurum |
| 1864871000006118 | heterosexual | Heterosexual | Aurum |
| 1864891000006117 | homosexuality | Homosexual | Aurum |
| 1864911000006115 | female homosexuality | Homosexual | Aurum |
| 1864921000006111 | female homosexual | Homosexual | Aurum |
| 1864931000006114 | male homosexuality | Homosexual | Aurum |
| 1864951000006119 | homosexuality | Homosexual | Aurum |
| 2015881000006115 | person stated sexual orientation: heterosexual or straight | Heterosexual | Aurum |
| 2015891000006117 | person stated sexual orientation: gay or lesbian | Homosexual | Aurum |
| 2015901000006118 | person stated sexual orientation: bisexual | Bisexual | Aurum |
| 2015911000006115 | person stated sexual orientation: other sexual orientation not listed | Other | Aurum |
| 2824711000006111 | heterosexual orientation - straight | Heterosexual | Aurum |
| 3121771000006116 | gay | Homosexual | Aurum |
| 3737951000006113 | gay man | Homosexual | Aurum |
| 3737961000006110 | male homosexual - gay | Homosexual | Aurum |
| 3950261000006113 | female homosexual - lesbian | Homosexual | Aurum |
| 4942571000006115 | homosexual behaviour | Homosexual | Aurum |
| 4980431000006110 | heterosexual relationship | Heterosexual | Aurum |
| 4980441000006117 | homosexual relationship | Homosexual | Aurum |
| 4980461000006118 | bisexual - predominantly heterosexual | Bisexual | Aurum |
| 4980471000006113 | bisexual - predominantly homosexual | Bisexual | Aurum |
| 5450161000006110 | straight | Heterosexual | Aurum |
| 12029251000006119 | sexually attracted to male sex | Unknown | Aurum |
| 12029271000006112 | sexually attracted to male and female sex | Bisexual | Aurum |
| 12029281000006110 | sexually attracted to neither male nor female sex | Other | Aurum |
| 14091951000006111 | asexual | Other | Aurum |

## Code list for determining gender identity in CPRD

The CPRD Code Browser was used to generate a list of codes, both in CPRD Gold and CPRD Aurum. The search strategy included terms such as “*gender*”, “*trans*sex*”, etc.

All codes that are related to gender identity were considered. The final code lists had 26 codes for CPRD Gold and 83 codes for CPRD Aurum.

| **Medcode** | **Description** | **Category** | **Source** |
| --- | --- | --- | --- |
| 9432 | operation for sexual transformation nos | Transgender | Gold |
| 9790 | [x]gender identity disorders | Other/Unknown | Gold |
| 9994 | operations for sexual transformation | Transgender | Gold |
| 20043 | transvestism | Transgender | Gold |
| 27765 | gender role disorder of adolescent or adult | Other/Unknown | Gold |
| 29841 | trans-sexualism | Transgender | Gold |
| 31124 | [x]transsexualism | Transgender | Gold |
| 37684 | [x]gender identity disorder, unspecified | Other/Unknown | Gold |
| 40084 | [x]gender-role disorder nos | Other/Unknown | Gold |
| 43170 | trans-sexuality with asexual history | Transgender | Gold |
| 43836 | [x]other gender identity disorders | Other/Unknown | Gold |
| 51983 | [x]fetishistic transvestism | Transgender | Gold |
| 54206 | trans-sexualism nos | Transgender | Gold |
| 62540 | [x]dual-role transvestism | Transgender | Gold |
| 63926 | gender unknown | Other/Unknown | Gold |
| 64520 | [x]gender identity disorder of childhood | Other/Unknown | Gold |
| 66734 | trans-sexuality with unspecified sexual history | Transgender | Gold |
| 66865 | trans-sexuality with homosexual history | Transgender | Gold |
| 70940 | trans-sexuality with heterosexual history | Transgender | Gold |
| 73326 | gender unspecified | Other/Unknown | Gold |
| 93391 | gender reassignment patient | Transgender | Gold |
| 109031 | [v] gender dysphoria | Other/Unknown | Gold |
| 114699 | gender identity observations | Other/Unknown | Gold |
| 114756 | seen in gender identity clinic | Other/Unknown | Gold |
| 278517011 | other specified operation for sexual transformation | Transgender | Aurum |
| 278518018 | operation for sexual transformation nos | Transgender | Aurum |
| 295093014 | transvestism | Transgender | Aurum |
| 295119010 | gender role disorder of adolescent or adult | Other/Unknown | Aurum |
| 296515015 | gender identity disorder | Other/Unknown | Aurum |
| 296518018 | [x]other gender identity disorders | Other/Unknown | Aurum |
| 296519014 | [x]gender identity disorder, unspecified | Other/Unknown | Aurum |
| 420552015 | gender reassignment patient | Transgender | Aurum |
| 424018015 | gender identity finding | Other/Unknown | Aurum |
| 1488458017 | gender unknown | Other/Unknown | Aurum |
| 1488459013 | gender unspecified | Other/Unknown | Aurum |
| 2158979015 | transsexual | Transgender | Aurum |
| 2158981018 | male-to-female transsexual | Transgender | Aurum |
| 2158982013 | female-to-male transsexual | Transgender | Aurum |
| 2158983015 | surgically transgendered transsexual, male-to-female | Transgender | Aurum |
| 2158984014 | surgically transgendered transsexual, female-to-male | Transgender | Aurum |
| 3298519013 | male to female transsexual person on hormone therapy | Transgender | Aurum |
| 3298557013 | female to male transsexual person on hormone therapy | Transgender | Aurum |
| 45781000006119 | operations for sexual transformation | Transgender | Aurum |
| 95121000006110 | transsexual | Transgender | Aurum |
| 95131000006113 | trans-sexualism nos | Transgender | Aurum |
| 95141000006115 | trans-sexuality with asexual history | Transgender | Aurum |
| 95151000006118 | trans-sexuality with heterosexual history | Transgender | Aurum |
| 95161000006116 | trans-sexuality with homosexual history | Transgender | Aurum |
| 95171000006111 | trans-sexuality with unspecified sexual history | Transgender | Aurum |
| 379391000006119 | [x]dual-role transvestism | Transgender | Aurum |
| 386831000006112 | [x]fetishistic transvestism | Transgender | Aurum |
| 388001000006110 | gender identity disorder of childhood | Other/Unknown | Aurum |
| 388031000006119 | [x]gender-role disorder nos | Other/Unknown | Aurum |
| 429301000006117 | [x]transsexualism | Transgender | Aurum |
| 877261000006114 | sex transformation operat. nos | Transgender | Aurum |
| 1591631000006115 | gender reassignment patient | Transgender | Aurum |
| 1927781000006117 | gender dysphoria | Other/Unknown | Aurum |
| 2003021000006114 | seen in gender identity clinic | Other/Unknown | Aurum |
| 2007661000006116 | non-binary gender | Other/Unknown | Aurum |
| 2227021000000113 | referral to lgbt (lesbian, gay, bisexual and transgender) service | Other/Unknown | Aurum |
| 2785541000006112 | gender identity disorder of adulthood | Other/Unknown | Aurum |
| 2897321000006117 | gender assignment surgery | Transgender | Aurum |
| 3382951000006118 | adult gender identity disorder, sexually attracted to females | Other/Unknown | Aurum |
| 3437821000006115 | gender identity disorder of adolescence | Other/Unknown | Aurum |
| 3591271000006112 | adult gender identity disorder, sexually attracted to both sexes | Other/Unknown | Aurum |
| 4021031000006114 | gender identity dysphoria | Other/Unknown | Aurum |
| 5697251000006110 | gender identity observations | Other/Unknown | Aurum |
| 6280691000006118 | gender finding | Other/Unknown | Aurum |
| 6746241000006112 | surgically transgendered transsexual | Transgender | Aurum |
| 7088131000006117 | history of gender assignment surgery | Transgender | Aurum |
| 7480251000006114 | previously heterosexual transsexual | Transgender | Aurum |
| 7480261000006111 | previously homosexual transsexual | Transgender | Aurum |
| 7480891000006119 | fetishistic transvestism | Transgender | Aurum |
| 7481051000006110 | dual-role transvestism | Transgender | Aurum |
| 7570821000006117 | male gender | To be determined | Aurum |
| 7570841000006112 | female gender | To be determined | Aurum |
| 7789711000006117 | gender reassignment surgery | Transgender | Aurum |
| 7789731000006111 | operation for sexual transformation | Transgender | Aurum |
| 8367511000006111 | current view provisional problem description item 26 score - gender discomfort issues | Other/Unknown | Aurum |
| 9482161000006111 | transgender | Transgender | Aurum |
| 9482171000006116 | transgender identity | Transgender | Aurum |
| 9482901000006112 | gender dysphoria in childhood | Other/Unknown | Aurum |
| 9482911000006110 | gender identity dysphoria in childhood | Other/Unknown | Aurum |
| 9482931000006116 | gender dysphoria in adolescence and adulthood | Other/Unknown | Aurum |
| 9482941000006114 | gender identity dysphoria in adolescence and adulthood | Other/Unknown | Aurum |
| 12029301000006114 | sexual relationship problem due to gender identity confusion | Other/Unknown | Aurum |
| 12119501000006119 | referral to gender identity clinic | Other/Unknown | Aurum |
| 12119511000006116 | seen in gender identity clinic | Other/Unknown | Aurum |
| 12119521000006112 | did not attend gender identity clinic | Other/Unknown | Aurum |
| 12204951000006110 | gender incongruence | Other/Unknown | Aurum |
| 12622861000006115 | provision of information about nhs population screening programmes available to transgender and non-binary people | Other/Unknown | Aurum |
| 13509261000006115 | non-binary gender | Other/Unknown | Aurum |
| 13934741000006113 | gender confirmation top surgery | Transgender | Aurum |
| 13934761000006112 | gender affirming top surgery | Transgender | Aurum |
| 13934771000006117 | gender confirmation bottom surgery | Transgender | Aurum |
| 13967661000006118 | bilateral mastectomy for female to male transsexual | Transgender | Aurum |
| 13967671000006113 | bilateral mastectomy for transgender male | Transgender | Aurum |
| 13967681000006111 | bilateral mastectomy for ftm (female to male) transsexual | Transgender | Aurum |
| 14090111000006119 | gender affirming surgery | Transgender | Aurum |
| 14146371000006111 | transgender health service | Transgender | Aurum |
| 14683541000006111 | excision of bilateral breasts for female to male transsexual | Transgender | Aurum |
| 14803331000006118 | identifies as nonbinary gender | Other/Unknown | Aurum |
| 14803361000006110 | non-binary gender identity | Other/Unknown | Aurum |
| 14809641000006115 | identifies as female gender | To be determined | Aurum |
| 14809671000006111 | identifies as male gender | To be determined | Aurum |

All codes that were used in exclusion criteria for gender identity in CPRD Aurum and CPRD Gold.

| **Medcode** | **Description** | **Source** |
| --- | --- | --- |
| 15516019 | female pseudohermaphroditism | Aurum |
| 178565013 | male pseudohermaphroditism | Aurum |
| 296538017 | psychological disorder associated with sexual development | Aurum |
| 296540010 | [x]other psychosexual development disorders | Aurum |
| 296541014 | [x]psychosexual development disorder, unspecified | Aurum |
| 314205015 | indeterminate sex or pseudohermaphroditism nos | Aurum |
| 356304015 | pseudohermaphrodite, male with adrenocortical disorder | Aurum |
| 377946011 | false hermaphrodite | Aurum |
| 377947019 | intersex | Aurum |
| 400865010 | indeterminate sex and pseudohermaphroditism | Aurum |
| 400867019 | indeterminate sex nos | Aurum |
| 400868012 | pseudohermaphrodite nos | Aurum |
| 478924017 | true hermaphrodite | Aurum |
| 198371100000611 | pseudohermaphrodite,female with adrenocortical disorder | Aurum |
| 43485100000611 | 46xx true hermaphrodite | Aurum |
| 55051100000611 | chimera 46xx/46xy, true hermaphrodite | Aurum |
| 1847541000006113 | wilms' tumour and nephrotic syndrome with pseudohermaphroditism | Aurum |
| 1906211000006114 | other specified operations for disorders of sex development | Aurum |
| 1906221000006118 | unspecified operations for disorders of sex development | Aurum |
| 1975741000006118 | psychosexual development disorder, unspecified, heterosexuality | Aurum |
| 1975791000006110 | psychosexual development disorder, unspecified, homosexuality | Aurum |
| 1975811000006114 | psychosexual development disorder, unspecified, bisexuality | Aurum |
| 1975861000006112 | psychosexual development disorder, unspecified, other, including prepubertal | Aurum |
| 1975891000006116 | other psychosexual development disorders, heterosexuality | Aurum |
| 1975921000006110 | other psychosexual development disorders, homosexuality | Aurum |
| 1975951000006118 | other psychosexual development disorders, bisexuality | Aurum |
| 1975971000006111 | other psychosexual development disorders, other, including prepubertal | Aurum |
| 2301891000000112 | operations for disorders of sex development | Aurum |
| 2751581000006110 | intersexuality | Aurum |
| 2897311000006113 | intersex surgery | Aurum |
| 3189591000006119 | intersex surgery, female to male | Aurum |
| 3333211000006111 | intersex surgery, male to female | Aurum |
| 3722141000006114 | pseudohermaphroditism | Aurum |
| 4831551000006114 | 46, xx true hermaphrodite | Aurum |
| 8314501000006116 | operations for disorders of sex development | Aurum |
| 11433811000006113 | indeterminate sex | Aurum |
| 14135001000006110 | 46,xx testicular disorder of sex development | Aurum |
| 14800221000006119 | ovotesticular disorder of sex development | Aurum |
| 27395 | indeterminate sex and pseudohermaphroditism | Gold |
| 29508 | indeterminate sex nos | Gold |
| 30414 | [x]other psychosexual development disorders | Gold |
| 41272 | intersex nec | Gold |
| 46264 | true hermaphroditism | Gold |
| 48532 | pseudohermaphrodite nos | Gold |
| 48890 | [x]psychosexual development disorder, unspecified | Gold |
| 49246 | 46xx true hermaphrodite | Gold |
| 50066 | indeterminate sex or pseudohermaphroditism nos | Gold |
| 54216 | male pseudohermaphroditism | Gold |
| 64539 | female pseudohermaphroditism | Gold |
| 93135 | pseudohermaphrodite, male with adrenocortical disorder | Gold |
| 97883 | false hermaphrodite | Gold |
| 108922 | wilms' tumour + nephrotic syndrome + pseudohermaphroditism | Gold |
| 112548 | nephrotic syndrome with pseudohermaphroditism | Gold |
| 113523 | chimera 46xx/46xy, true hermaphrodite | Gold |

## Code list for determining care home residence in CPRD

CPRD Code Browser was used to generate a list of codes, both on CPRD Gold and CPRD Aurum. The search strategy included terms “*care*home*”, “*nursing*home*”, “*convalescent*home*”, “*elder*home*”, “geriatric*home*”, “*hospice*”, and “*old*home*”.

All codes that are related to residing in a care home were considered, including those indicating not residing in a care home. The final code lists had 41 codes for Gold and 84 codes for CPRD Aurum.

Indicator: 0 = Does not reside in care home; 1 = Resides in care home

| **Medcode** | **Description** | **Indicator** | **Source** |
| --- | --- | --- | --- |
| 6991 | patient died in nursing home | 1 | Gold |
| 7101 | seen in old people's home | 1 | Gold |
| 7653 | seen in nursing home | 1 | Gold |
| 10993 | discharge to nursing home | 1 | Gold |
| 11099 | home visit elderly assessment | 0 | Gold |
| 11419 | lives in an old peoples home | 1 | Gold |
| 13359 | lives in a nursing home | 1 | Gold |
| 13360 | nursing/other home | 1 | Gold |
| 13562 | [v]old age home admission medical | 1 | Gold |
| 18929 | full care by hospice | 1 | Gold |
| 22253 | shared care - hospice / gp | 1 | Gold |
| 24816 | residential care | 1 | Gold |
| 24828 | nursing home care | 1 | Gold |
| 27936 | delayed discharge to nursing home | 1 | Gold |
| 32672 | family details and household composition | 1 | Gold |
| 35279 | seen in elderly mentaly infirm home | 1 | Gold |
| 43915 | discharge to private nursing home | 1 | Gold |
| 46222 | place of occurrence of accident/poisoning, old people's home | 1 | Gold |
| 46642 | other residential care homes managed by local authority | 1 | Gold |
| 49138 | [v]delayed discharge - nursing home vacancy awaited | 1 | Gold |
| 53140 | local authority residential care | 1 | Gold |
| 56116 | does not dispose of household rubbish | 1 | Gold |
| 59514 | unable to dispose of household rubbish | 1 | Gold |
| 59548 | lives in an old peoples home | 1 | Gold |
| 59653 | geriatric home admission exam. | 1 | Gold |
| 73083 | nursing home visit note | 1 | Gold |
| 73321 | nursing home | 1 | Gold |
| 94070 | provision of continuing care in nursing home | 1 | Gold |
| 98758 | previously lived in care home | 0 | Gold |
| 101078 | patient died in care home | 1 | Gold |
| 102230 | nursing home acquired pressure ulcer | 1 | Gold |
| 102493 | admission to nursing home | 1 | Gold |
| 102598 | discharge to nursing home | 1 | Gold |
| 107443 | care home visit for initial patient assessment | 1 | Gold |
| 107602 | care home visit for follow-up patient review | 1 | Gold |
| 107757 | care home visit | 1 | Gold |
| 109437 | accident due to fall from burning convalescent home | 1 | Gold |
| 111749 | carbon monoxide fumes from fire in convalescent home | 1 | Gold |
| 111802 | other voluntary or private hospital or nursing home | 1 | Gold |
| 111835 | lives in extra care housing | 1 | Gold |
| 115425 | living temporar in care home | 1 | Gold |
| 71184010 | nursing home | 1 | Aurum |
| 250560019 | lives in a nursing home | 1 | Aurum |
| 250563017 | lives in an old peoples home | 1 | Aurum |
| 264938019 | full care by hospice | 1 | Aurum |
| 283813014 | delayed discharge to nursing home | 1 | Aurum |
| 284635010 | patient died in nursing home | 1 | Aurum |
| 285209012 | seen in nursing home | 1 | Aurum |
| 285465018 | home visit elderly assessment | 0 | Aurum |
| 329630011 | accident due to collapse of burning convalescent home | 1 | Aurum |
| 329644017 | accident due to fall from burning convalescent home | 1 | Aurum |
| 329658015 | hit by object falling from burning convalescent home | 1 | Aurum |
| 329674013 | jump from burning convalescent home | 1 | Aurum |
| 411631010 | seen in old people's home | 1 | Aurum |
| 449801010 | discharge to nursing home | 1 | Aurum |
| 1227760014 | [v]delayed discharge - nursing home vacancy awaited | 1 | Aurum |
| 1227796013 | [v]old age home admission medical | 1 | Aurum |
| 1488495017 | other voluntary or private hospital or nursing home | 1 | Aurum |
| 1488499011 | other residential care homes managed by local authority | 1 | Aurum |
| 1488500019 | other residential care home man voluntary/private agents | 1 | Aurum |
| 3517423018 | transition from acute care to hospice | 1 | Aurum |
| 62241000000112 | nursing home visit note | 1 | Aurum |
| 146841000006119 | shared care - hospice and gp | 1 | Aurum |
| 153661000006114 | seen in elderly mentally infirm home | 1 | Aurum |
| 227981000006119 | place of occurrence of accident or poisoning, old people's home | 1 | Aurum |
| 296351000006117 | nursing or other home | 1 | Aurum |
| 406371000000117 | lives in care home | 1 | Aurum |
| 530521000000114 | provision of continuing care in nursing home | 1 | Aurum |
| 738781000006113 | lives in an old peoples home | 1 | Aurum |
| 801611000006110 | geriatric home admission examination | 1 | Aurum |
| 1009771000006114 | lives in a registered adult care home | 1 | Aurum |
| 1063191000000117 | previously lived in care home | 0 | Aurum |
| 1552711000000110 | patient died in care home | 1 | Aurum |
| 1583741000006116 | provision of continuing care in nursing home | 1 | Aurum |
| 1647741000000115 | nursing home acquired pressure ulcer | 1 | Aurum |
| 1692851000000118 | admission to nursing home | 1 | Aurum |
| 1777721000006111 | activity location: residential care home | 1 | Aurum |
| 1777731000006114 | activity location: nursing home | 1 | Aurum |
| 1778951000006117 | patient died in nhs hospice/specialist palliative care unit | 1 | Aurum |
| 1778961000006115 | patient died in voluntary hospice/specialist palliative care ut. | 1 | Aurum |
| 1851171000006116 | care home assessment required | 1 | Aurum |
| 1851271000006111 | care home assessment not required | 1 | Aurum |
| 1851461000006115 | care home assessment completed | 1 | Aurum |
| 1872471000006118 | accom status - non-mental health registered care home | 1 | Aurum |
| 1872481000006115 | accom status - other accommodation with care and support | 1 | Aurum |
| 1872581000006116 | accom status - nursing home for older persons | 1 | Aurum |
| 1920601000006115 | care home visit for annual patient review | 1 | Aurum |
| 1920611000006117 | care home visit for mid-year patient review | 1 | Aurum |
| 1936201000006117 | admission to hospice from care home | 1 | Aurum |
| 1951031000006114 | discharge to care home | 1 | Aurum |
| 1990711000006115 | no longer lives in a nursing home | 0 | Aurum |
| 2290511000000115 | care home visit | 1 | Aurum |
| 2290551000000116 | care home visit for initial patient assessment | 1 | Aurum |
| 2290591000000112 | care home visit for follow-up patient review | 1 | Aurum |
| 2491051000000119 | lives in extra care housing | 1 | Aurum |
| 3187751000006110 | nh - nursing home | 1 | Aurum |
| 5385771000006114 | old peoples' home | 1 | Aurum |
| 5959551000006111 | discharge to private nursing home | 1 | Aurum |
| 5997731000006114 | private nursing home | 1 | Aurum |
| 6443281000006118 | hospice care assessment | 1 | Aurum |
| 6443301000006119 | hospice care | 1 | Aurum |
| 6443331000006110 | hospice care management | 1 | Aurum |
| 6443341000006117 | manage hospice care | 1 | Aurum |
| 6520541000006113 | mental health residential care hrgs | 1 | Aurum |
| 7224961000006115 | fall in nursing home | 1 | Aurum |
| 8053841000006111 | provision of residential care | 1 | Aurum |
| 8053871000006115 | provision of nursing home care | 1 | Aurum |
| 8053921000006113 | provision of palliative care in hospital, hospice or care home | 1 | Aurum |
| 8053951000006116 | provision of respite care in care home or hospital | 1 | Aurum |
| 8053961000006119 | local authority care home | 1 | Aurum |
| 8053981000006112 | provision of local authority permanent residential care | 1 | Aurum |
| 8054021000006111 | provision of continuing care in hospital or care home | 1 | Aurum |
| 8074901000006115 | patient transfer to nursing home | 1 | Aurum |
| 8137181000006113 | non nhs registered mental health nursing home/residential care | 1 | Aurum |
| 8171261000006117 | non nhs registered nursing home mental health care for older adults - 24 hour not intensive | 1 | Aurum |
| 8178471000006115 | nhs mental health nursing home/residential care | 1 | Aurum |
| 8220191000006110 | residential care | 1 | Aurum |
| 8453631000006112 | home visit requested by care home staff | 1 | Aurum |
| 13072971000006116 | living temporarily in care home | 1 | Aurum |
| 14130291000006116 | died in care home | 1 | Aurum |
| 14130331000006111 | died in nursing home | 1 | Aurum |
| 14281911000006115 | death location type code (actual) : care home with nursing | 1 | Aurum |
| 14281921000006111 | death location type code (actual) : care home without nursing | 1 | Aurum |
| 14282491000006110 | activity location: integrated care home without nursing and care home with nursing | 1 | Aurum |
| 14673881000006114 | nursing home acquired pressure injury | 1 | Aurum |

## Code list for determining unpaid carer responsibilities in CPRD

CPRD Code Browser was used to generate a list of codes, both on CPRD Gold and CPRD Aurum. The search strategy included terms “carer*”, “carin*”, “informal*” and “provid*”.

The returned codes were reviewed in a two-step process. First, all codes that are related to care were considered. Once a list of codes was collated, they were reviewed again in a more stringent manner to flag them as “strict” or “broad”. The final code lists had 28 codes for Gold and 61 codes for CPRD Aurum.

Flag: 1 = Strict; 2 = Broad

| **Medcode** | **Description** | **Flag** | **Source** |
| --- | --- | --- | --- |
| 8499 | Carer | 2 | Gold |
| 24853 | Carer support | 2 | Gold |
| 25634 | Carer support | 2 | Gold |
| 26675 | Carer | 2 | Gold |
| 32264 | Cares for a relative | 1 | Gold |
| 34438 | Carer of a person with learning disability | 1 | Gold |
| 34439 | Carer of a person with physical disability | 1 | Gold |
| 34440 | Carer of a person with sensory impairment | 1 | Gold |
| 34441 | Carer of a person with substance misuse | 1 | Gold |
| 34442 | Carer of a person with alcohol misuse | 1 | Gold |
| 34443 | Carer of a person with chronic disease | 1 | Gold |
| 34444 | Carer of a person with mental health problem | 1 | Gold |
| 46671 | Carer strain index score | 1 | Gold |
| 50111 | Cares for mentally handicapped dependent | 1 | Gold |
| 59672 | Cares for a friend | 1 | Gold |
| 63270 | Cares for a neighbour | 1 | Gold |
| 88987 | Carer of a person with a terminal illness | 1 | Gold |
| 100077 | Cares for a relative | 1 | Gold |
| 101383 | Carer annual health check | 2 | Gold |
| 101419 | Cares for a friend | 1 | Gold |
| 102241 | Cares for a neighbour | 1 | Gold |
| 104380 | Carer of person with dementia | 1 | Gold |
| 106607 | Carer annual health check declined | 2 | Gold |
| 109910 | Carer health check offered | 2 | Gold |
| 110004 | Carer health check | 2 | Gold |
| 110083 | Carer health check completed | 2 | Gold |
| 110250 | Carer health check declined | 2 | Gold |
| 114957 | Carer Support Needs Assessment Tool | 1 | Gold |
| 216663017 | Carer support | 2 | Aurum |
| 283472015 | Care of potential criminal | 2 | Aurum |
| 283494011 | Care of disabled | 2 | Aurum |
| 337526018 | Cares for a friend | 1 | Aurum |
| 337527010 | Cares for a neighbour | 1 | Aurum |
| 344433010 | Carer | 2 | Aurum |
| 397205019 | Care of aged | 2 | Aurum |
| 397206018 | Care of old | 2 | Aurum |
| 412029016 | Cares for mentally handicapped dependent | 1 | Aurum |
| 444639018 | Cares for a relative | 1 | Aurum |
| 1488457010 | Carer | 2 | Aurum |
| 1780196015 | Carer strain index score | 1 | Aurum |
| 2533376016 | Carer of a person with chronic disease | 1 | Aurum |
| 2533378015 | Carer of a person with mental health problem | 1 | Aurum |
| 2533386015 | Carer of a person with alcohol misuse | 1 | Aurum |
| 2533387012 | Carer of a person with substance misuse | 1 | Aurum |
| 2533388019 | Carer of a person with sensory impairment | 1 | Aurum |
| 2533389010 | Carer of a person with physical disability | 1 | Aurum |
| 2533390018 | Carer of a person with learning disability | 1 | Aurum |
| 407261000000118 | Carer of a person with a terminal illness | 1 | Aurum |
| 538491000006118 | Carer | 2 | Aurum |
| 915231000006112 | Carer strain index score | 1 | Aurum |
| 915241000006119 | Carer strain index - 5 points | 1 | Aurum |
| 915251000006117 | Carer strain index - 6 points | 1 | Aurum |
| 915261000006115 | Carer strain index - 7 points | 1 | Aurum |
| 915271000006110 | Carer strain index - 8 points | 1 | Aurum |
| 915281000006113 | Carer strain index - 9 points | 1 | Aurum |
| 915291000006111 | Carer strain index - 10 points | 1 | Aurum |
| 915301000006112 | Carer strain index - 11 points | 1 | Aurum |
| 915311000006110 | Carer strain index - 12 points | 1 | Aurum |
| 915321000006119 | Carer strain index - 13 points | 1 | Aurum |
| 915331000006116 | Carer strain index - 14 points | 1 | Aurum |
| 915341000006114 | Carer strain index - 15 points | 1 | Aurum |
| 915351000006111 | Carer strain index - 16 points | 1 | Aurum |
| 915361000006113 | Carer strain index - 17 points | 1 | Aurum |
| 915371000006118 | Carer strain index - 18 points | 1 | Aurum |
| 915381000006115 | Carer strain index - 19 points | 1 | Aurum |
| 915391000006117 | Carer strain index - 20 points | 1 | Aurum |
| 915401000006115 | Carer strain index - 21 points | 1 | Aurum |
| 915411000006117 | Carer strain index - 22 points | 1 | Aurum |
| 915421000006113 | Carer strain index - 23 points | 1 | Aurum |
| 915431000006111 | Carer strain index - 24 points | 1 | Aurum |
| 915441000006118 | Carer strain index - 25 points | 1 | Aurum |
| 1660441000000116 | Carer annual health check | 2 | Aurum |
| 1786931000006118 | Carer annual health check declined | 2 | Aurum |
| 2003031000006112 | Carer of a person with motor neurone disease | 1 | Aurum |
| 2145441000000117 | Carer of person with dementia | 1 | Aurum |
| 2173661000000118 | Carer annual health check declined | 2 | Aurum |
| 2387241000000111 | Carer Support Needs Assessment Tool | 1 | Aurum |
| 2452961000000115 | Carer health check declined | 2 | Aurum |
| 2453001000000113 | Carer health check offered | 2 | Aurum |
| 2453041000000111 | Carer health check completed | 2 | Aurum |
| 2453121000000117 | Carer health check | 2 | Aurum |
| 4719061000006112 | Caring for the dying | 2 | Aurum |
| 5887411000006119 | Carer stress syndrome | 1 | Aurum |
| 6748741000006115 | Informal carer | 1 | Aurum |
| 8088861000006113 | Cares for dependent relative at home | 1 | Aurum |
| 13788791000006112 | Carer | 2 | Aurum |
| 13820491000006112 | Carer support | 2 | Aurum |
| 13823701000006116 | Carer Strain Index Score | 1 | Aurum |
| 14575381000006112 | Caregiver of dependent with intellectual disability | 1 | Aurum |

## Code list for determining relationship status in CPRD

CPRD Code Browser was used to generate a list of codes, both on CPRD Gold and CPRD Aurum. The search strategy included terms such as “*single*person*”, “*married*”, “*widow*”, “*divorce*”, “*partnership*”, “*spouse*”, etc.

All codes that are related to relationship status were considered. The final code lists had 164 codes for Gold and 232 codes for CPRD Aurum.

For indicator meanings, see 9.1.

| **Medcode** | **Description** | **Legal partnership status** | **Relationship status** | **Source** |
| --- | --- | --- | --- | --- |
| 1135016 | spouse | 1 | 1 | Aurum |
| 1136015 | husband | 1 | 1 | Aurum |
| 1137012 | wife | 1 | 1 | Aurum |
| 65545019 | marital conflict | 1 | 1 | Aurum |
| 84322012 | single | 0 | 0 | Aurum |
| 105113011 | divorce | 3 | 3 | Aurum |
| 129552010 | widower | 4 | 4 | Aurum |
| 169507013 | spouse deceased | 4 | 4 | Aurum |
| 191514016 | married | 1 | 1 | Aurum |
| 191517011 | divorced | 3 | 3 | Aurum |
| 195523014 | separated | 2 | 2 | Aurum |
| 213682015 | alcoholic spouse | 1 | 1 | Aurum |
| 216633014 | death of partner | 0 | 0 | Aurum |
| 250143018 | fh: boyfriend |  | 5 | Aurum |
| 250144012 | fh: girlfriend |  | 5 | Aurum |
| 250169012 | occupation of spouse nos | 1 | 1 | Aurum |
| 250207019 | marital state unknown |  | 6 | Aurum |
| 250209016 | common-law husband |  | 5 | Aurum |
| 250210014 | common-law wife |  | 5 | Aurum |
| 250503019 | homeless single person | 0 | 0 | Aurum |
| 250535019 | divorced couple sharing house | 3 | 3 | Aurum |
| 250583016 | spouse cannot care for patient | 1 | 1 | Aurum |
| 250639016 | marriage | 1 | 1 | Aurum |
| 250645012 | divorce proceedings pending | 3 | 3 | Aurum |
| 250650018 | marital breakdown | 2 | 2 | Aurum |
| 250675019 | battered wife - history | 1 | 1 | Aurum |
| 250676018 | battered husband - history | 1 | 1 | Aurum |
| 250677010 | violent spouse | 1 | 1 | Aurum |
| 250732018 | relationship problems |  | 5 | Aurum |
| 250733011 | boyfriend relationship problem |  | 5 | Aurum |
| 250734017 | girlfriend relationship problem |  | 5 | Aurum |
| 250764013 | extra-marital problems | 1 | 1 | Aurum |
| 250777016 | seven year itch - marital | 1 | 1 | Aurum |
| 250778014 | extra-marital problems nos | 1 | 1 | Aurum |
| 250782011 | new relationship |  | 5 | Aurum |
| 250783018 | first relationship |  | 5 | Aurum |
| 250791010 | partner begins work |  | 5 | Aurum |
| 250792015 | partner stops work |  | 5 | Aurum |
| 250793013 | partner retires |  | 5 | Aurum |
| 250794019 | partner works after retirement |  | 5 | Aurum |
| 250799012 | husband in prison | 1 | 1 | Aurum |
| 250800011 | spouse arrested | 1 | 1 | Aurum |
| 250801010 | boyfriend arrested |  | 5 | Aurum |
| 250802015 | girlfriend arrested |  | 5 | Aurum |
| 250817016 | partner unemployed |  | 5 | Aurum |
| 250833018 | wife pregnant | 1 | 1 | Aurum |
| 250834012 | wife well | 1 | 1 | Aurum |
| 250835013 | wife alive | 1 | 1 | Aurum |
| 250961011 | spouse is handicapped | 1 | 1 | Aurum |
| 250962016 | disabled spouse | 1 | 1 | Aurum |
| 251052016 | widows allowance | 4 | 4 | Aurum |
| 251053014 | widowed mother's allowance | 4 | 4 | Aurum |
| 251054015 | widows pension | 4 | 4 | Aurum |
| 251056018 | war widows pension | 4 | 4 | Aurum |
| 251057010 | widows benefits nos | 4 | 4 | Aurum |
| 251136017 | crime against spouse | 1 | 1 | Aurum |
| 251152019 | spouse works away from home | 1 | 1 | Aurum |
| 253433011 | spouse cooks food | 1 | 1 | Aurum |
| 262935017 | no partner at present | 0 | 0 | Aurum |
| 282957016 | spouse reassured | 1 | 1 | Aurum |
| 285433016 | cohabitee made appointment |  | 5 | Aurum |
| 290530016 | marital reconciliation | 1 | 1 | Aurum |
| 324766016 | battered wife | 1 | 1 | Aurum |
| 324767013 | battered husband | 1 | 1 | Aurum |
| 335218014 | [x]neglect and abandonment, by spouse or partner |  | 5 | Aurum |
| 335225019 | [x]other maltreatment syndromes, by spouse or partner |  | 5 | Aurum |
| 338024013 | divorce proceedings | 3 | 3 | Aurum |
| 389947015 | partner in relationship |  | 5 | Aurum |
| 397721011 | occupation of spouse | 1 | 1 | Aurum |
| 397767011 | broken with partner | 0 | 0 | Aurum |
| 397800018 | spouse unwell | 1 | 1 | Aurum |
| 401444017 | partner had vasectomy |  | 5 | Aurum |
| 405075010 | widows benefits | 4 | 4 | Aurum |
| 411044018 | spouse haemophiliac | 1 | 1 | Aurum |
| 411070017 | occupation of husband | 1 | 1 | Aurum |
| 411071018 | occupation of wife | 1 | 1 | Aurum |
| 411077019 | has infirm partner |  | 5 | Aurum |
| 411878013 | partner sterilised |  | 5 | Aurum |
| 412031013 | looks after chronically sick husband | 1 | 1 | Aurum |
| 412033011 | looks after chronically sick spouse | 1 | 1 | Aurum |
| 412034017 | looks after chronically sick wife | 1 | 1 | Aurum |
| 412070019 | partnership problems |  | 5 | Aurum |
| 412087017 | legal problem with divorce | 3 | 3 | Aurum |
| 412088010 | divorce problems | 3 | 3 | Aurum |
| 412089019 | spouse returned home | 1 | 1 | Aurum |
| 412099012 | wife committed adultery | 1 | 1 | Aurum |
| 412100016 | husband committed adultery | 1 | 1 | Aurum |
| 412102012 | row with wife | 1 | 1 | Aurum |
| 412103019 | spouse unsympathetic | 1 | 1 | Aurum |
| 412104013 | spouse inattentive | 1 | 1 | Aurum |
| 412131011 | separated from cohabitee | 0 | 0 | Aurum |
| 412132016 | husband left home | 2 | 2 | Aurum |
| 412133014 | wife left home | 2 | 2 | Aurum |
| 412134015 | cohabitee left home | 0 | 0 | Aurum |
| 412135019 | spouse left home | 2 | 2 | Aurum |
| 415853015 | remarried | 1 | 1 | Aurum |
| 415854014 | newly wed | 1 | 1 | Aurum |
| 418923013 | husband alcoholic | 1 | 1 | Aurum |
| 442161011 | cohabitee returned |  | 5 | Aurum |
| 442258018 | husband died | 4 | 4 | Aurum |
| 443775016 | wife unable to cope | 1 | 1 | Aurum |
| 456915015 | relationship counselling |  | 5 | Aurum |
| 460520016 | artificial insemination by husband | 1 | 1 | Aurum |
| 460981013 | [v]marital problems | 1 | 1 | Aurum |
| 476009017 | common law partnership |  | 5 | Aurum |
| 483247015 | spouse committed infidelity | 1 | 1 | Aurum |
| 483248013 | spouse committed adultery | 1 | 1 | Aurum |
| 485552011 | widowed | 4 | 4 | Aurum |
| 486188017 | maladjustment to married life | 1 | 1 | Aurum |
| 487634016 | cohabiting |  | 5 | Aurum |
| 500032013 | marital problems | 1 | 1 | Aurum |
| 1207808017 | marital counselling | 1 | 1 | Aurum |
| 1227741019 | [v]divorce | 3 | 3 | Aurum |
| 1227797016 | [v]marriage medical | 1 | 1 | Aurum |
| 1484966014 | death of wife | 4 | 4 | Aurum |
| 1485014011 | partner dying |  | 5 | Aurum |
| 1488623014 | cohabitee |  | 5 | Aurum |
| 2159175019 | informing partner |  | 5 | Aurum |
| 2470673016 | lives with partner |  | 5 | Aurum |
| 2575940016 | death of husband | 4 | 4 | Aurum |
| 2881789016 | lives with spouse | 1 | 1 | Aurum |
| 53381000006116 | widows allowances | 4 | 4 | Aurum |
| 53421000006114 | widows pensions | 4 | 4 | Aurum |
| 142341000006112 | single person | 0 | 0 | Aurum |
| 239861000006111 | questionable if patient marital status correct |  | 6 | Aurum |
| 239871000006116 | patient marital status unknown |  | 6 | Aurum |
| 298041000000110 | breast lump detected by partner |  | 5 | Aurum |
| 300231000000115 | low sperm count in partner |  | 5 | Aurum |
| 300251000000110 | fertility problems in partner |  | 5 | Aurum |
| 312241000000110 | partner is informal carer |  | 5 | Aurum |
| 493941000006111 | artificial insemin by husband | 1 | 1 | Aurum |
| 493971000000110 | married/civil partner | 1 | 1 | Aurum |
| 494031000000110 | divorced/person whose civil partnership has been dissolved | 3 | 3 | Aurum |
| 494151000000119 | widowed/surviving civil partner | 4 | 4 | Aurum |
| 494211000000114 | marital/civil state not disclosed |  | 6 | Aurum |
| 562571000000116 | partner pregnant |  | 5 | Aurum |
| 717161000006113 | marital discord | 1 | 1 | Aurum |
| 717171000006118 | marital disharmony | 1 | 1 | Aurum |
| 717221000006112 | marital stress | 1 | 1 | Aurum |
| 717231000006110 | marital trouble | 1 | 1 | Aurum |
| 738831000000111 | partner had tubal ligation |  | 5 | Aurum |
| 759611000000114 | antenatal screening not indicated for partner |  | 5 | Aurum |
| 759671000000116 | antenatal screening for partner required |  | 5 | Aurum |
| 759851000000112 | partner declined antenatal screening |  | 5 | Aurum |
| 852911000006116 | mad wife | 1 | 1 | Aurum |
| 853431000006111 | sexual problem within marriage | 1 | 1 | Aurum |
| 923471000006119 | marital counselling | 1 | 1 | Aurum |
| 981081000006118 | physically/emotionally abusive to partner |  | 5 | Aurum |
| 982451000006113 | relationship breakdown | 0 | 0 | Aurum |
| 1009721000006113 | lives independently with spouse/partner |  | 5 | Aurum |
| 1539061000006113 | new partner male |  | 5 | Aurum |
| 1551151000006110 | intrauterine insemination with superovulation using partner sperm |  | 5 | Aurum |
| 1551171000006117 | intrauterine insemination without superovulation using partner sperm |  | 5 | Aurum |
| 1661521000000116 | partner contraception |  | 5 | Aurum |
| 1672431000006114 | common law partnership |  | 5 | Aurum |
| 1672441000006116 | homosexual marriage, female | 1 | 1 | Aurum |
| 1672451000006119 | homosexual marriage, male | 1 | 1 | Aurum |
| 1716541000000113 | knows partner's method of contraception |  | 5 | Aurum |
| 1747781000006111 | concern expressed by spouse | 1 | 1 | Aurum |
| 1748001000006118 | patient aware of partner's method of contraception |  | 5 | Aurum |
| 1757711000006118 | patient in a relationship |  | 5 | Aurum |
| 1804931000006113 | in de facto relationship |  | 5 | Aurum |
| 1804951000006118 | marital or partnership status inadequately described |  | 6 | Aurum |
| 1817361000006114 | [x]maltreatment, by spouse or partner |  | 5 | Aurum |
| 1834051000006111 | male partner |  | 5 | Aurum |
| 1834061000006113 | female partner |  | 5 | Aurum |
| 1998361000006115 | polygamous partner |  | 5 | Aurum |
| 2406051000000112 | wife unwell | 1 | 1 | Aurum |
| 2406671000000114 | wife deceased | 4 | 4 | Aurum |
| 2406841000000116 | husband well | 1 | 1 | Aurum |
| 2406881000000112 | husband alive | 1 | 1 | Aurum |
| 2406921000000118 | husband unwell | 1 | 1 | Aurum |
| 2406971000000119 | husband deceased | 4 | 4 | Aurum |
| 2407071000000118 | partner deceased | 0 | 0 | Aurum |
| 2407111000000112 | partner alive |  | 5 | Aurum |
| 2407151000000111 | partner unwell |  | 5 | Aurum |
| 2407191000000115 | partner well |  | 5 | Aurum |
| 3090441000006111 | legally married | 1 | 1 | Aurum |
| 3090451000006113 | legal marriage | 1 | 1 | Aurum |
| 3392411000006112 | engaged to be married |  | 5 | Aurum |
| 4064611000006116 | psychologically abused spouse | 1 | 1 | Aurum |
| 4082271000006115 | abusive emotional relationship |  | 5 | Aurum |
| 4082291000006119 | abusive emotional relationship with spouse | 1 | 1 | Aurum |
| 4118631000006112 | personal relationship breakdown | 0 | 0 | Aurum |
| 4118641000006119 | relationship breakdown | 0 | 0 | Aurum |
| 4380341000006118 | single, never married | 0 | 0 | Aurum |
| 4535281000006117 | spouse unable to care for patient | 1 | 1 | Aurum |
| 4535671000006118 | marriage, life event | 1 | 1 | Aurum |
| 4537501000006116 | widows payment | 4 | 4 | Aurum |
| 4923521000006113 | number of previous marriages |  | 6 | Aurum |
| 4924501000006118 | impending marriage |  | 5 | Aurum |
| 4924521000006111 | recent marriage | 1 | 1 | Aurum |
| 4924531000006114 | recent marriage, life event | 1 | 1 | Aurum |
| 4926671000006118 | wife arrested | 1 | 1 | Aurum |
| 4926681000006115 | husband arrested | 1 | 1 | Aurum |
| 4942401000006113 | same generation sex partner |  | 5 | Aurum |
| 4980411000006116 | committed sexual relationship |  | 5 | Aurum |
| 4980431000006110 | heterosexual relationship |  | 5 | Aurum |
| 4980441000006117 | homosexual relationship |  | 5 | Aurum |
| 5170631000006111 | abuse of partner |  | 5 | Aurum |
| 5448541000006117 | partner |  | 5 | Aurum |
| 5887191000006116 | reconciliation with partner |  | 5 | Aurum |
| 5888151000006114 | disharmony with partner |  | 5 | Aurum |
| 5903761000006111 | employment circumstances of partner or spouse |  | 5 | Aurum |
| 5940491000006114 | under care of marriage guidance counsellor | 1 | 1 | Aurum |
| 5942941000006113 | seen by marriage guidance counsellor | 1 | 1 | Aurum |
| 5947001000006113 | referral by marriage guidance counsellor | 1 | 1 | Aurum |
| 5953701000006115 | referral to marriage guidance counsellor | 1 | 1 | Aurum |
| 6025471000006110 | relationship counseling |  | 5 | Aurum |
| 6557191000006111 | cancer diagnosis discussed with partner in relationship |  | 5 | Aurum |
| 6557241000006111 | cancer care plan discussed with partner in relationship |  | 5 | Aurum |
| 6557381000006110 | preferred place of death discussed with partner in relationship |  | 5 | Aurum |
| 6767621000006110 | lives with husband | 1 | 1 | Aurum |
| 6853791000006115 | failure to conceive due to infertility of male partner |  | 5 | Aurum |
| 6912231000006116 | victim of abusive sexual relationship |  | 5 | Aurum |
| 6995701000006114 | physically abusive to partner |  | 5 | Aurum |
| 7058321000006111 | intrauterine insemination without controlled ovarian hyperstimulation using partner sperm |  | 5 | Aurum |
| 7067061000006113 | intrauterine insemination with controlled ovarian hyperstimulation using partner sperm |  | 5 | Aurum |
| 7124071000006114 | legally separated with interlocutory decree | 2 | 2 | Aurum |
| 7289791000006116 | spouse of subject is victim of crime | 1 | 1 | Aurum |
| 7342331000006113 | lives with wife | 1 | 1 | Aurum |
| 7691581000006111 | health of partner |  | 5 | Aurum |
| 8033011000006119 | problem with aged spouse or partner |  | 5 | Aurum |
| 8111491000006116 | marital psychotherapy | 1 | 1 | Aurum |
| 8262701000006112 | victim of forced marriage | 1 | 1 | Aurum |
| 11927261000006115 | death of husband | 4 | 4 | Aurum |
| 12716291000006112 | fh: boyfriend |  | 5 | Aurum |
| 12716301000006113 | fh: girlfriend |  | 5 | Aurum |
| 13932191000006111 | partner had vasectomy |  | 5 | Aurum |
| 14130591000006118 | alcoholic husband | 1 | 1 | Aurum |
| 14130601000006114 | alcoholic spouse | 1 | 1 | Aurum |
| 14842121000006116 | relationship problem |  | 5 | Aurum |
| 30597 | [v]artificial insemination from husband | 1 | 1 | Gold |
| 23858 | [v]divorce | 3 | 3 | Gold |
| 9551 | [v]marital problems | 1 | 1 | Gold |
| 46941 | [v]marriage medical | 1 | 1 | Gold |
| 104936 | [x]maltreatment, by spouse or partner |  | 5 | Gold |
| 37113 | [x]neglect and abandonment, by spouse or partner |  | 5 | Gold |
| 28484 | [x]other maltreatment syndromes, by spouse or partner |  | 5 | Gold |
| 10330 | alcoholic spouse | 1 | 1 | Gold |
| 104214 | antenatal screening for partner required |  | 5 | Gold |
| 97398 | antenatal screening not indicated for partner |  | 5 | Gold |
| 16344 | artificial insemin by husband | 1 | 1 | Gold |
| 23508 | battered husband | 1 | 1 | Gold |
| 42454 | battered husband - history | 1 | 1 | Gold |
| 3321 | battered wife | 1 | 1 | Gold |
| 22873 | battered wife - history | 1 | 1 | Gold |
| 42390 | boyfriend arrested |  | 5 | Gold |
| 4531 | boyfriend relationship problem |  | 5 | Gold |
| 61291 | breast lump detected by partner |  | 5 | Gold |
| 24055 | broken with partner | 0 | 0 | Gold |
| 98130 | cohabitee |  | 5 | Gold |
| 42428 | cohabitee left home | 0 | 0 | Gold |
| 39474 | cohabitee made appointment |  | 5 | Gold |
| 38325 | cohabitee returned |  | 5 | Gold |
| 25503 | cohabiting |  | 5 | Gold |
| 104879 | common law partnership |  | 5 | Gold |
| 16315 | common-law husband |  | 5 | Gold |
| 42386 | common-law wife |  | 5 | Gold |
| 50149 | crime against spouse | 1 | 1 | Gold |
| 12325 | death of husband | 4 | 4 | Gold |
| 25097 | death of husband | 4 | 4 | Gold |
| 28440 | death of partner | 0 | 0 | Gold |
| 207 | death of spouse | 4 | 4 | Gold |
| 11251 | death of wife | 4 | 4 | Gold |
| 15824 | disabled spouse | 1 | 1 | Gold |
| 94555 | discharge by marriage guidance counsellor | 1 | 1 | Gold |
| 6056 | divorce | 3 | 3 | Gold |
| 9910 | divorce problems | 3 | 3 | Gold |
| 838 | divorce proceedings | 3 | 3 | Gold |
| 2159 | divorce proceedings pending | 3 | 3 | Gold |
| 1522 | divorced | 3 | 3 | Gold |
| 31678 | divorced couple sharing house | 3 | 3 | Gold |
| 96856 | divorced/person whose civil partnership has been dissolved | 3 | 3 | Gold |
| 34771 | extra-marital problems | 1 | 1 | Gold |
| 37551 | extra-marital problems nos | 1 | 1 | Gold |
| 45005 | fertility problems in partner |  | 5 | Gold |
| 44353 | fh: boyfriend |  | 5 | Gold |
| 30986 | fh: girlfriend |  | 5 | Gold |
| 50485 | first relationship |  | 5 | Gold |
| 68095 | girlfriend arrested |  | 5 | Gold |
| 3111 | girlfriend relationship problem |  | 5 | Gold |
| 22336 | has infirm partner |  | 5 | Gold |
| 21860 | health of spouse | 1 | 1 | Gold |
| 25452 | homeless single person | 0 | 0 | Gold |
| 59817 | husband | 1 | 1 | Gold |
| 15404 | husband alcoholic | 1 | 1 | Gold |
| 109946 | husband alive | 1 | 1 | Gold |
| 21433 | husband committed adultery | 1 | 1 | Gold |
| 109964 | husband deceased | 4 | 4 | Gold |
| 12076 | husband died | 4 | 4 | Gold |
| 17802 | husband died | 4 | 4 | Gold |
| 24769 | husband in prison | 1 | 1 | Gold |
| 5055 | husband left home | 2 | 2 | Gold |
| 109680 | husband unwell | 1 | 1 | Gold |
| 109828 | husband well | 1 | 1 | Gold |
| 32451 | informing partner |  | 5 | Gold |
| 91652 | intrauterine insemination superovulation partner sperm |  | 5 | Gold |
| 111544 | intrauterine insemination without superovulat partner sperm |  | 5 | Gold |
| 103033 | knows partner's method of contraception |  | 5 | Gold |
| 20313 | legal problem with divorce | 3 | 3 | Gold |
| 108175 | lives with partner |  | 5 | Gold |
| 110375 | lives with spouse | 1 | 1 | Gold |
| 16552 | looks after chronically sick husband | 1 | 1 | Gold |
| 15115 | looks after chronically sick spouse | 1 | 1 | Gold |
| 20149 | looks after chronically sick wife | 1 | 1 | Gold |
| 49666 | low sperm count in partner |  | 5 | Gold |
| 15313 | maladjustment to married life | 1 | 1 | Gold |
| 954 | marital breakdown | 2 | 2 | Gold |
| 333 | marital conflict | 1 | 1 | Gold |
| 4565 | marital counselling | 1 | 1 | Gold |
| 3551 | marital discord | 1 | 1 | Gold |
| 1540 | marital disharmony | 1 | 1 | Gold |
| 1349 | marital problems | 1 | 1 | Gold |
| 29543 | marital reconciliation | 1 | 1 | Gold |
| 47408 | marital state unknown |  | 6 | Gold |
| 723 | marital stress | 1 | 1 | Gold |
| 29544 | marital trouble | 1 | 1 | Gold |
| 100473 | marital/civil state not disclosed |  | 6 | Gold |
| 7869 | marriage | 1 | 1 | Gold |
| 3988 | married | 1 | 1 | Gold |
| 88373 | married/civil partner | 1 | 1 | Gold |
| 9112 | new relationship |  | 5 | Gold |
| 7419 | newly wed | 1 | 1 | Gold |
| 13001 | no partner at present | 0 | 0 | Gold |
| 33001 | occupation of husband | 1 | 1 | Gold |
| 36077 | occupation of spouse | 1 | 1 | Gold |
| 54096 | occupation of spouse nos | 1 | 1 | Gold |
| 39292 | occupation of wife | 1 | 1 | Gold |
| 99328 | partner begins work |  | 5 | Gold |
| 101904 | partner contraception |  | 5 | Gold |
| 110227 | partner deceased | 0 | 0 | Gold |
| 113149 | partner declined antenatal screening |  | 5 | Gold |
| 36947 | partner dying |  | 5 | Gold |
| 97076 | partner had tubal ligation |  | 5 | Gold |
| 1328 | partner had vasectomy |  | 5 | Gold |
| 102413 | partner in relationship |  | 5 | Gold |
| 60723 | partner is informal carer |  | 5 | Gold |
| 94917 | partner pregnant |  | 5 | Gold |
| 109323 | partner retires |  | 5 | Gold |
| 22934 | partner sterilised |  | 5 | Gold |
| 47411 | partner stops work |  | 5 | Gold |
| 33188 | partner unemployed |  | 5 | Gold |
| 109906 | partner unwell |  | 5 | Gold |
| 110148 | partner well |  | 5 | Gold |
| 101900 | partner works after retirement |  | 5 | Gold |
| 22909 | partnership problems |  | 5 | Gold |
| 109484 | pat. marital status ? correct |  | 6 | Gold |
| 93992 | pat. marital status unknown |  | 6 | Gold |
| 28985 | referral to marriage guidance counsellor | 1 | 1 | Gold |
| 25699 | relationship counselling |  | 5 | Gold |
| 6104 | relationship problems |  | 5 | Gold |
| 1580 | remarried | 1 | 1 | Gold |
| 30950 | row with wife | 1 | 1 | Gold |
| 36333 | seen by marriage guidance counsellor | 1 | 1 | Gold |
| 4204 | separated | 2 | 2 | Gold |
| 27385 | separated from cohabitee | 0 | 0 | Gold |
| 21925 | seven year itch - marital | 1 | 1 | Gold |
| 37265 | single | 0 | 0 | Gold |
| 11103 | single - unmarried | 0 | 0 | Gold |
| 95101 | spouse | 1 | 1 | Gold |
| 42398 | spouse arrested | 1 | 1 | Gold |
| 23385 | spouse cannot care for patient | 1 | 1 | Gold |
| 39651 | spouse committed adultery | 1 | 1 | Gold |
| 15777 | spouse committed infidelity | 1 | 1 | Gold |
| 32984 | spouse cooks food | 1 | 1 | Gold |
| 63118 | spouse haemophiliac | 1 | 1 | Gold |
| 54816 | spouse inattentive | 1 | 1 | Gold |
| 41203 | spouse is handicapped | 1 | 1 | Gold |
| 15020 | spouse left home | 2 | 2 | Gold |
| 21346 | spouse reassured | 1 | 1 | Gold |
| 15950 | spouse returned home | 1 | 1 | Gold |
| 42321 | spouse unsympathetic | 1 | 1 | Gold |
| 3719 | spouse unwell | 1 | 1 | Gold |
| 42402 | spouse works away from home | 1 | 1 | Gold |
| 38003 | under care of marriage guidance counsellor | 1 | 1 | Gold |
| 3483 | violent spouse | 1 | 1 | Gold |
| 98610 | war widows pension | 4 | 4 | Gold |
| 4312 | widowed | 4 | 4 | Gold |
| 94044 | widowed/surviving civil partner | 4 | 4 | Gold |
| 15527 | widower | 4 | 4 | Gold |
| 98818 | widows allowance | 4 | 4 | Gold |
| 61509 | widows allowances | 4 | 4 | Gold |
| 40866 | widows benefits | 4 | 4 | Gold |
| 60821 | widows benefits nos | 4 | 4 | Gold |
| 45010 | widows pension | 4 | 4 | Gold |
| 33000 | widows pensions | 4 | 4 | Gold |
| 59829 | wife | 1 | 1 | Gold |
| 17538 | wife alive | 1 | 1 | Gold |
| 42400 | wife committed adultery | 1 | 1 | Gold |
| 109892 | wife deceased | 4 | 4 | Gold |
| 4925 | wife left home | 2 | 2 | Gold |
| 8470 | wife pregnant | 1 | 1 | Gold |
| 23514 | wife unable to cope | 1 | 1 | Gold |
| 109775 | wife unwell | 1 | 1 | Gold |
| 23974 | wife well | 1 | 1 | Gold |

# Abbreviations

CPRD Clinical Practice Research Datalink

E England

NI Northern Ireland

NISRA Northern Ireland Statistics and Research Agency

ONS Office for National Statistics

S Scotland

UK United Kingdom (including England, Wales, Northern Ireland, and Scotland)

W Wales

# References

1. Nomis/Office for National Statistics. Census 2011. 2014 [cited 2023 Oct 3]. QS203UK Country of birth. Available from: https://www.nomisweb.co.uk/census/2011/qs203uk

2. Nomis/Office for National Statistics. Census 2021. 2022 [cited 2023 Oct 16]. TS004 - Country of birth. Available from: https://www.nomisweb.co.uk/datasets/c2021ts004

3. Northern Ireland Statistics and Research Agency. Census 2021 main statistics demography tables – country of birth. 2022 [cited 2023 Oct 16]. MS-A16 Country of birth - basic detail. Available from: https://www.nisra.gov.uk/publications/census-2021-main-statistics-demography-tables-country-of-birth

4. Scotland’s Census. UV204 - Country of birth [Internet]. 2024 [cited 2025 Dec 8]. Available from: https://statistics.ukdataservice.ac.uk/dataset/scotland-s-census-2022-uv204-country-of-birth

5. Nomis/Office for National Statistics. Census 2011. 2013 [cited 2023 Oct 16]. QS204EW Main language (detailed). Available from: https://www.nomisweb.co.uk/census/2011/qs204ew

6. Northern Ireland Statistics and Research Agency. 2011 Census key statistics tables on ethnicity, identity, language and religion. 2022 [cited 2023 Oct 16]. KS207NI Main language. Available from: https://www.nisra.gov.uk/publications/2011-census-key-statistics-tables-ethnicity-identity-language-and-religion

7. Scotland’s Census. Census 2011. 2013 [cited 2023 Oct 16]. Table KS206SC - Language. Available from: https://www.scotlandscensus.gov.uk/webapi/jsf/tableView/tableView.xhtml

8. Nomis/Office for National Statistics. Census 2021. 2022 [cited 2023 Oct 16]. TS024 - Main language. Available from: https://www.nomisweb.co.uk/datasets/c2021ts024

9. Northern Ireland Statistics and Research Agency. Census 2021 main statistics language tables. 2022 [cited 2023 Oct 16]. MS-B13 Main language - full detail. Available from: https://www.nisra.gov.uk/publications/census-2021-main-statistics-language-tables

10. Scotland’s Census. UV212 - Main language [Internet]. 2024 [cited 2025 Dec 8]. Available from: https://statistics.ukdataservice.ac.uk/dataset/scotland-s-census-2022-uv212-main-language

11. Nomis/Office for National Statistics. Census 2011. 2013 [cited 2023 Oct 3]. QS208EW Religion. Available from: https://www.nomisweb.co.uk/census/2011/qs208ew

12. Northern Ireland Statistics and Research Agency. 2011 Census key statistics tables on ethnicity, identity, language and religion. 2012 [cited 2023 Oct 3]. KS211NI Religion. Available from: https://www.nisra.gov.uk/publications/2011-census-key-statistics-tables-ethnicity-identity-language-and-religion

13. Scotland’s Census. 2011 census table data: Scotland. 2021 [cited 2023 Oct 3]. Table KS209SCb - Religion. Available from: https://www.scotlandscensus.gov.uk/documents/2011-census-table-data-scotland/

14. Nomis/Office for National Statistics. Census 2021. 2022 [cited 2023 Oct 16]. TS030 - Religion. Available from: https://www.nomisweb.co.uk/datasets/c2021ts030

15. Northern Ireland Statistics and Research Agency. Census 2021 main statistics religion tables. 2022 [cited 2023 Oct 16]. MS-B21 Religion - full detail. Available from: https://www.nisra.gov.uk/publications/census-2021-main-statistics-religion-tables

16. Scotland’s Census. UV205 - Religion [Internet]. 2024 [cited 2025 Dec 8]. Available from: https://statistics.ukdataservice.ac.uk/dataset/scotland-s-census-2022-uv205-religion

17. Nomis/Office for National Statistics. Census 2021. 2022 [cited 2023 Oct 16]. TS079 - Sexual orientation. Available from: https://www.nomisweb.co.uk/datasets/c2021ts079

18. Northern Ireland Statistics and Research Agency. Census 2021 main statistics sexual orientation tables. 2023 [cited 2023 Oct 16]. MS-C03 Sexual orientation - full detail. Available from: https://www.nisra.gov.uk/publications/census-2021-main-statistics-sexual-orientation-tables

19. Scotland’s Census. UV904 - Sexual Orientation [Internet]. 2024 [cited 2025 Dec 8]. Available from: https://statistics.ukdataservice.ac.uk/dataset/scotland-s-census-2022-uv904-sexual-orientation

20. Nomis/Office for National Statistics. Census 2021. 2022 [cited 2023 Oct 16]. TS070 - Gender identity. Available from: https://www.nomisweb.co.uk/datasets/c2021ts070

21. Scotland’s Census. UV903a - Trans Status or History (7 Groups) [Internet]. 2024 [cited 2025 Dec 8]. Available from: https://statistics.ukdataservice.ac.uk/dataset/scotland-s-census-2022-uv903a-trans-status-or-history-7-groups

22. Nomis/Office for National Statistics. Census 2011. 2014 [cited 2023 Oct 3]. KS405UK Communal establishment residents. Available from: https://www.nomisweb.co.uk/census/2011/ks405uk

23. Nomis/Office for National Statistics. Census 2021. 2022 [cited 2023 Oct 16]. TS048 - Communal establishment management and type. Available from: https://www.nomisweb.co.uk/datasets/c2021ts048

24. Northern Ireland Statistics and Research Agency. Census 2021 main statistics communal establishment tables. 2023 [cited 2023 Oct 16]. MS-F02 Communal establishment management and type- usual residents in a communal establishment. Available from: https://www.nisra.gov.uk/publications/census-2021-main-statistics-communal-establishment-tables

25. Scotland’s Census. UV414 - Communal establishment management and type - Residents [Internet]. 2024 [cited 2025 Dec 18]. Available from: https://statistics.ukdataservice.ac.uk/dataset/scotland-s-census-2022-uv414-communal-establishment-management-and-type-residents

26. Nomis/Office for National Statistics. Census 2011. 2014 [cited 2023 Oct 3]. QS301UK Provision of unpaid care. Available from: https://www.nomisweb.co.uk/census/2011/qs301uk

27. Nomis/Office for National Statistics. Census 2021. 2022 [cited 2023 Oct 16]. TS039 - Provision of unpaid care. Available from: https://www.nomisweb.co.uk/datasets/c2021ts039

28. Northern Ireland Statistics and Research Agency. Census 2021 main statistics health, disability and unpaid care tables. 2022 [cited 2023 Oct 16]. MS-D17 Provision of unpaid care by broad age bands. Available from: https://www.nisra.gov.uk/publications/census-2021-main-statistics-health-disability-and-unpaid-care-tables

29. Scotland’s Census. UV301 - Provision of unpaid care [Internet]. 2024 [cited 2025 Dec 8]. Available from: https://statistics.ukdataservice.ac.uk/dataset/scotland-s-census-2022-uv301-provision-of-unpaid-care

30. Nomis/Office for National Statistics. Census 2011. 2014 [cited 2023 Oct 3]. KS103UK Marital and civil partnership status. Available from: https://www.nomisweb.co.uk/census/2011/ks103uk

31. Nomis/Office for National Statistics. Census 2021. 2022 [cited 2023 Oct 16]. TS002 - Legal partnership status. Available from: https://www.nomisweb.co.uk/datasets/c2021ts002

32. Northern Ireland Statistics and Research Agency. Census 2021 main statistics demography tables – household relationships. 2023 [cited 2024 Apr 30]. MS-A30 Marital and civil partnership status [UPDATED]. Available from: https://www.nisra.gov.uk/publications/census-2021-main-statistics-demography-tables-household-relationships

33. Scotland’s Census. UV104 - Marital and civil partnership status [Internet]. 2024 [cited 2025 Dec 8]. Available from: https://statistics.ukdataservice.ac.uk/dataset/scotland-s-census-2022-uv104-marital-and-civil-partnership-status
